# Indirect inhibition of the NLRP3-interleukin-1β axis contributes to the efficacy of JAK1 inhibitors in experimental colitis and human ulcerative colitis

**DOI:** 10.1038/s41467-026-71808-y

**Published:** 2026-04-18

**Authors:** Beibei Liu, Marianne R. Spalinger, Annalisa Invernizzi, Eike Gerdes, Babett Steglich, Marlene Schwarzfischer, Andres Machicote, Penelope Pelczar, Doris Pöhlmann, Mikolaj Nawrocki, Marius Böttcher, Ayob Aleko, Lis Noelia Velasquez, Sandra Wende, Franziska Stallbaum, Justus Neuendorff, Saskia Grosshauser, Franziska Muscate, Gemma Douilhet, Laura Garcia Perez, Morsal Sabihi, Katharina Möller, Florian Viehweger, Jan P. Sutter, Christoph Kilian, Yogesh Kumar, Thorben Fründt, Guido Sauter, Thomas Rösch, Nicola Gagliani, Amedeo Caflisch, Michael Scharl, Samuel Huber

**Affiliations:** 1 Section of Molecular Immunology and Gastroenterology, I. Department of Medicine, University Medical Center Hamburg-Eppendorf, Hamburg, Germany; 2 Hamburg Center for Translational Immunology (HCTI), University Medical Center Hamburg-Eppendorf, Hamburg, Germany; 3 Department of Gastroenterology and Hepatology, University Hospital Zurich, University of Zurich, Zurich, Switzerland; 4 Department of Biochemistry, University of Zurich, Zurich, Switzerland; 5 Theoretical Ultrafast X-ray Science, Deutsches Elektronen-Synchrotron DESY, Hamburg, Germany; 6 Institute of Pathology, University Medical Center Hamburg-Eppendorf, Hamburg, Germany; 7 Department for Interdisciplinary Endoscopy, University Medical Center Hamburg-Eppendorf, Hamburg, Germany; 8 Institute for Inflammation and Carcinogenesis, University Medical Center Hamburg-Eppendorf, Hamburg, Germany

**Keywords:** Inflammatory bowel disease, Inflammasome, Mucosal immunology, Innate immunity

## Abstract

Tofacitinib, a pan–Janus kinase inhibitor, and the Janus kinase 1–preferential inhibitors Upadacitinib and Filgotinib are approved for the treatment of ulcerative colitis, yet their molecular mechanisms of action remain incompletely understood. Here, using dextran sulfate sodium–induced and T cell transfer colitis models together with analyses of individuals with ulcerative colitis, we show that all three inhibitors ameliorate colitis in mice with macrophage-specific deletion of protein tyrosine phosphatase non-receptor type 2, a model characterized by hyperactive Janus kinase–signal transducer and activator of transcription signaling. In contrast, only Upadacitinib and Filgotinib provide enhanced protection in wild-type mice – an effect that is lost upon genetic disruption of inflammasome signaling. Longitudinal single-cell transcriptomic analyses and immunostaining of intestinal biopsies further show that Upadacitinib reduces interleukin-1β expression in vivo, which associates with clinical response. Thus, indirect suppression of inflammasome activity contributes to the efficacy of Janus kinase 1–preferential inhibitors.

## Abstract (web summary)

The Janus Kinase inhibitors Tofacitinib, Upadacitinib and Filgotinib are used for the treatment of ulcerative colitis but the full mechanism of their action is not completely elucidated. Here the authors show Tofacitinib, Upadacitinib and Filgotinib inhibit JAK/STAT signaling, but also show Upadacitinib and Filgotinib additionally inhibit NLRP3 inflammasome and IL-1β production in experimental and human colitis.

## Introduction

Ulcerative colitis (UC) is a subtype of inflammatory bowel disease (IBD) and is characterized by a continuous, relapsing colonic inflammation. The pathogenesis of UC is considered multifactorial, including genetic, environmental, microbial, and immune components^1–3^. The key therapeutic goal of UC is the induction and maintenance of long-term remission, which is defined by clinical symptoms, mucosal healing and deep remission based on histology^4^. Of note, mucosal healing is associated with a better clinical outcome and a reduced risk of developing colorectal cancer^5^. Biological therapies, including antibodies against cytokines (e.g., TNF, IL-12p40 and IL-12p19) or integrins (e.g., alpha4beta7), have improved outcomes for patients with UC^6^. However, despite the advances in medical therapy, a substantial proportion of patients do not respond to the available treatments, lose response over time, or have adverse events^7^.

Pro-inflammatory cytokines play a key role in IBD. IL-1β, a pro-inflammatory cytokine downstream of the inflammasome pathway, is one of the main drivers of inflammation^8^. The NOD-, LRR- and pyrin domain-containing protein 3 (NLRP3) inflammasome is a canonical member and can be induced and activated by a wide range of stimuli, such as lipopolysaccharide (LPS), ATP and crystal structures^9,10^. LPS primarily increases the transcription of NLRP3 and pro-IL-1β, and ATP or crystal structures further activates the assembled NLRP3 complex. Upon activation, NLRP3 recruits and cleaves procaspase1, which further activates IL-1β^11^. Thus, blocking the inflammasome or IL-1β may be a relevant strategy to suppress inflammation in chronic inflammatory diseases, including UC^12^. Interestingly, many of the other pro-inflammatory cytokines, such as IL-2, IL-6, IFN-γ, but not IL-1β, use the Janus kinase (JAK) tyrosine kinases and signal transducer and activator of transcription (STAT)-associated pathways to exert their biological effects^13^. Protein tyrosine phosphatase non-receptor type-2 (PTPN2) is a negative regulator of the JAK/STAT pathway. It dephosphorylates both JAKs and STATs, thereby inactivating the signaling strength and duration. The loss of function of PTPN2 results in the hyperactivation of the JAK/STAT pathway, and has been reported to be associated with a high risk for IBD^14^. Thus, inhibiting the JAK/STAT pathway provides a promising strategy for the treatment of IBD.

Indeed, small-molecule JAK inhibitors (e.g., Tofacitinib, Filgotinib and Upadacitinib) have been shown to be efficacious in treating UC^13,15^. Four different JAK kinases (JAK1, JAK2, JAK3 and tyrosine kinase (TYK) 2) are currently known^16^. The pan JAK inhibitor, Tofacitinib, results in predominant inhibition of JAK1/JAK3 at an adequate dosage^17,18^, leading to inhibition of JAK-associated intracellular signaling^19,20^. Tofacitinib was first developed and approved throughout Europe in 2017 as a synthetic disease-modifying anti-rheumatic drug for the treatment of moderate to severe rheumatoid arthritis (RA)^21–23^, and was subsequently also approved for the treatment of UC^24^. Over the following years, the JAK1-preferential inhibitors, Filgotinib and Upadacitinib were subsequently approved for UC^25,26^. Recent network analyses indicate that JAK inhibitors are very efficacious in individuals with IBD, especially with UC^27^. However, it is still not clear which specific molecules and pathways primarily contribute to their efficacy. Here, we studied three JAK inhibitors, Tofacitinib, Filgotinib, and Upadacitinib. We found that all three JAK inhibitors ameliorated colitis in mice with a hyperactivated JAK/STAT pathway resulting from *PTPN2*-deletion in macrophages. Moreover, we discovered an additional mode of action of JAK1 inhibitors, namely targeting the NLRP3-IL1β axis. This dual action of targeting both JAK/STAT and inflammasome pathways offers an explanation for their superior efficacy in UC treatment.

## Results

### Upadacitinib and Filgotinib are more potent in suppressing colitis in mice when compared to Tofacitinib

**Fig. 1:**
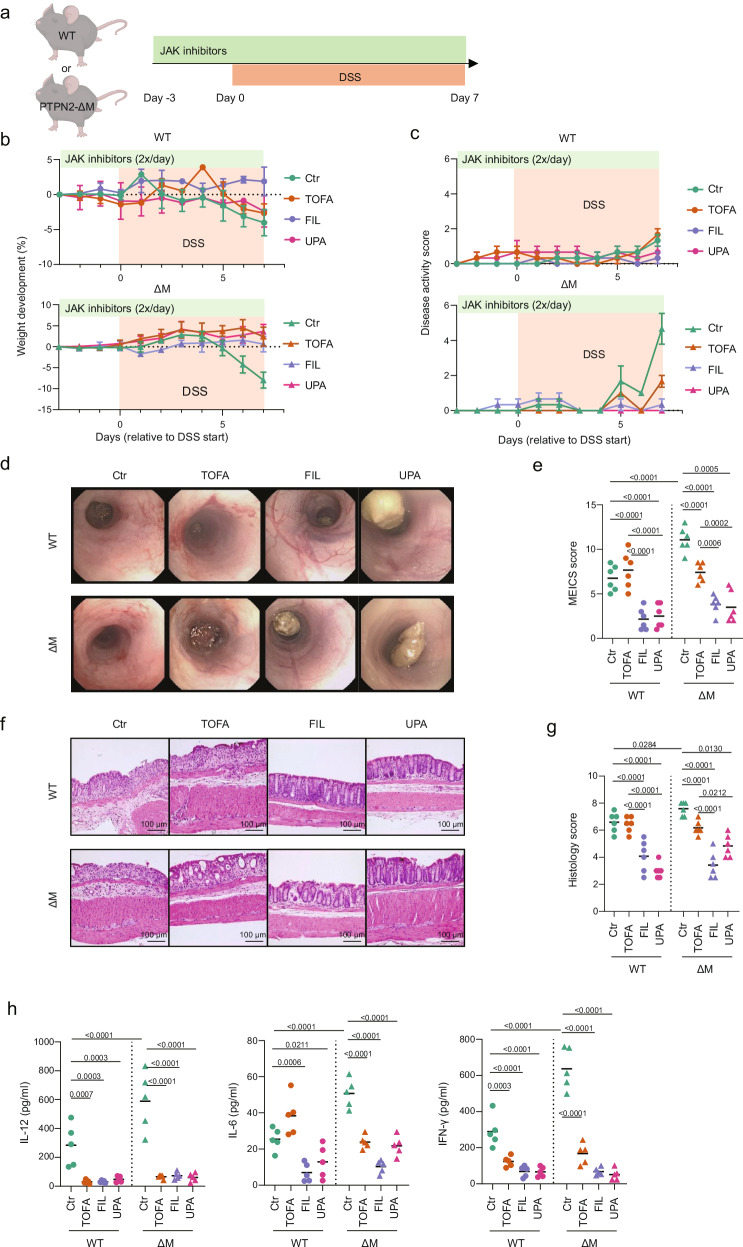
Upadacitinib and Filgotinib reduce colitis severity. PTPN2-LysMCre (PTPN2-ΔM) mice and their WT littermates were orally gavaged with the JAK inhibitors Tofacitinib (*n* = 6), Filgotinib(*n* = 6), or Upadacitinib (*n* = 6) starting 3 days prior to administration of 2% DSS. DSS was administrated in the drinking water for 7 days. **a** Experimental set-up (Created in BioRender. Bedke, T. (2026) https://BioRender.com/nqo42li); **b** weight development; **c** disease activity score over time; **d** representative images of endoscopy; **e** respective scoring of mouse endoscopy on the last day of the experiment; **f** representative images and **g** scoring for disease severity of H&E-stained sections of the terminal colon; **h** levels of the indicated cytokines in the supernatant. Colon pieces were obtained on the last day of the experiment and incubated for 24 h in cell culture medium. Every dot corresponds to one mouse. Bars represent mean value; error bars represent standard deviation (SD). Significance was assessed using two-tailed two-way ANOVA with Tukey’s multiple comparisons test. Source data are provided in the Source Data file.

To assess the efficacy of JAK inhibitors, namely Tofacitinib, Upadacitinib, and Filgotinib in colitis, we induced intestinal inflammation in wild-type (WT) mice via 2% DSS in the drinking water. Tofacitinib, Filgotinib, and Upadacitinib were administered twice daily, starting 3 days prior to the DSS application (Fig. 1a). In WT mice, Upadacitinib and Filgotinib, but not Tofacitinib treatment were protective in DSS colitis, as evidenced by reduced weight loss (Fig. 1b), lower disease activity (Fig. 1c), as well as reduced endoscopic and histologic scores (Fig. 1d–g). Similar effects were observed in a T cell transfer colitis model, where *Rag1*^*-/-*^ mice were injected with naïve T cells (Supplementary Fig. 1a). Mice that received vehicle control started showing signs of colitis around 3 weeks after injection of naïve T cells (Supplementary Fig. 1b, c), which was reduced upon treatment with Filgotinib and Upadacitinib, while only minor effects were observed upon treatment with Tofacitinib (Supplementary Fig. 1b–f). Next, we aimed to compare different dosages of Tofacitinib, Filgotinib and Upadacitinib in the DSS colitis model. We found that even higher doses of Tofacitinib were not efficient in reducing colitis in WT mice (Supplementary Fig. 2), while 50 mg/kg body weight was the most efficient dose for Filgotinib and Upadacitinib (Supplementary Fig. 3). Of note, though treatment with 50 mg/kg body weight of Tofacitinib had no effect in WT mice that were treated with DSS, this dose was effective in reducing DSS-induced colitis in mice lacking PTPN2 in macrophages (PTPN2-ΔM mice, Fig. 1), which is in line with our previous findings^28^. PTPN2 dephosphorylates many cellular substrates, including JAK/STAT molecules, and PTPN2-ΔM mice have more pro-inflammatory M1-like macrophages and show elevated expression of inflammatory cytokines, e.g., IL-6, which can be partially corrected by Tofacitinib^28,29^. In concordance with our previous findings^28,30^, PTPN2-ΔM mice suffered from a more severe colitis than their WT littermate counterparts (Fig. 1b–g). Treatment with Tofacitinib reduced the colitis severity in PTPN2-ΔM mice to the level of untreated WT mice, but we did not observe a protection beyond this. Upadacitinib and Filgotinib treatments were more efficient than Tofacitinib in both WT and PTPN2-ΔM mice (Fig. 1), indicating additional protective effects of Upadacitinib and Filgotinib that might be linked to molecular mechanisms that are not controlled by PTPN2. To identify potential mechanisms mediating the more potent effect of Upadacitinib and Filgotinib compared to Tofacitinib, colon sections from JAK inhibitor and DSS-treated mice were obtained and cultured for 24 h in cell culture medium, and effects on cytokine production were assessed. As demonstrated previously^28,30^, when compared to WT mice, PTPN2-ΔM mice showed elevated levels of IL-12, IL-6, and IFN-γ (Fig. 1h). Tofacitinib was able to reduce IL-12 and IFN-γ secretion in WT and PTPN2-ΔM mice, whereas it only reduced IL-6 in PTPN2-ΔM mice, but not in WT mice (Fig. 1h), indicating that the effect of Tofacitinib might be dependent on the inhibition of exaggerated IL-6 production. Likewise, Upadacitinib and Filgotinib were able to reduce IL-12, IL-6 and IFN-γ in both WT and PTPN2-ΔM mice (Fig. 1h). Although Tofacitinib reduced IL-6 as efficiently as JAK1 inhibitors in PTPN2-ΔM, Tofacitinib-treated mice recovered to a lesser extent than Upadacitinib- and Filgotinib-treated mice (Fig. 1d–g), suggesting there might be other molecules mediating the discrepant efficacy of different JAK inhibitors.

Taken together, these results indicate that the three JAK inhibitors exert distinct effects on colitis severity, which is also associated with different impacts on cytokine production in the inflamed mouse intestine. While Tofacitinib is able to reduce colitis severity in PTPN2-ΔM but not WT mice, Upadacitinib and Filgotinib can reduce the disease severity in both genotypes.

### Filgotinib and Upadacitinib downregulate *Nlrp3* and *Il1b* expression

**Fig. 2:**
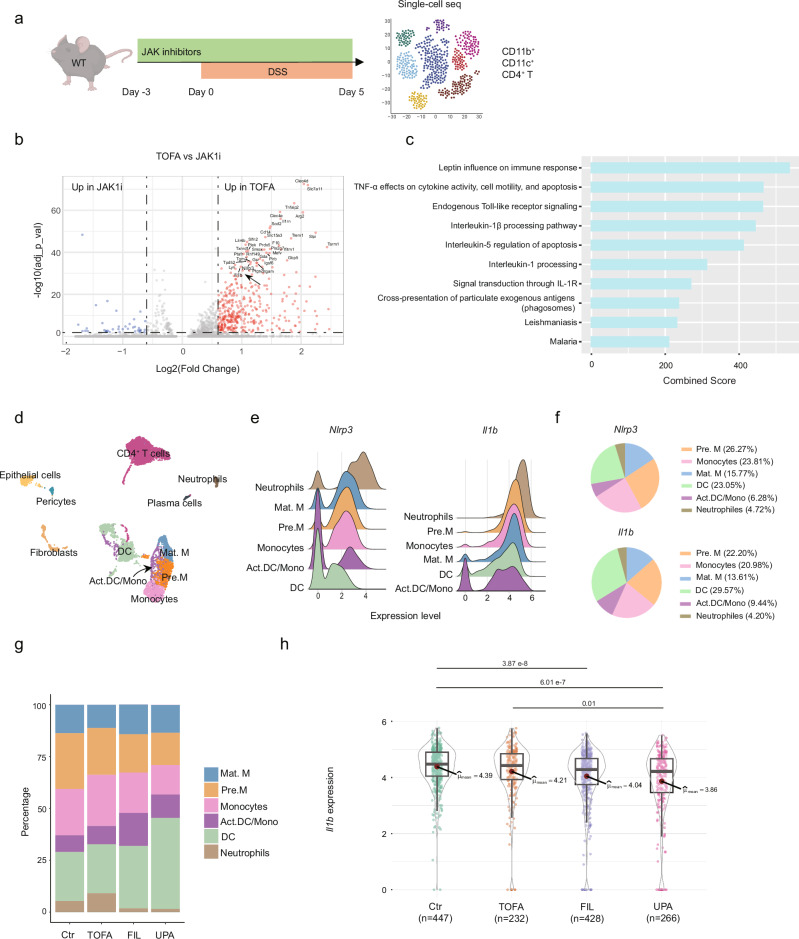
Filgotinib and Upadacitinib downregulate *Nlrp3* and *Il1b* expression. Colonic immunocytes from DSS colitis after JAK inhibitor treatment were analyzed by single-cell RNA sequencing. Mice treated with JAK inhibitors and vehicle control were sacrificed on day 5 of DSS treatment. Cells of four mice were pooled per group. CD4^+^T cells and CD11b^+^ and/or CD11c^+^ cells were isolated and FACS-sorted from the colon, and merged at 1:1 ratio. **a** Experimental set-up (Created in BioRender. Bedke, T. (2026) https://BioRender.com/nqo42li); **b** differentially expressed genes (DEGs) between the Tofacitinib-treated group and JAK1i group (including Upadacitinib and Filgotinib), significance was assessed by Wilcoxon Rank Sum test, two-tailed, with the *p*-value adjusted by Benjamini–Hochberg false discovery rate (FDR) method for multiple comparisons; **c** pathway analysis based on the upregulated DEGs in Tofacitinib versus JAK1i group; **d** UMAP of identified cell clusters (Act. DC/Mono: activated dendritic cells/Monocytes, Pre. M: premature macrophages, Mat. M: mature macrophages); **e** *Il1b* and *Nlrp3* expression among cell clusters; **f** proportions of cell clusters within *Nlrp3* and *Il1b* expressing cells; **g** proportions of myeloid cell clusters in control, Tofacitinib-, Upadacitinib- and Filgotinib- treated mice; **h** average expression of *Il1b* in macrophages, including Pre. M and Mat. M. The Center line, lower and upper hinges correspond to the median, 25th and 75th percentiles. The upper whisker extends from the hinge to the largest value no further than 1.5 × IQR from the hinge (where IQR is the inter-quartile range, or distance between the first and third quartiles). The lower whisker extends from the hinge to the smallest value at most 1.5 * IQR of the hinge. *P*-value was adjusted by Holm-Bonferroni method. Source data are provided (see “data availability”).

To further decipher the effect of the different JAK inhibitors in WT mice in DSS colitis, we performed single-cell RNA sequencing of myeloid cells (CD11b^+^ and/or CD11c^+^) and CD4^+^ T cells (with a 1:1 ratio combination) isolated and enriched from the colon of JAK inhibitor-treated WT mice upon induction of DSS colitis (Fig. 2a). To avoid the bias of different disease severities, mice were sacrificed on day 5 when they exhibited a similar disease activity (Fig. 1b, c). In total, we generated 16,111 high-quality single-cell transcriptomes. Data from all groups were integrated and depicted in a Uniform Manifold Approximation and Projection (UMAP) (Supplementary Fig. 4a). First, we compared the differentially expressed genes (DEGs) between the two JAK1 inhibitors, Upadacitinib and Filgotinib, and found that they behaved very similarly in DSS colitis, as only a few DEGs were observed between these two groups (Supplementary Fig. 4b). We then combined the cells from Upadacitinib and Filgotinib treated samples (hereafter referred to as JAK1i) and analyzed the DEGs between JAK1i and Tofacitinib. Several genes related to pro-inflammatory pathways were significantly upregulated in the Tofacitinib group, e.g., *Ifitm1/2/3* and *Irf4*, which are involved in interferon pathways^31–33^ (Fig. 2b). Of note, we also found higher expression of *Nlrp3* and *Il1b*, which are crucial components of the inflammasome pathway, in the group treated with Tofacitinib compared to JAK1i (Fig. 2b). Previous findings reported a reduction of the protein level of JAK/STAT-mediating cytokines (Fig. 1h), i.e., IL-12, IL-6 and IFN-γ. In line with this, we also observed a reduction on RNA level among the different treatments (Supplementary Fig. 4c), however the changes were minor. We next looked for pathways enriched in the upregulated DEGs in Tofacitinib versus JAK1i. We found that signaling cascades downstream of IL-1 receptor (IL-1R) and IL-1 processing signaling, especially IL-1β processing pathways were highly expressed in Tofacitinib-treated groups, compared to those in JAK1i treated groups (Fig. 2c). This suggests that JAK1i can control inflammasome signaling, while Tofacitinib cannot. Next, we identified and annotated immune cells according to the feature markers in a knowledge-based method (Fig. 2d and Supplementary Fig. 4d). By analyzing *Nlrp3* and *Il1b* expression among all cell-clusters, we found that these two genes are abundant in variable populations, in which myeloid cells are the most dominant cell type, particularly neutrophils and macrophages (Fig. 2e, Supplementary Fig. 4e). Of note, the average expression of *Il1b* in myeloid cells was decreased in JAK1i-treated mice compared to the control and Tofacitinib groups, but remained unchanged in the Tofacitinib group compared to the control group (Supplementary Fig. 4f). Among myeloid populations, macrophages, including mature macrophages (Mat. M, defined by the expression of *Itgam, Cd14, Fcgr1, Cd68* and *Cx3cr1*) and premature macrophages (Pre. M, defined by *Itgam, Cd14, Fcgr1, Cd68* and *Cx3cr1*^low^), are the major contributors of *Nlrp3* and *Il1b* expression (Fig. 2f). Notably, not only was the proportion of macrophages decreased in JAK1i groups compared to the control group (Fig. 2g), but the average expression of *Il1b* within macrophages was also reduced in the JAK1i group, which was not the case in the Tofacitinib-treated mice (Fig. 2h). This suggests again JAK1i is capable of suppressing *Il1b* expression, but not Tofacitinib.

Taken together, these data suggest that the higher efficacy of Upadacitinib and Filgotinib versus Tofacitinib in DSS colitis might result from the ability of Upadacitinib and Filgotinib to regulate *Il1b* production in myeloid cells, particularly macrophages.

### Filgotinib and Upadacitinib reduce inflammasome activation

**Fig. 3:**
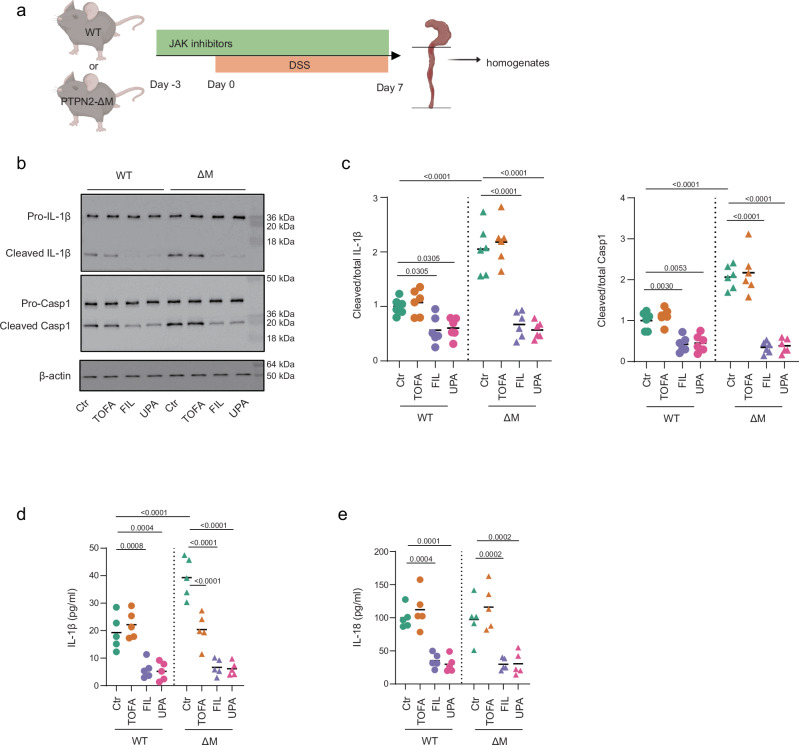
Filgotinib and Upadacitinib inhibit inflammasome activation in mouse colon. PTPN2-LysMCre (PTPN2-ΔM) mice and their WT littermates were orally gavaged with Tofacitinib, Filgotinib, Upadacitinib starting 3 days prior to administration of 2% DSS in the drinking water for 7 days. At the end of the experiment, colons were homogenized and analyzed for IL-1β and Caspase-1 cleavage by Western blot. **a** Experimental set-up (Created in BioRender. Bedke, T. (2026) https://BioRender.com/nqo42li); **b** representative pictures and **c** densitometric analysis of Western blots for the indicated proteins (*n* = 6); **d** IL-1β and **e** IL-18 levels in the supernatant of colon pieces obtained on the last day of the experiment and incubated for 24 h in cell culture medium (*n* = 5). Each data point represents one mouse. Bars represent mean value. Significance was assessed using two-tailed two-way ANOVA with Tukey’s multiple comparisons test. Source data are provided in the Source Data file.

IL-1β is activated via the inflammasome. Based on our findings from the single-cell RNA sequencing data, we subsequently assessed whether the JAK inhibitors were able to influence inflammasome activation on the protein level. To this end, we first probed colon lysates from our JAK inhibitor-treated mice for IL-1β and Caspase-1 using Western blotting (Fig. 3a). We observed reduced levels of cleaved (active) Caspase-1 and IL-1β in lysates from Upadacitinib and Filgotinib-treated mice compared to untreated controls, but no differences were found in lysates from Tofacitinib-treated mice compared to controls in both WT and PTPN2-ΔM groups (Fig. 3b, c). Likewise, we found the same trend in the production of IL-1β and IL-18 which is also a cytokine downstream of the inflammasome pathway in the supernatant of explant tissue incubated for 24 h (Fig. 3d, e).

**Fig. 4:**
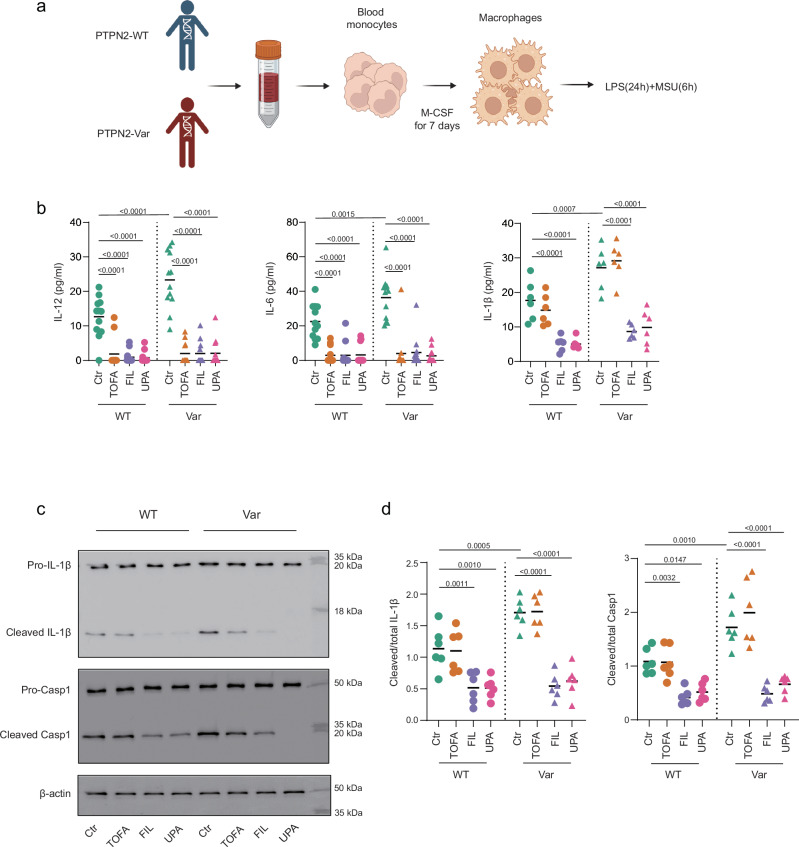
Filgotinib and Upadacitinib inhibit inflammasome activation in human cells. Monocytes were isolated from the peripheral blood of IBD patients who were either WT or variant for the loss-of-function SNP rs1893217 in the gene locus encoding PTPN2 (PTPN2-Var). These cells were differentiated into macrophages and then treated for 24 h with LPS prior to activation with MSU crystals (150 ng/ml) for 6 h. **a** Experimental set-up (Created in BioRender. Bedke, T. (2026) https://BioRender.com/nqo42li); **b** cytokine levels in the supernatant for the indicated cytokines (*n* = 12 for IL-12 and IL-6, n = 6 for IL-1β); **c** representative Western blot pictures, 17 kDa for active IL-1β and 10 kDa for active Caspase-1; **d** densitometry for the indicated proteins (*n* = 6). Every dot corresponds to one individual. Bars represent mean value. Significance was assessed using two-tailed two-way ANOVA with Tukey’s multiple comparisons test. Source data are provided in the Source Data file.

We next aimed to assess whether this is a direct effect on IL-1β-producing cells. We isolated peripheral blood monocytes from IBD patients who were either WT or variant for the IBD-associated PTPN2 SNP rs1893217, and polarized them to macrophages under M-CSF. We then treated them with lipopolysaccharide (LPS) for 24 h to induce the expression of inflammasome components. Following this, the cells were treated with MSU crystals (NLRP3 activator) to further induce inflammasome activation (Fig. 4a). This treatment resulted in robust secretion of inflammatory cytokines, including IL-6, IL-12 and IL-1β. All three JAK inhibitors were able to reduce IL-12 and IL-6 levels (Fig. 4b). However, only Upadacitinib and Filgotinib, but not Tofacitinib were able to reduce IL-1β levels (Fig. 4b), mirroring the effects observed in the colon explants from JAK inhibitor-treated mice subjected to colitis induction. Notably, the effects on IL-1β secretion were independent of the PTPN2 genotype. Western blot analyses for IL-1β and Caspase-1 indicated that the reduction of IL-1β in the supernatant of Upadacitinib and Filgotinib-treated cells was indeed due to a reduction in inflammasome activation, as evidenced by reduced levels of the mature forms of those two molecules (Fig. 4c, d).

In addition to macrophages, we also tested the effect of JAK inhibitors on human monocytes, monocyte-derived dendritic cells (DCs) and T cells. We observed that all three JAK inhibitors equally suppressed the production of IL-12 and IL-6 in all populations, and IFN-γ from T cells, especially in PTPN2-variant patients (Supplementary Fig. 5a–e). Owing to the low level of inflammasome activation induced by LPS, we were unable to reliably assess the effects of Upadacitinib and Filgotinib on IL-1β production in this setting (Supplementary Fig. 5a, c). However, upon NLRP3-specific activation with MSU, both JAK1 inhibitors markedly reduced IL-1β production in dendritic cells and monocytes from IBD patients, regardless of the PTPN2 genotype (Supplementary Fig. 5b, d). In contrast, Tofacitinib only modestly suppressed IL-1β production in monocytes and had no detectable effect in dendritic cells following MSU stimulation.

Thus, Filgotinib and Upadacitinib—but not Tofacitinib—effectively inhibit IL-1β production and suppress inflammasome activation.

### JAK inhibitors lose efficacy in mice lacking inflammasome signaling

**Fig. 5:**
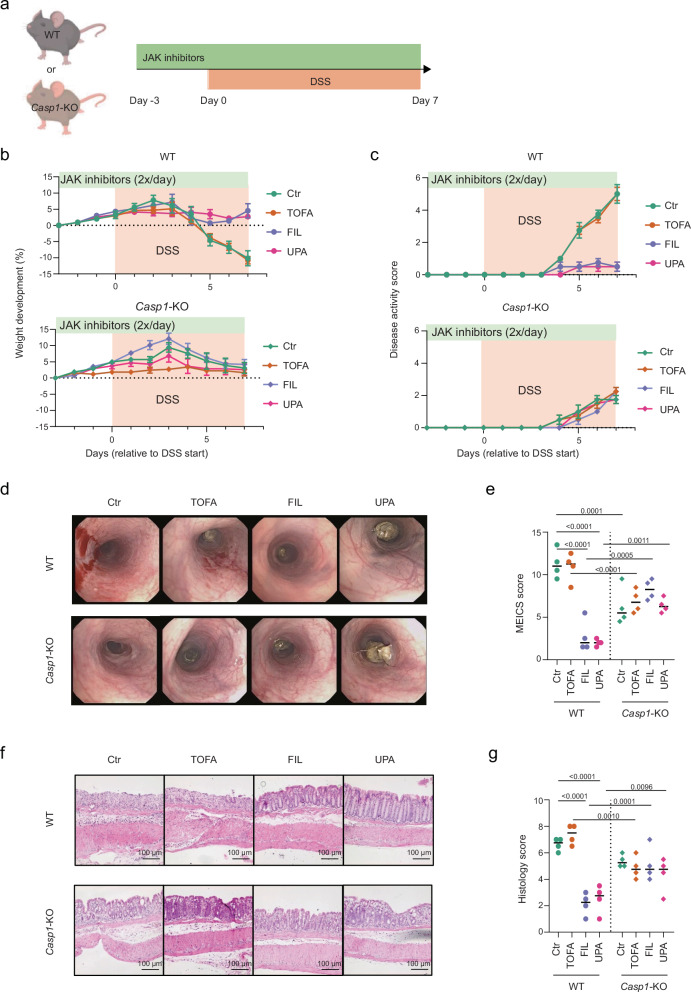
The protective effect of Upadacitinib and Filgotinib is abrogated in Caspase1-deficient mice. Caspase-1/11 knockout mice or WT controls were treated twice daily with the JAK inhibitors Tofacitinib (*n* = 4), Filgotinib (*n* = 4), and Upadacitinib (*n* = 4), starting 3 days prior to administration of 2% DSS in the drinking water for 7 days. **a** Experimental set-up (Created in BioRender. Bedke, T. (2026) https://BioRender.com/nqo42li); **b** weight development; **c** disease activity score over time; **d** representative images and **e** respective scoring of mouse endoscopy on the last day of the experiment; **f** representative images and **g** scoring for disease severity of H&E-stained sections of the terminal colon. Every dot corresponds to one mouse. Bars represent mean value; error bars represent standard deviation (SD). Significance was assessed using two-tailed two-way ANOVA with Tukey’s multiple comparisons test. Source data are provided in the Source data file. Source data are provided in the Source data file. Source data are provided in the Source Data file.

To assess whether the protective effect of Filgotinib and Upadacitinib in DSS colitis is indeed dependent on inflammasome activation, we next used Caspase1 (*Casp1*)-deficient mice (Fig. 5a). These mice are not able to assemble inflammasomes efficiently. In our hands, *Casp1*-deficient mice showed a reduced colitis severity when compared to co-housed WT mice (Fig. 5b–g), which is in line with previous findings from our group^34^. Both Upadacitinib and Filgotinib were again able to reduce colitis in WT mice, but had no effect on colitis severity in *Casp1*-deficient mice (Fig. 5b–g). To further assess whether the efficacy of JAK1 inhibitors in colitis is dependent on NLRP3, we performed DSS colitis in *Nlrp3*-deficient mice (Supplementary Fig. 6a). Consistent with the findings obtained in *Casp-1*-deficient mice, Upadacitinib showed no protective effect in *Nlrp3*-deficient mice (Supplementary Fig. 6b–d).

Thus, the effect of Upadacitinib and Filgotinib in DSS colitis is, at least in part, dependent on the inflammasome inhibition.

### Filgotinib and Upadacitinib suppress IL-1β expression via NLRP3 inflammasome

**Fig. 6:**
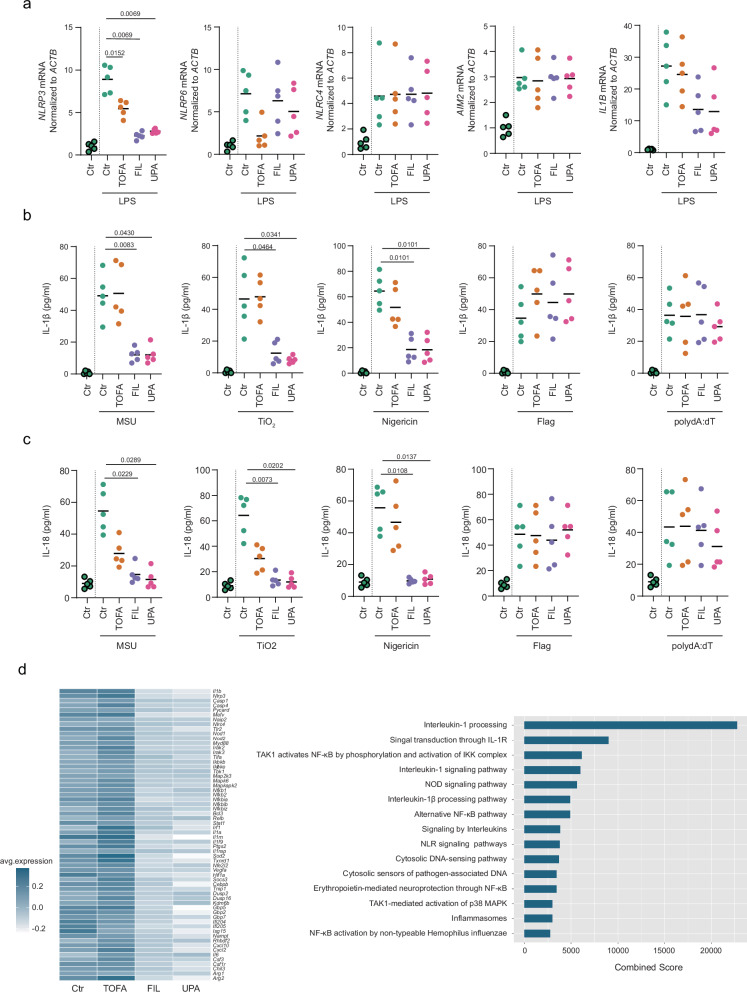
Filgotinib and Upadacitinib specifically inhibit the NLRP3 inflammasome. Monocytes were isolated from the peripheral blood of healthy volunteers (*n* = 5) and differentiated into macrophages for 7 days. **a** Macrophages were treated for 24 h with LPS and mRNA expression levels of the indicated inflammasome receptors were assessed; **b** + **c** Macrophages were treated for 24 h with LPS and then activated with MSU (150 ng/ml, 6 h), TiO2 (150 ng/ml, 12 h), Nigericin (200 ng/ml, 6 h), Flagellin (Flag, 500 ng/ml, 2 h) or poly(dA:dT) (1ug/ml, 2 h). Cell culture supernatant was assessed for IL-1β (b) and IL-18 (c) by ELISA; **d** Left: Heatmap of genes associated with NLRP3 and IL-1β-processing. These genes were significantly downregulated by JAK1 inhibitors during DSS colitis compared to Tofacitinib-treated mice and the control. Right: Bar plot of the enriched pathways of the identified genes. Bars represent mean value. Significance was assessed using two-tailed one-way ANOVA with Holm-Sidak’s multiple comparisons test. Source data are provided in the Source Data file.

To elucidate the mechanisms by which Upadacitinib and Filgotinib affect IL-1β production and different inflammasomes, we used macrophages from healthy volunteers and treated them with LPS for 24 h prior to activating different inflammasome receptors using MSU crystals (NLRP3 activator), TiO2 (NLRP3 activator), Nigericin (NLRP3 activator), Flagellin (NLRC4 activator), or double stranded DNA (dsDNA, AIM2 activator). Assessment of mRNA levels of the inflammasome receptor molecules upon treatment with LPS for 24 h revealed that Upadacitinib and Filgotinib reduced mRNA levels of *NLRP3*, and by trend, *IL1B*. However, no differences were observed in *NLRP6*, *NLRC4* or *AIM2* mRNA levels (Fig. 6a). Furthermore, IL-1β and IL-18 secretion were reduced by Upadacitinib and Filgotinib upon treatment with the NLRP3 activators MSU, TiO2, and Nigericin, while IL-1β levels upon activation of NLRC4 and AIM2 were not altered (Fig. 6b, c). These data suggest that NLRP3 is the primary inflammasome targeted by JAK1 inhibitors.

Next, we assessed the impact of Upadacitinib and Filgotinib on inflammasome activation compared to an NLRP3 inhibitor, namely MCC950. Indeed, the effect of Upadacitinib and Filgotinib was similar to that observed when treating the cells with the NLRP3 inhibitor MCC950 (Supplementary Fig. 7a, b). Of note, Upadacitinib and Filgotinib were not able to further decrease NLRP3-mediated IL-1β secretion when the cells were simultaneously treated with MCC950 (Supplementary Fig. 7a). However, it remained unclear, whether JAK1 inhibitors would bind directly to NLRP3 or if they regulate NLRP3 expression through indirect mechanisms. To address this, we assessed the ability of JAK1 inhibitors to bind NLRP3 using the NLRP3 NanoBRET™ Target Engagement assay^35^ with MCC950 as a positive control. As expected, JAK1 inhibitors reduced IL-1β production at the tested concentrations (Supplementary Fig. 8a). However, the assay revealed no detectable direct binding of any JAK inhibitors to NLRP3 (Supplementary Fig. 8b), indicating that JAK1 inhibitors modulate NLRP3 activity through indirect mechanisms.

A previous study suggested that Ruxolitinib, a JAK1/2 inhibitor could suppress NLRP3 expression indirectly via STAT3^36^. To test whether this is also the case for Upadacitinib and Filgotinib, we assessed phosphorylated STAT1 and STAT3 in lysates from DSS-induced colitis and human macrophages treated with the JAK inhibitors. The results revealed that Tofacitinib, Upadacitinib and Filgotinib reduced the phosphorylation to a similar level (Supplementary Fig. 8c, d). Given that Tofacitinib was incapable of reducing IL-1β production, it indicates that the suppression of IL-1β by JAK1 inhibitors is unlikely to be mediated by STAT1 and STAT3.

Next, we examined whether JAK1 signaling regulated the reduction of inflammasome and IL-1β expression by using siRNA to knockdown *JAK1* or *NLRP3* in human macrophages. We observed that in the absence of JAK1, Upadacitinib and Filgotinib could still downregulate the secretion of IL-1β, whereas knocking down *NLRP3* markedly compromised their inhibitory capacity on IL-1β (Supplementary Fig. 8e–g). This indicates that JAK1 inhibitors target NLRP3 independently of canonical JAK1 signaling. Of note, Tofacitinib did not show any inhibitory capacity in any of the conditions.

In order to assess potential mechanisms, we analyzed the expression of genes associated with *Nlrp3* and *Il1b* expression, which are downregulated by JAK1 inhibitors compared to Tofacitinib and control treatments in our single-cell RNA-seq dataset (Fig. 6d). Pathway enrichment analysis revealed that many of these genes were involved in NF-κB and MAPK sigalings, both of which are known to promote the expression of *Nlrp3* and *Il1b*^37–40^.This is consistent with our findings in Fig. 6a. In addition, several other individual transcription factors previously reported to directly regulate *Nlrp3* and *Il1b* expression, such as *Hif1α*^41^ and *Cebpb*^42,43^, were also found to be downregulated by JAK1 inhibitors. This suggests that a network of molecules and pathways might contribute to the reduction of the inflammasome-IL-1β axis. However, the specific underlying molecular mechanism needs to be further investigated.

Taken together, these findings indicate that apart from targeting the JAK/STAT pathway, Upadacitinib and Filgotinib can suppress NLRP3 activation in an indirect manner, thereby reducing IL-1β levels.

### Upadacitinib downregulates *IL1B* expression in UC patients

**Fig. 7:**
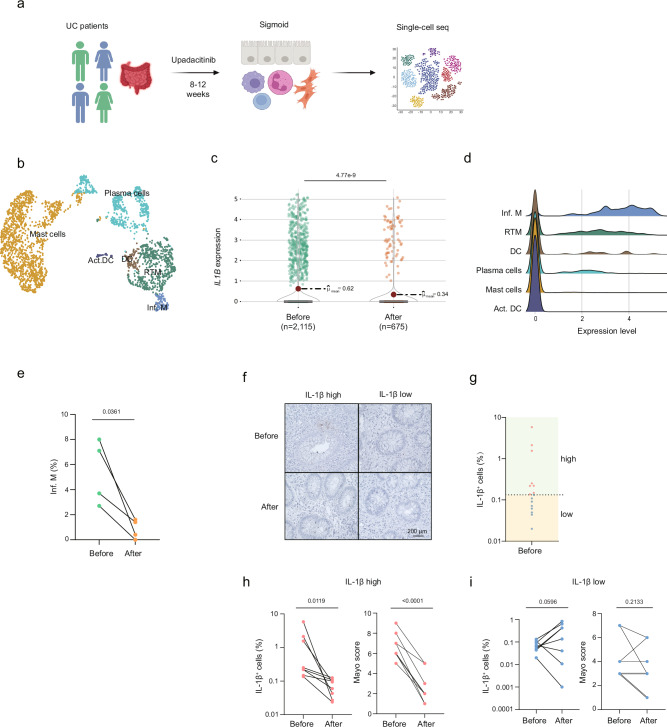
Upadacitinib downregulates IL-1β expression in UC patients. Immunocytes isolated from the lamina propria of UC patients (*n* = 4) treated with Upadacitinib were analyzed by single-cell RNA sequencing. Tissue was collected before and after 8–12 weeks of Upadacitinib treatment. **a** Experimental set-up (Created in BioRender. Bedke, T. (2026) https://BioRender.com/nqo42li); **b** UMAP of identified cell clusters (Act. DC activated dendritic cells, RTM resident tissue macrophages, Inf. M inflammatory macrophages); **c** Violin Plot of *IL1B* expression before and after Upadacitinib treatment. Significance was assessed using a two-tailed linear mixed-effects model with the patient ID as a random effect, followed by a Tukey’s post-hoc test. **d** *IL1B* expression in identified clusters; **e** proportions of inflammatory macrophages (Inf. M) in every patient before and after Upadacitinib treatment. Bars represent mean value. Significance was assessed using two-tailed paired *t* test; **f**–**i** IHC of IL-1β in sigmoid of Upadacitinib-treated UC patients (*n* = 16). **f** representative pictures showing IL-1β expression (brown). **g** quantified IL-1β expression before Upadacitinib treatment (baseline). Patients were grouped into IL-1β high or low groups based on the median of IL-1β expression among all individuals. The dotted line represents the median value. **h** quantified IL-1β expression and Mayo score of the patients in the “IL-1β high” group before and after Upadacitinib treatment. **i** quantified IL-1β expression and Mayo score of the patients in the “IL-1β low” group before and after Upadacitinib treatment. The percentage of IL-1β^+^ cells is plotted on a logarithmic scale. Bars represent mean value. Significance was assessed using two-tailed paired *t*-test. Source data are provided (see “data availability”).

To explore whether the efficacy of JAK1 inhibitors is also linked to IL-1β in human UC, we performed single-cell RNA sequencing on immune cells isolated from the lamina propria (LP) of four UC patients, before and after 8–12 weeks of Upadacitinib treatment (Fig. 7a). 26,412 high-quality single-cell transcriptomes were generated. Cells were visualized in a UMAP plot revealing 26 clusters, with clusters “14”, “25”, “12” and “22” identified as the primary producers of *IL1B* (Supplementary Fig. 9a and b). Therefore, we subclustered these populations and re-ran the UMAP. Based on the characteristic genes, we identified and annotated seven distinct populations (Fig. 7b, Supplementary Fig. 9c). Post Upadacitinib treatment, *IL1B* expression was decreased (Fig. 7c). Among all populations, macrophages, particularly the inflammatory macrophages (Inf. M), characterized by high expression of *CD14, CD68, CSF1R, TREM1, S100A12, S100A9, and S100A8* (Supplementary Fig. 9c) expressed the highest levels of *IL1B* (Fig. 7d). In a previous study, Mitsialis et al. defined a group of inflammatory macrophages possessing similar gene patterns, especially high levels of *IL1B*, and found these macrophages to be increased in active UC patients compared to healthy controls^44^. Notably, the proportion of these inflammatory macrophages was significantly reduced post Upadacitinib treatment (Fig. 7e, Supplementary Fig. 9d), which is in line with our findings in the mouse colitis model.

To assess the effect of Upadacitinib on IL-1β on the protein level, we next performed immunohistochemistry in sigmoid biopsy samples from sixteen UC patients (Supplementary Table 1), before and after Upadacitinib treatment. We noted variations of IL-1β expression among these patients at baseline (Fig. 7f, g), and thus grouped them into patients with high and low IL-1β expression based on the median value. In summary, eight patients who expressed relatively high levels of IL-1β presented a reduction in IL-1β after treatment, which correlated with the response to the therapy, as evidenced by a significantly lower Mayo score (Fig. 7h). Conversely, eight patients had low IL-1β expression at baseline, and their IL-1β expression level remained insignificantly changed post treatment, which was accompanied by a non-response to the treatment (Fig. 7i). To assess whether the observed effects are specific for Upadacitinib treatment or a general consequence linked to the reduced inflammation upon any treatment, we utilized a published single-cell RNA dataset of individuals with UC treated with anti-TNF (Adalimumab)^45^ and analyzed the *IL1B* expression (Supplementary Fig. 10a). In the myeloid population, in contrast to Upadacitinib, *IL1B* was not significantly decreased upon Adalimumab treatment (Supplementary Fig. 10b). Additionally, *IL1B* expression was not associated with the response to anti-TNF treatment (Supplementary Fig. 10c and d). Thus, it appears that the observed effect of Upadacitinib is not a general consequence of reduced inflammation. Aside from *IL1B*, we also examined the expression of other cytokines in myeloid cells, including *IL12A*, *IL6*, *IFNG*, and *IL17A*, before and after Upadacitinib treatment, and we observed minor changes in these cytokines (Supplementary Fig. 9e).

Thus, Upadacitinib can suppress IL-1β levels in individuals with UC and high IL-1β expression. This suppression is correlated with a response to the therapy. Taken together with our murine data, these results indicate that suppression of IL-1β contributes to the therapeutic efficacy of Upadacitinib in UC.

## Discussion

JAK/STAT pathways play an important role in inflammatory disease, including UC. Thus, targeting JAK activation is one of the current strategies to suppress inflammation. In our study, we explored the efficacy of different JAK inhibitors, namely Tofacitinib, Upadacitinib and Filgotinib. We found that in addition to inhibiting processes associated with the JAK/STAT pathway, Upadacitinib and Filgotinib, the JAK1 inhibitors, have the capacity to inhibit NLRP3 inflammasome activity in an indirect manner. This mode of action offers an explanation for the high efficacy of Upadacitinib and Filgotinib observed in recent clinical trials.

In this study, we found that Tofacitinib, Upadacitinib and Filgotinib have distinct effects on colitis severity, which is also associated with a different impact on cytokine production in the inflamed mouse colon. In line with a previous publication^28^, Tofacitinib was able to reduce colitis severity in PTPN2-∆M mice, but not WT mice, suggesting that Tofacitinib may have high efficacy in certain settings, e.g., in IBD patients with PTPN2 polymorphism, or hyperactivation of JAK/STAT signaling. In contrast, we found that Upadacitinib and Filgotinib could reduce the colitis severity in WT mice. This is also in line with recent network analysis suggesting that Upadacitinib is also more potent compared to Tofacitinib in patients with UC^27^. Considering these drugs are used in UC treatment at different dosages (10 mg twice a day, 45 mg once daily and 200 mg once daily for Tofacitinib, Upadacitinib and Filgotinib, respectively), we next conducted a drug titration experiment to determine whether the difference in efficacy between Tofacitinib and JAK1 inhibitors is dependent on the dosage. We observed that even in the highest concentration (450 mg/kg), Tofacitinib was not as efficacious as JAK1 inhibitors in suppressing colitis susceptibility in WT mice, indicating that the dosage is less critical in this setting. To further decipher the mechanism mediating the effect of the different JAK inhibitors in WT mice, we performed single-cell RNA sequencing of colonic immune cells. Of note, signaling cascades downstream of IL-1 receptor (IL-1R) and IL-1 processing signaling were expressed at higher levels in Tofacitinib-treated mice, compared to Upadacitinib- and Filgotinib-treated mice. In line with this, macrophages, which are the primary producers of *Nlrp3* and *Il1b* were found to be reduced in those mice treated with Upadacitinib and Filgotinib. On this basis, we hypothesized that IL-1β could be linked to the higher efficacy of Upadacitinib and Filgotinib compared to Tofacitinib. Of note, the inflammasome plays a key role in the activation of IL-1β. Inflammasomes are intracellular protein complexes and are able to sense the pathogen-associated molecular patterns (PAMPs) and damage-associated molecular patterns (DAMPs). Activated inflammasomes trigger the autocleavage and activation of pro-caspase 1, which further leads to the maturation and release of IL-1β^11^. In line with the hypothesis that Upadacitinib and Filgotinib interfere with inflammasome activation, we found reduced levels of cleaved (active) Caspase1 and IL-1β in lysates from Upadacitinib- and Filgotinib-treated mice compared to untreated control mice using western blot, but there were no significant differences in lysates from Tofacitinib-treated mice compared to control. We observed a decreased IL-1β expression by Tofacitinib in PTPN2-deficient mice using ELISA (Fig. 3d). This might be due to technical sensitivity compared to western blot. However, the reduction of IL-1β was in any case much lower compared to Upadacitinib and Filgotinib. Likewise, Upadacitinib and Filgotinib, but not Tofacitinib were able to reduce active IL-1β levels in in vitro stimulated cells upon inflammasome activation.

Yang et al.^46^ previously investigated the effect of Tofacitinib in a RA model where Tofacitinib was given to mice with RA or to BMDM culture at the dosage of 120 ng/day, or at the concentration of 1.5, or 25 μM respectively. They observed that Tofacitinib protected mice from RA, and inhibited NLRP3 and IL-1β in vivo and in vitro in macrophages, which is seemingly contradictory to our results. However, this study did not investigate the mechanism of how Tofacitinib inhibited NLRP3. In our study, we tested the efficacy of Tofacitinib in colitis models and here Tofacitinib did not have a protective effect on colitis regardless of the dosages used, which is also in line with the previous study^28,47^. Differences in the disease model used may account for the observed effects of Tofacitinib. In addition, we observed that Tofacitinib reduced the RNA levels of *NLRP3* in vitro to some extent. However, when assessing IL-1β production, this had only a minor effect. In contrast to Tofacitinib, JAK1 inhibitors displayed a more pronounced suppressive effect on IL-1β. Additionally, Tofacitinib showed an effect on IL-18 release, although it was not statistically significant. However, this effect was again noticeably less pronounced than the impact of Upadacitinib and Filgotinib. Of note, though we did not observe a direct effect of Tofacitinib on NLRP3 activation, we cannot rule out indirect effects at different doses similar to what has been described for other JAK inhibitors^36,48^.

Notably, in the mouse colitis model, we also observed a reduction in several other pro-inflammatory cytokines—such as IL-12, and IFN-γ. However, both Tofacitinib and the JAK1 inhibitors reduced these cytokines to comparable levels, suggesting that they are unlikely to be the primary drivers of the different therapeutic efficacy observed. This interpretation is further supported by our single-cell analysis, which did not implicate these cytokines as key factors underlying the distinct responses to the treatments. Collectively, our findings indicate that inhibition of the NLRP3–IL-1β axis plays a critical role in mediating the superior efficacy of Upadacitinib and Filgotinib compared to Tofacitinib in the context of UC. Nonetheless, our data do not exclude the possibility that other cytokines, including IL-6, IL-12, and IFN-γ, as well as other pathways (Fig. 2c) may also contribute. Further studies will be necessary to elucidate the specific functional roles of these cytokines and pathways in modulating treatment responses.

Engineered as a selective JAK1 inhibitor, Upadacitinib shows higher efficacy and lower adverse events associated with JAK2 and JAK3 inhibition, when compared to other JAK inhibitors^26,49–51^. To date, however, no studies have assessed whether Upadacitinib can modulate the inflammasome pathway, and whether this contributes to its improved anti-inflammatory potential. The protein NLRP3 is part of the inflammasome, and it is known that the NLRP3-Caspase1-IL-1β axis plays a prominent role in colitis pathogenesis. Specifically, enhanced IL-1β expression implies increased disease severity^52,53^. The colonic inflammation level is significantly reduced in NLRP3 knockout (KO) mice compared to WT littermate controls. Likewise, the NLRP3-R779C variant is associated with the development of very-early-onset IBD in children younger than 6 years old^54^. A similar gain-of-function mutation of NLPR3 p.D305N maintains NLRP3 in the active conformation associated with autoinflammation in adult patients^55^. To assess whether the protective effect of Upadacitinib in DSS colitis is indeed dependent on inflammasome activation, we performed DSS colitis in *Casp1* and *Nlrp3*-deficient mice. The efficacy of Upadacitinib was significantly compromised in KO mice from both genotypes compared to wild-type controls, highlighting the essential contribution of the NLRP3 inflammasome pathway in Upadacitinib therapeutic efficiency. Notably, we also observed a small and equal protective effect from all three drugs based on histology scores (Fig. 5g) by trend, but not based on endoscopy (Fig. 5e) in *Casp1*-deficient mice. This supports the notion that these drugs also exert their effects through the suppression of the JAK/STAT pathway. However, as for Upadacitinib and Filgotinib the effect was much lower in *Casp1*-deficient mice when compared to wild-type mice, suggesting that the inhibition of the inflammasome in conjunction with JAK/STAT pathways contributed to the advanced efficacy of JAK1 inhibitors.

Additionally, to test the effect of Upadacitinib on inflammasome activation, we also performed an in vitro assay using macrophages from healthy volunteers and treated them with LPS for 24 h prior to activation with MSU crystals, TiO2, Nigericin, Flagellin, or double stranded DNA (dsDNA) to activate different inflammasome receptors. We found that Upadacitinib and Filgotinib could only reduce the IL-1β production induced by NLRP3 activators. In addition, we compared the impact of Upadacitinib and Filgotinib on inflammasome activation to the LPS control and an NLRP3 inhibitor, namely MCC950. Several inhibitors that target the NLRP3 inflammasome have been reported^56–59^. Among them, MCC950 (also called CP-456,773 or CRID3) is the best characterized and the most potent NLRP3 inhibitor, which blocks NLRP3 activation at nanomolar concentrations^59^. Of note, it has been reported that MCC950 ameliorates inflammation in a murine model of colitis^59–61^, which is why we decided to use this compound as a control. Indeed, the effect of Upadacitinib and Filgotinib was similar to that observed when treating the cells with the NLRP3 inhibitor MCC950. Therefore, we assessed whether JAK1 inhibitors are able to interact with NLRP3 directly similar to MCC950 using a NLRP3 binding assay. Results revealed no binding signals between JAK1 inhibitors and NLRP3, indicating an indirect regulation.

In previous studies, Patel et al. ^48^ reported that Baricitinib, a JAK1/2 inhibitor, suppresses the NLRP3–caspase-1 axis, although the underlying mechanism remained unclear. Zhu et al.^36^ further demonstrated that Ruxolitinib, another JAK1/2 inhibitor, can indirectly inhibit NLRP3 activation by suppressing STAT3 in a murine cerebral ischemia model. Based on these findings, we examined STAT1 and STAT3 phosphorylation in lysates from DSS-induced colitis in mice and in human monocyte-derived macrophages treated with Tofacitinib, Upadacitinib, or Filgotinib. However, we did not detect any differences among the three inhibitors (Supplementary Fig. 8c, d). Given that Tofacitinib failed to downregulate inflammasome signaling, these data collectively suggest that STAT1 and STAT3 are unlikely to mediate the JAK1 inhibitor–induced reduction of IL-1β in this context.

We next examined whether the JAK1 pathway regulates the NLRP3-IL-1β axis by knocking down JAK1 or NLRP3 in human macrophages. Notably, JAK1 inhibitors continued to efficiently reduce IL-1β levels in *JAK1*-deficient macrophages, but not in *NLRP3*-deficient cells. These results indicate that JAK1 inhibitors suppress IL-1β independently of canonical JAK1 signaling.

To obtain a broader overview of transcriptional changes associated with IL-1β regulation, we analyzed genes involved in NLRP3 inflammasome activation and IL-1β processing that were downregulated by JAK1 inhibitors compared with Tofacitinib or control treatments in our mouse single-cell RNA-seq dataset. Pathway enrichment analysis revealed that many of the genes were involved in NF-κB and MAPK cascades. NF-κB indeed plays an important role in promoting the transcription of both NLRP3 and IL-1β^37,38^. MAPK cascades are activated in parallel with NF-κB, and co-regulate the expression of NLRP3 and IL-1β^39,40^. Consistent with this, we did observe that JAK1 inhibitors reduced both *NLRP3* and *IL1B* transcript levels in macrophages (Fig. 6a), suggesting that interference with NF-κB and/or MAPK signaling may contribute to their mechanism of action. Moreover, several additional genes previously reported to regulate NLRP3 and IL-1β expression, such as *Hif1a*^41^ and *Cebpb*^42,43^, were also downregulated following JAK1 inhibition, which may also contribute to suppression of the inflammasome pathway.

Collectively, our data indicate that JAK1 inhibitors can modulate the inflammasome pathway; however, it appears that many molecules and pathways might participate in the modulation, and the precise mechanisms appear to differ between compounds. Therefore, further studies are warranted to elucidate the detailed molecular pathways through which JAK1 inhibitors regulate the NLRP3-IL-1β axis.

To determine whether our findings can be translated to human biology, we performed RNA sc-seq on immune cells isolated from the LP of patients with UC before and after 8–12 weeks of Upadacitinib treatment. When comparing gene expression in myeloid subsets, which are the primary source of *IL1B* expression upon Upadacitinib therapy, we found a lower expression of *IL1B* post treatment. We confirmed these data on the protein level from UC patients: individuals with a high level of IL-1β showed a reduction of IL-1β after treatment, which correlated with the response to the therapy, as evidenced by significantly lower Mayo scores. Conversely, individuals with low IL-1β expression before treatment exhibited unchanged IL-1β expression post treatment, which was accompanied by inferior response to the treatment. Of note, patients with high IL-1β expression and therapeutic response to Upadacitinib were not simultaneously treated with any other advanced IBD treatments. But notably, two of the eight patients with low pre-treatment IL-1β expression had received Budesonide foam or Prednisone. Steroids are known to suppress inflammatory responses, including IL-1β. Therefore, larger multicenter studies that also include other JAK inhibitors are warranted to provide greater generalizability and comparable efficacy data in patients.

Interestingly, our results demonstrate a robust suppression of IL-1β at a concentration of approximately 5 μM Upadacitinib in vitro. Notably, a previous study suggested that colonic concentrations of Upadacitinib may fall within a similar range^62^, and findings from a study evaluating other JAK inhibitor further support this estimate^63^. This is in line with our human data, which shows a marked reduction of IL-1β in colonic tissue from patients who respond to Upadacitinib. Nevertheless, additional studies are warranted to directly quantify colonic Upadacitinib levels and to correlate these concentrations with IL-1β levels and clinical outcomes.

Next, we aimed to test, whether this is a general outcome linked to reduced inflammation upon treatment. We re-analyzed a published longitudinal dataset of UC patients before and upon Adalimumab, an anti-TNF treatment. Despite inducing remission, Adalimumab did not decrease *IL1B* expression in the myeloid cells, and *IL1B* expression was also not associated with the response to anti-TNF therapy. Overall, our data suggest that the efficacy of Upadacitinib therapy in UC might be correlated to its capacity to inhibit IL-1β activation via the inhibition of the NLRP3 inflammasome. Therefore, Upadacitinib might work more efficiently in individuals with UC and high IL-1β expression. Thus, IL-1β may represent a promising biomarker to guide clinical therapeutic strategies. However, this hypothesis requires validation in future multicenter prospective trials with larger patient cohorts.

In case studies of Mendelian disease-like IBD with *IL10*-deficiency, the blockade of IL-1 signaling can successfully treat intestinal inflammation^64,65^. Large-scale studies of IL-1 blockade in polygenic IBD patients with acute severe UC are ongoing, however, results appear to be rather disappointing^66^. We hypothesize, therefore, that targeting IL-1β alone is insufficient to provide effective protection against colitis. Dual action, such as targeting both the JAK/STAT signaling pathway and the inflammasome axis may increase the therapeutic efficacy. This highlights the importance of simultaneously modulating these interconnected inflammatory pathways. Thus, taken together, it appears that inhibition of the NLRP3-IL-1β axis plays an important role in controlling colitis in conjunction with JAK/STAT inhibition, but may not be sufficient to control colitis alone. Further studies will be essential to clarify this point.

In conclusion, Upadacitinib and Filgotinib exhibited a higher efficacy than Tofacitinib in WT mice upon DSS-induced colitis. The superior efficacy can at least in part be attributed to their capacity to inhibit the NLRP3 inflammasome and thus IL-1β activation. In line with this, Upadacitinib was also found to reduce NLRP3 and IL-1β expression in individuals with UC, and this was associated with a response to the therapy. To conclude, we have identified a mode of action of Upadacitinib and Filgotinib, which provides evidence for the high efficiency of JAK1 inhibitors.

## Methods

### Animal and human ethics

All animal experiments were conducted according to Swiss and Hamburg animal welfare legislation and approved by the local veterinary authorities (Veterinäramt des Kantons Zürich and Behörde für Justiz und Verbraucherschutz Hamburg). All patients enrolled in this study have signed informed consent which was approved by the local ethics committees in Zürich and Hamburg (Cantonal Ethics Commission Zurich, Switzerland; Ethikkommission der Ärztekammer Hamburg, Germany).

### Mice and colitis induction

PTPN2^fl/fl^-LysMCre^+/-^ (PTPN2-ΔM) mice were previously generated in our lab^30^ and a local colony maintained by crossing PTPN2^fl/fl^ mice with PTPN2^fl/fl^-LysMCre^+/-^ mice. In experiments with PTPN2-ΔM mice, PTPN2^fl/fl^ LysMCre^-^ littermates were used as controls. Caspase-1 KO mice and non-littermate WT controls were purchased from Jackson Laboratories (strain #016621). Prior to the start of experiments, WT and Caspase-1 KO mice were co-housed for at least 3 weeks in order to eliminate differences in their microbiome. *Nlrp3*-deficient mice were kindly provided by Professor Andy Wullaert (University of Antwerp, Belgium), and *Rag1*^-/-^ mice were obtained from Jackson laboratories. All mice were kept in single-ventilated cages in specific-pathogen-free conditions with 12 h dark/light cycle, standard animal chow and water *ad libitum*, ambient temperature of 20 ± 2 °C, humidity of 55 ± 10%. For colitis models involving PTPN2-ΔM mice and their WT littermates, *Rag1*^-/-^ mice, as well as Caspase-1 KO and WT mice, females at a weight of 20–25 g were used for the experiments. In the colitis experiments with NLRP3 KO and their WT littermates, both male and female mice were included. To induce DSS-mediated colitis, the mice received 2% DSS in their drinking water for 7 days. To induce T cell transfer colitis, *Rag1*^-/-^ mice were injected with 0.25 × 10^6^ naïve T cells that were obtained from the spleen of WT donor mice using the EasySep™ Mouse CD4^+^ T Cell Isolation Kit from Stemcell Technologies (# 19852) and subsequent sorting of CD62L^high^, CD44^low^ naïve T cells on an Aria III sorter from BD. JAK inhibitors (50 mg/kg body weight, suspended in 2% methylcellulose) were administered twice daily via oral gavage, starting 3 days before the DSS application or 3 days prior to the injection of naïve T cells. Unless otherwise stated, mice were sacrificed on day 7 after the first DSS exposure.

### Patient samples

Patients (*n* = 16, 6 female and 10 male participants, 30–62 years of age) diagnosed with UC and treated with Upadacitinib were enrolled in this study. Tissue samples were collected from the sigmoid during endoscopy before and after 8–12 weeks of Upadacitinib treatment. Colitis activity was evaluated by the Mayo score (Supplementary Table 1).

### Assessment of mouse colitis severity

Disease activity (consisting of the following six parameters: (1) mouse activity level, (2) behavior, (3) signs of pain, (4) stool consistency, (5) blood in stool, (6) and weight loss) was monitored daily. On the last day, colonoscopy was performed on anesthetized animals using a mouse endoscope (IMAGE1 STM from Karl Storz, Tuttlingen, Germany). Colitis severity was assessed using the murine endoscopic index of colitis severity (MEICS) scoring system^67^ using the following five parameters: (1) transparency of the colon, (2) changes of the vascular pattern, (3) fibrin visibility, (4) granularity of the mucosal surface, and (5) stool consistency. Histological scoring for inflammatory infiltration and epithelial cell damage was performed on H&E-stained sections of the most distal 1.5 cm of the mouse colon^30,67^.

### Cytokine assessment in mouse colon

To assess cytokine production in mouse colons, colon pieces were obtained on the last day of the DSS experiment and cultured in RPMI (Life Technologies) supplemented with 10% FCS, 1% Penicillin/Streptomycin (Life Technologies). After 24 h, the supernatant was collected and cytokine levels were assessed using the Mouse 23-Plex assay from BioRad.

### In vitro assay and inflammasome activation

Blood was obtained from patients enrolled in the Swiss IBD cohort or from healthy volunteers. T cells were isolated and activated by anti-CD3/anti-CD28 dynabeads (Thermo Fisher Scientific, #10131D). Monocytes were isolated for the in vitro assay or differentiated further into DCs or macrophages in RPMI supplemented with 10% FCS and recombinant human GM-CSF (40 ng/ml) and IL-4 (40 ng/ml) or M-CSF (20 ng/ml) for 7 days^29^. The cells were collected with ice cold PBS containing 2 mM EDTA by gentle pipetting and seeded in 24 well plates for experiments. Prior to inflammasome activation, the cells were treated with ultra-pure lipopolysaccharide (LPS; 50 ng/ml, Sigma-Aldrich) for 24 h. Mono-sodium urate (MSU), TiO2, Flagellin and polydA:dT were obtained from Invivogen. Flagellin and polydA:dT were transfected into the cells using DOTAP reagent. JAK inhibitors were applied at a final concentration of 50 μM in the assay. The NLRP3 inhibitor MCC950 (Sigma-Aldrich) was used at 1 μM. For the dose-response assay, 1 nM, 10 nM, 100 nM, 1 μM, 2.5 μM, 5 μM, 10 μM and 100 μM of Upadacitinib and Filgotinib were applied in human monocyte-derived macrophages.

### NLRP3 binding assay

NLRP3 NanoBRET™ Target Engagement utilized bioluminescent resonance energy transfer (BRET) following the manufacturer’s protocol (Promega, #CS1810C523). Briefly, HEK293 cells were transfected with NLRP3-Nanoluc® fusion construct (NLRP3 RefSeq: NP_001120933.1 or UniProt: Q96P20-5; NanoLuc® Protein Fusion Flexi® Vector pFC32K, Promega, #N1341). With the addition of substrate, the Nanoluc tag from NLRP3 emitted the signal (donor) at 450 nm. The lyophilized NLRP3 tracer, binding to the NACHT domain and labeled with a chemically reactive dye, was resuspended in DMSO, and prepared at a working concentration of 20X in NanoBRET™ TE tracer dilution buffer (12.5 mM HEPES, 31.25% PEG-400, pH 7.5). NLRP3 tracer was added in the culture together with JAK inhibitors and MCC950 at concentrations of 0.000508, 0.00152, 0.00457, 0.0137, 0.0412, 0.123, 0.37, 1.11, 3.33, 10 μM. Prior to BRET measurement, NanoBRETTM Nano-Glo® Substrate was added to the cells. With the tracer binding to NLRP3, the donor light excited the tracer, emitting the signal at 600 nm (acceptor). Competition for the binding site by inhibitors displaced the tracer, thereby decreasing the acceptor signal. Measurements were performed on a GloMax® Discover (Promega, Cat#GM3000) luminometer. Occupancy of NLRP3 by inhibitors was calculated based on the detected signals and formula provided in the manufacturer’s protocol^35^.

### Western blots

1 cm colon pieces were homogenized in 350 μl M-PER (Thermo fisher Scientific, #23225) on an OctoMACS dissociator (Miltenyi) and incubated for 15 min prior to centrifugation at 13,000 × *g* for 10 min. For cell culture samples, cells were homogenized in M-PER using a 25G needle, incubated for 10 min, and centrifuged for 10 min at 13,000 × *g*. Protein concentration was estimated in the supernatant by absorption measurement at 280 nm using a NanoDrop (Thermo Fisher Scientific). Equal amounts of protein were mixed with LDS sample loading buffer (Thermo fisher scientific, #84788) and separated on polyacrylamide gels before blotting onto nitrocellulose membranes. The membranes were blocked in 1% BSA, 3 % milk in tris-buffered saline containing 0.05% Tween (TBST) for 2 h before incubation with primary antibody (anti-human IL-1β, R&D Systems; anti-mouse IL-1b, R&D/bio-Techne; anti-human/mouse Caspase-1, AdipoGen; anti-human STAT1 and STAT3, Cell Signaling Technologies; anti-mouse STAT1 and STAT3, Cell signaling Technologies) over night, washing three times in TBST, incubation with secondary antibody (horseradish peroxidase (HRP)-coupled anti-mouse; or HRP-coupled anti-rabbit antibody) for 2 h, washing three times in TBST and developing with ECL solution (Thermo Fisher Scientific, #32106) and imaging on a FusionX Western blot imager. Uncropped and unprocessed scans of the blots are included in the Source Data file.

#### JAK1 and NLRP3 knockdown by siRNA in human macrophages

Monocytes were isolated from buffy coats from healthy donors, then differentiated into macrophages in RPMI supplemented with 10% FCS and recombinant human M-CSF (40 ng/ml). On day 6, macrophages were transfected by *siJAK1* (Dharmacon, #M-003145-02-0005) or *siNLRP3* (Dharmacon, #M-017367-00-0005) for 48 h, then activated by LPS and treated with Tofacitinib, Upadacitinib, Filgotinib, MCC950 or DMSO as a control. The level of IL-1β in the supernatant was measured by ELISA.

#### Preprocessing and analysis of single-cell RNA-seq

For murine cell isolation for single-cell RNA sequencing, colon tissue was digested in dithiothreitol (DTT) buffer for 20 min at 37 °C on a shaker and was subsequently incubated in collagenase buffer for 30 min at 37 °C on a shaker. Next, immune cells were separated using a density gradient consisting of 40% and 60% Percoll. Cells from four mice belonging to the same group were pooled and frozen in freezing buffer (10% DMSO in FBS) in Mr. Frosty. After thawing, fluorescence-activated cell sorting (FACS) was performed with AriaFusion sorter (BD Bioscience) to isolate populations of CD11b^+^ and/or CD11c^+^ cells and CD4^+^ T cells, which were then combined at a 1:1 ratio. A total of 20,000 cells were loaded in a Chromium Next GEM Chip K (10x Genomics, USA). The following fluorescent antibodies were used for FACS: Fixable Viability Dye eFluorTM 506 (Amcyan, eBioscience), anti-CD45-BV785 (Biolegend), anti-CD3-BV650 (Biolegend), anti-CD4-PE-Cy7 (Biolegend), anti-CD11b-PE (Biolegend), anti-CD11c-PE (BD Biosciences), and anti-CD19-APC-Cy7 (Biolegend). Amcyan^- ^CD45^+^ CD3^- ^CD19^- ^CD11b^+^ and/or CD11c^+^ cells were sorted and combined with Amcyan^-^ CD45^+^ CD3^+^ CD4^+^ cells in the ratio stated above. For human cell isolation for single-cell RNA sequencing, cells from the lamina propria (LP) of the sigmoid colon were isolated. The tissue was digested in DTT buffer for 15 min at 37 °C on a shaker, then further cut and digested for 30 min in RPMI 1640 medium containing DNase I (2 μg/ml, Roche, Switzerland) and collagenase (1 mg/ml, Roche, Switzerland) at 37 °C on a shaker. A total of 10,000 LP cells and 20,000 FACS-enriched CD45^+^ cells (anti-human CD45 PE-Cy7, Biolegend) were counted and loaded in the Chromium Next GEM Chip K. For both murine and human cells, gene expression libraries were generated using the 10X Genomics Chromium Single-Cell 5′v2 Reagents kit according to the manufacturer’s instructions. The libraries were sequenced with an Illumina NovaSeq 6000 system. The Cell Ranger software pipeline (v5.1.0, 10X Genomics) was used to demultiplex cellular barcodes and map reads to the reference genome (command cellranger count). The feature-barcode matrices for all the samples and the subsequent analyses were processed using R packages including Seurat^68^ (v5.0.1), and EnrichR^69^ platform. In the QC step, cells with 200-2500 unique features and mitochondrial counts below 5% for murine samples, and 5–8% for human samples were retained in order to exclude the cells of low quality, doublets, and dying cells. Data were normalized using the function “NormalizeData”. Highly variable features and repeated variable features were identified using function “FindVariableFeatures”. “FindIntegrationAnchors” and “IntegrateData” for murine samples and “Harmony” (harmony package, v1.2.0) for human samples were used to integrate the data. The integrated dataset was then scaled using “ScaleData”. Principal components analysis (PCA) was performed by “RunPCA” with default settings to reduce dimensionality. “FindNeighbors” (dims = 12 for murine dataset, 20 for human total dataset, and 6 for human *IL1B*-expressing clusters (cluster 12, 14, 22, 25)) and “FindClusters” (resolution = 0.6 for murine and human datasets) were used to cluster the cells. Clusters were then visualized in UMAP (dims = 12 for murine dataset, 15 for human total dataset and 6 for* IL1B*-expressing clusters). Gene expression in clusters was visualized using DotPlot, VlnPlot, FeaturePlot (ggplot2 package, v3.5.0; ggstatsplot package, v0.12.4; patchwork package, v1.2.0; ggrepel package, v0.9.5). For the analysis of differentially expressed genes (DEGs) and pathway enrichment, “FindMarkers” was utilized to identify DEGs between Tofacitinib and JAK1 inhibitors in murine RNA-seq data. DEGs were defined by an absolute log2 fold change higher than 0.6 and an adjusted *p*-value below 0.01. DEGs were then visualized using VolcanoPlot (ggplot2). Enriched pathways were determined with the reference of the “Bioplanet2019” dataset in EnrichR. Pathways were ranked based on the combined score, which takes the log of the *p*-value from the Fischer exact test and multiplies this value by the z-score of the deviation from the expected rank, and visualized using BarPlot (ggplot2). For human samples, to assess the significance of *IL1B* expression before and after treatment, a linear mixed-effects model with the patient ID as a random effect was applied using the “lme” function (nlme package, v3.1-163), followed by a Tukey’s test post-hoc using the “glht” function (multcomp package, v1.4-29). To identify pathways enriched among genes associated with NLRP3 and IL-1β expression that were downregulated by JAK1 inhibitors compared to Tofacitinib and the control, DEGs were identified using a threshold of the absolute log2 fold change higher than 0.3 and adjusted *p-value* below 0.05. The “Bioplanet2019” pathway database was used as the reference, and the enriched pathways were visualized using BarPlot.

#### Analysis of published single-cell RNA dataset

Myeloid cell population from the single-cell RNA dataset of Thomas et al. was downloaded and analyzed. Ten UC patients were filtered and selected based on the disease type (UC), sample site (sigmoid), and paired sample collection. The average expression of *IL1B* was calculated and grouped based on the median value. Clinical characteristics are shown in the Supplementary Table 2. The comparison of *IL1B *pre- and post-Adalimumab treatment in Supplementary Fig. 9b was analyzed in four patients with remission.

#### Immunohistochemistry

Sigmoid tissues were fixed in 4% buffered formalin, dehydrated, and embedded in paraffin. Paraffin tissue sections were cut at 5 μm. Samples were dewaxed and endogenous peroxidases were inactivated (Dako Peroxidaseblock). To unmask the antigen, heated Citrate (PH  6) was used and the non-specific binding was blocked by 10% BSA in PBS. Slides were incubated with anti-human IL-1β antibody (Cat.: AF-201-SP, 1 μg/ml; R&D Systems, USA) for 1 h at room temperature followed by incubation with the Anti-Goat IgG VisUCyte HRP Polymer Antibody (Cat.: VC004, R&D Systems, USA) and DAB chromogen. Tissues were counterstained with hematoxylin. Stained samples were mounted in a mounting gel. Quantification of 4–5 sections was evaluated and averaged per patient per time point by QuPath (v0.5.1).

### Statistics and reproducibility

Unless otherwise stated, statistics were performed using GraphPad Prism 10. Statistical tests are given in the figure legends. In the animal experiments, sample size was chosen based on the previous work from our group using the same models. Based on the previous data, we conducted G-power analysis with an ANOVA design to determine the sample size. Data presented in animal studies were combined data from at least two independent experiments. No data were excluded from the analyses.

### Reporting summary

Further information on research design is available in the Nature Portfolio Reporting Summary linked to this article.

## Supplementary information

Supplementary information

Reporting Summary

Transparent Peer Review file

## Source data

Source data

## Data Availability

Source data are provided with this paper. Mouse single-cell RNA-seq raw and processed data have been deposited in the database^70^ (10.25592/uhhfdm.18257). Human RNA-seq raw data and single-cell count matrices are available under restricted access due to the need to preserve data privacy. Access can be obtained by contacting Prof. Samuel Huber (s.huber@uke.de), who aims to respond to requests within three working days and to provide the data within 1 month after a data transfer agreement has been signed by both parties. The anonymized processed human data have been deposited in the database^70^ (10.25592/uhhfdm.18257). Single-cell RNA-seq data from Thomas et al.^45^ reanalyzed in this study are publicly accessible via the original publication. Source data are provided with this paper.
