# Supplementary material for: Indirect inhibition of the NLRP3-interleukin-1β axis contributes to the efficacy of JAK1 inhibitors in experimental colitis and human ulcerative colitis
Source: Nat Commun. 2026 Apr 18;17:5429. doi: 10.1038/s41467-026-71808-y (PMC13279951; doi:10.1038/s41467-026-71808-y)
Supplement: Supplementary file 1 — Supplementary information [file 41467_2026_71808_MOESM1_ESM.pdf]

Indirect inhibition of the NLRP3-interleukin-1β axis contributes to the efficacy of JAK1 inhibitors in experimental colitis and human ulcerative colitis

Supplementary methods

Murine cells isolation for single-cell RNA sequencing. Colon tissue was digested in dithioerythritol (DTT) buffer for 20 minutes at 37℃ on a shaker and were subsequently incubated in collagenase buffer for 30 minutes at 37 ℃ on a shaker. Next, immune cells were separated using a density gradient consisting of 40% and 60% Percoll. Cells of four mice belonging to the same group were pooled together and frozen in cell-freeze buffer (10% DMSO in FBS) in Mr. Frosty.

Cells were thawed. Fluorescence activated cell sorting (FACS) was performed with AriaFusion sorter from BD to isolate populations of CD11b+ and/or CD11c+ cells and CD4+ T cells, which were all sorted individually and merged in a ratio of 1:1. A total of 20.000 cells were loaded in a Chromium Next GEM Chip K (10x Genomics, USA). The following fluorescent antibodies are used: Live/dead staining by Fixable Viability Dye eFluorTM 506 (Amcyan, eBioscience), anti-CD45-BV785 (Biolegend), anti-CD3-BV650 (Biolegend), anti-CD4-PE-Cy7 (Biolegend), anti-CD11b-PE (Biolegend), anti-CD11c-PE (BD Biosciences), anti-CD19-APC-Cy7 (Biolegend). Amcyan$^-$CD45$^+$CD3$^-$

CD19⁻CD11b⁺ and/or CD11c⁺ cells, were sorted and combined with Amcyan⁻ CD45⁺CD3⁺CD4⁺ cells in the ratio stated above.

Human cell isolation for single-cell RNA sequencing. Cells from the lamina propria (LP) of the sigmoid were isolated. The tissue was digested in DTT buffer for 15 minutes at 37℃ on a shaker, then further cut and digested for 30 min in RPMI 1640 medium containing DNase I (2ug/ml, Roche, Switzerland) and Collagenase (1mg/ml) from Clostridium histolyticum (Roche, Switzerland) at 37°C on a shaker. A total of 10,000 LP cells and 20,000 FACS sort-enriched CD45⁺ cells (anti-human CD45 PE-Cy7, Biolegend) per sample were counted and loaded respectively in the Chromium Next GEM Chip K.

Sample normalization and integration. Data were normalized by function "NomalizeData". Highly variable features and repeated variable features were identified and selected by function "FindVariableFeatures". "FindIntegrationAnchors" and "IntegrateData" for murine samples and "Harmony" (harmony package, v1.2.0) for human samples were performed to integrate the data.

Dimensionality reduction and clustering. The integrated dataset was scaled by "ScaleData". Principal components analysis (PCA) was performed by "RunPCA"(default) to reduce dimensionality. "FindNeighbors" (dims = 12 in murine, 20 in human total dataset, and 6 in human *IL1B*-expressing clusters (cluster 12, 14, 22,

25)) and “FindClusters” (resolution=0.6 in murine and human samples) were performed to cluster the cells, and visualized in UMAP (dims = 12 in mice, 15 in human total dataset and 6 in *IL1B*-expressing clusters). Populations were identified and annotated by “FindAllMarkers”. Gene expression in clusters was visualized using DotPlot, VlnPlot, FeaturePlot (ggplot2 package, v3.5.0; ggstatsplot package, v0.12.4; patchword package, v1.2.0; ggrepel package, v0.9.5).

Analysis of differential expressed genes (DEGs) and pathways. “FindMarkers” in Seurat was utilized to assess DEGs between Tofacitinib and JAK1 inhibitors in mouse RNA-seq data. Threshold of DEGs was set as the absolute value of Log2 fold change lower than 0.6 and p-value lower than 0.01. DEGs were then visualized using VolcanoPlot (ggplot2). Enriched pathways were determined with the reference of the “Bioplanet2019” dataset in EnrichR[70]. Pathways were ranked based on a calculation of combined score, which takes the log of the p-value from the Fischer exact test and multiplies this value by the z-score of the deviation from the expected rank, which was later visualized using BarPlot (ggplot2). For human samples, to test significant differences in *IL1B* expression before and after treatment, a linear mixed-effects model with the patient ID as a random effect was applied, using the “lme” function (nlme package, v3.1-163), followed by a Tukey's test post-hoc using the “glht” function (multcomp package, v1.4-29).

To identify pathways enriched among genes associated with NLRP3 and IL-1β expression that were downregulated by JAK1 inhibitors compared to Tofacitinib and

the control. DEGs were defined using a threshold of |l Log2 fold| > 0.3 and $p < 0.05$. The “Bioplanet2019” pathway database was used as the reference, and the enriched pathways were visualized using BarPlot.

Supplementary figures

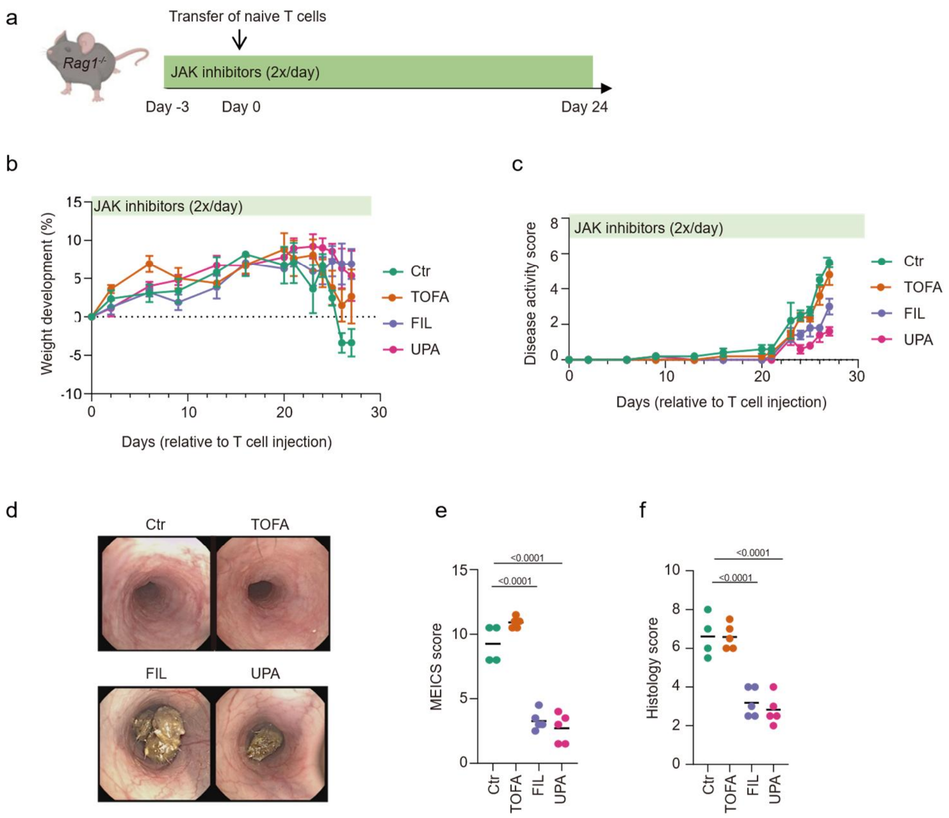

Supplementary Fig. 1. Upadacitinib and Filgotinib reduce T cell-mediated colitis. *Rag1*$^{-/-}$ mice were orally gavaged with the JAK inhibitors Tofacitinib (n=4), Filgotinib (n=4), Upadacitinib (n=4) starting three days prior to the injection of 0.25x10^{6} naïve T cells. **a** Experimental set-up (Created in BioRender. Bedke, T. (2026) https://BioRender.com/nqo42li); **b** weight development; **c** disease activity score over time; **d** representative images of colitis and **e** respective scoring of mouse endoscopy on the last day of the experiment; **f** scoring of disease severity of H&E-stained sections of the terminal colon. Every dot corresponds to one mouse. Bars represent mean value; error bars represent standard deviation (SD). Significance was assessed using two-tailed one-way ANOVA with Holm-Sidak's multiple comparison test.

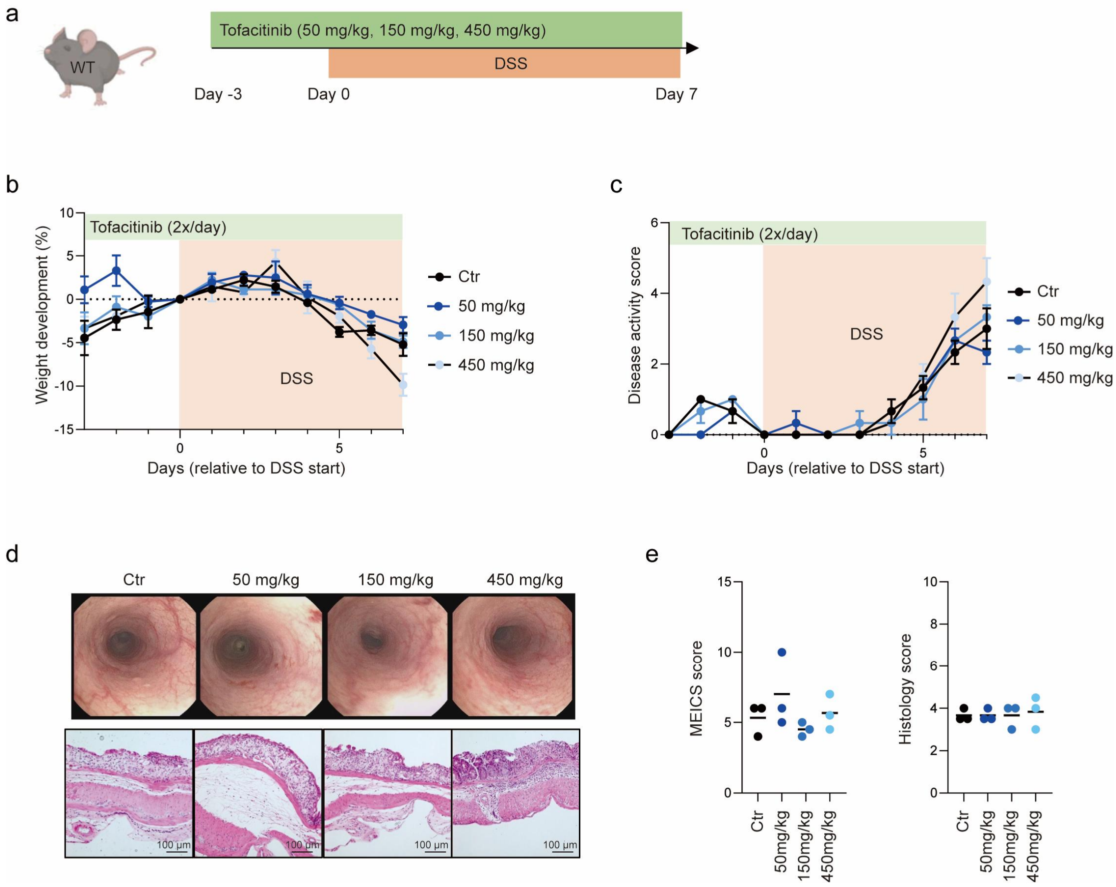

Supplementary Fig. 2. High doses of Tofacitinib are not effective in reducing colitis severity. WT mice were orally gavaged with the indicated doses of JAK inhibitor Tofacitinib starting three days prior to administration of 2% DSS in the drinking water for 7 days (n=3 per dose). **a** Experimental set-up (Created in BioRender. Bedke, T. (2026) https://BioRender.com/nqo42li); **b** weight development; **c** disease activity score over time; **d** representative images of mouse endoscopy (top) and H&E-stained sections of the terminal colon (bottom); **e** respective scoring of mouse endoscopy on the last day of the experiment and disease severity of H&E-stained sections of the terminal colon. Every dot corresponds to one mouse. Bars represent mean value; error bars represent standard deviation (SD).

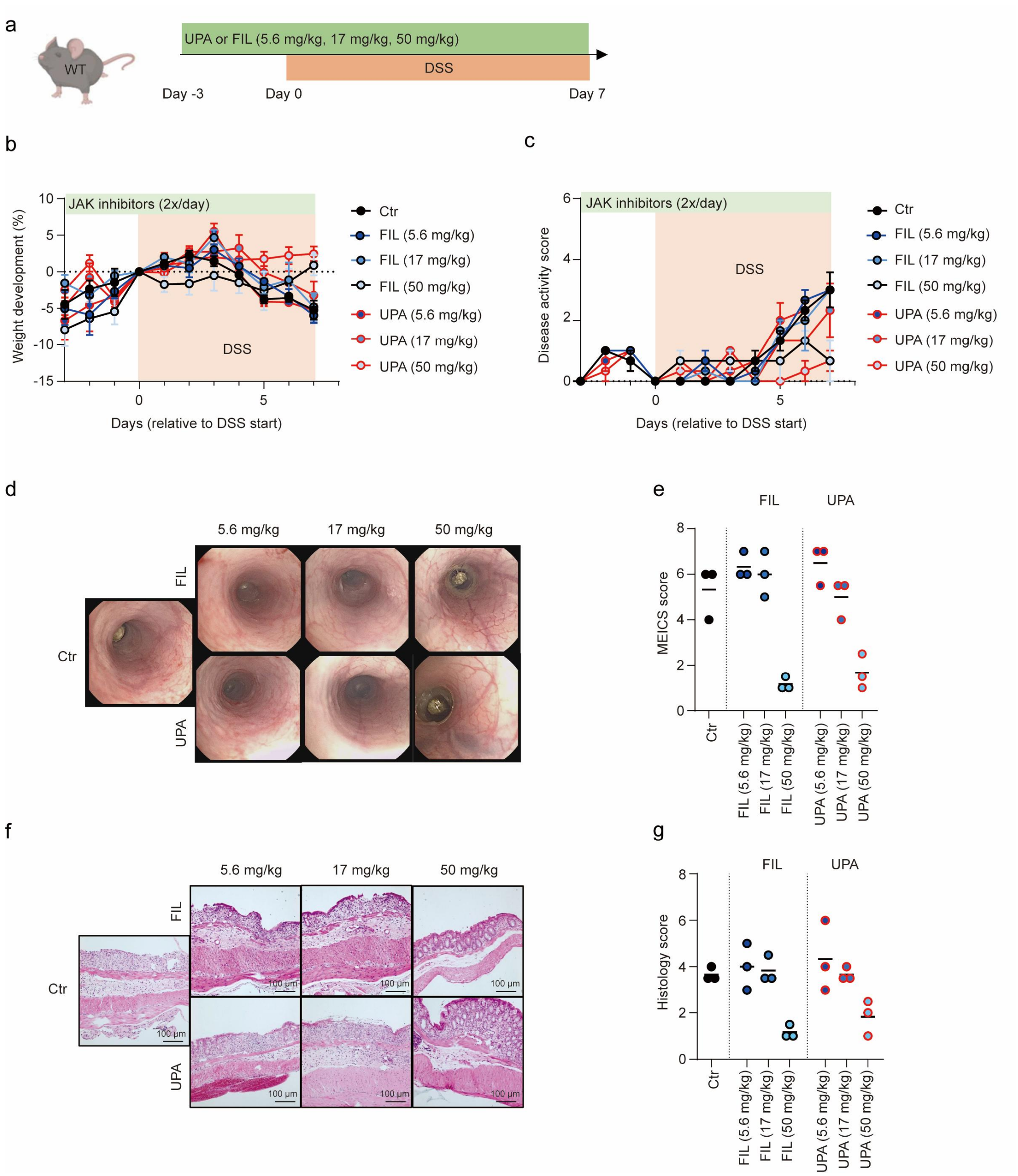

Supplementary Fig. 3. 50 mg/kg is the optimal dose for Upadacitinib and Filgotinib to reduce colitis severity. WT mice were orally gavaged with the JAK inhibitors Filgotinib and Upadacitinib at the indicated doses starting three days prior to administration of 2% DSS in the drinking water for 7 days (n=3 per dose). **a** Experimental set-up (Created in BioRender. Bedke, T. (2026) https://BioRender.com/nqo42li); **b** weight development; **c** disease activity score over time; **d** representative images; **e** respective scoring of mouse endoscopy on the last day of the experiment; **f** representative images; **g** scoring for disease severity of H&E-

stained sections of the terminal colon. Every dot corresponds to one mouse. Bars represent mean value; error bars represent standard deviation (SD).

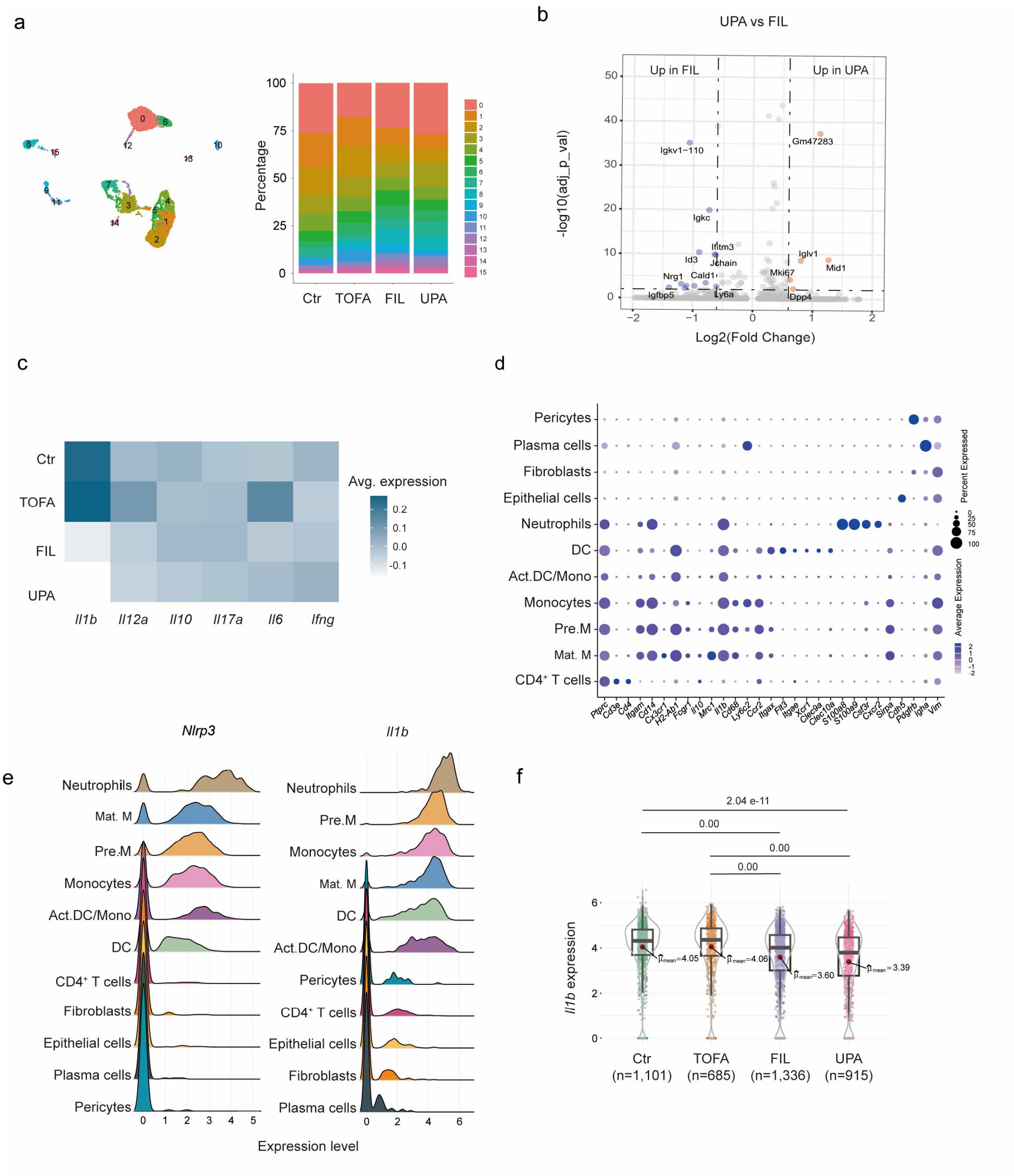

Supplementary Fig. 4. Single-cell RNA sequencing analysis of murine colonic immune cells. Mice treated with JAK inhibitors were sacrificed on day 5 after DSS started. CD4+ T cells and CD11b+ and/or CD11c+ cells were isolated and FACS-sorted from mouse colon samples and merged in a 1:1 ratio. Gene expression was examined by single cell analysis. **a** Left: Samples from control, Tofacitinib, Upadacitinib and Filgotinib groups were integrated and shown in a UMAP. Right: The composition of the clusters in every group. **b** DEGs between Upadacitinib and Filgotinib, statistics was assessed using two-tailed Wilcoxon Rank Sum test,

with the P-value adjusted by Benjamini–Hochberg false discovery rate (FDR) method for multiple comparisons; **c** average expression of the indicated genes among groups; **d** dot plot of characteristic markers in different cell clusters; **e** *Il1b* and *Nlrp3* expression among cell clusters; **f** average expression of *Il1b* in myeloid cells. The Center line, lower and upper hinges correspond to the median, 25th and 75th percentiles. The upper whisker extends from the hinge to the largest value no further than 1.5 * IQR from the hinge (where IQR is the inter-quartile range, or distance between the first and third quartiles). The lower whisker extends from the hinge to the smallest value at most 1.5 * IQR of the hinge. P-value was adjusted by Holm-Bonferroni method.

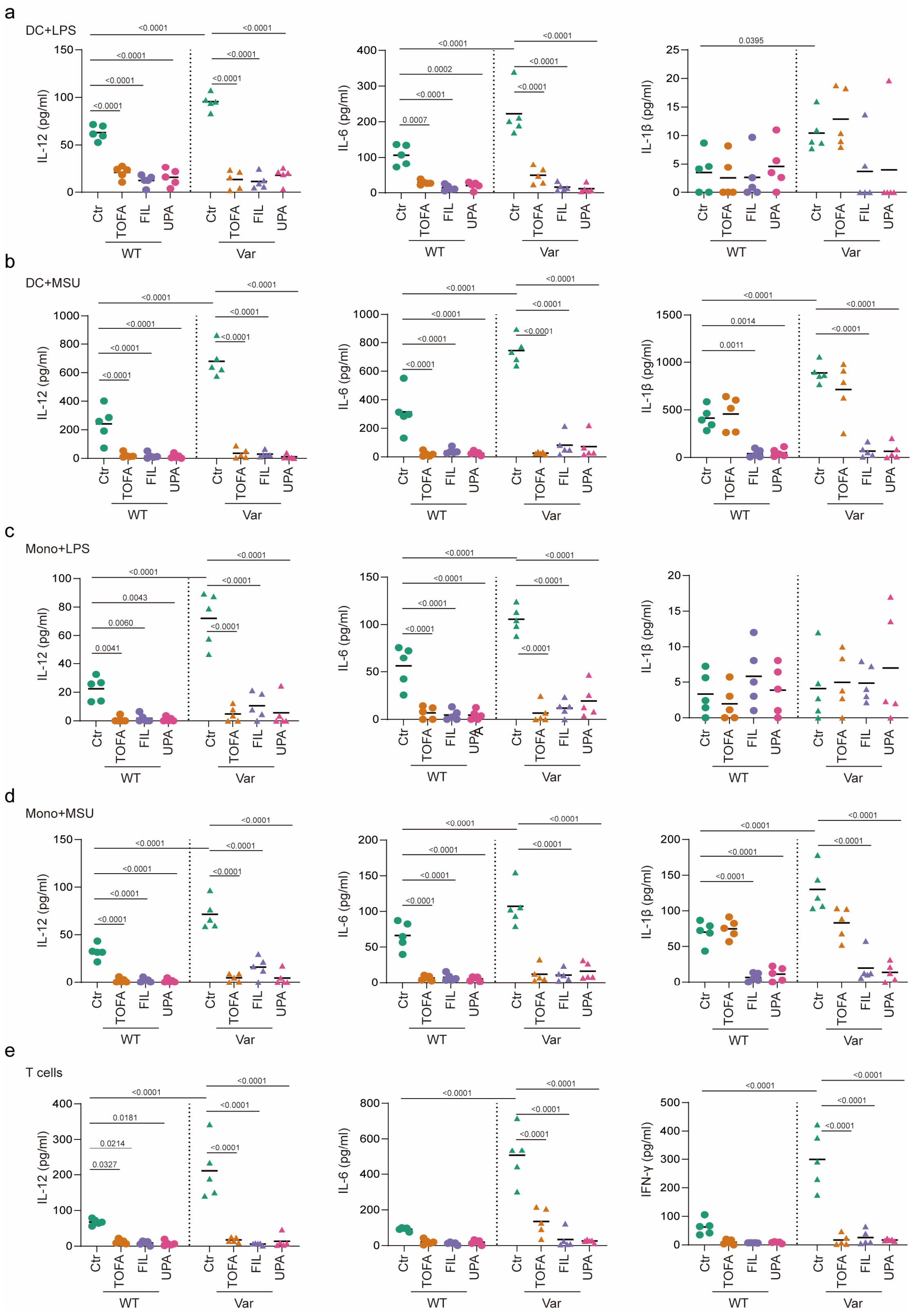

Supplementary Fig. 5. Upadacitinib and Filgotinib inhibit cytokine production in monocytes, DCs and T cells. Monocytes and T cells were isolated from the peripheral blood of IBD patients who were either WT (n=5) or variant (n=5) for the loss-of-function SNP

rs1893217 (n=5) in the gene locus encoding PTPN2. Monocytes or monocyte-derivd DCs were then treated for 24h with LPS with or without futher activation with MSU crystals (150 ng/ml) for 6h. T cells were activted by anti-CD3/anti-CD28 dynabeads. Cytokine levels in the supernatant for the indicated cytokines produced by **a-b** DCs, **c-d** monocytes and **e** T cell. Every dot corresponds to one individual. Bars represent mean value. Significance was assessed using two-tailed two-way ANOVA with Tukey's multiple comparisons test.

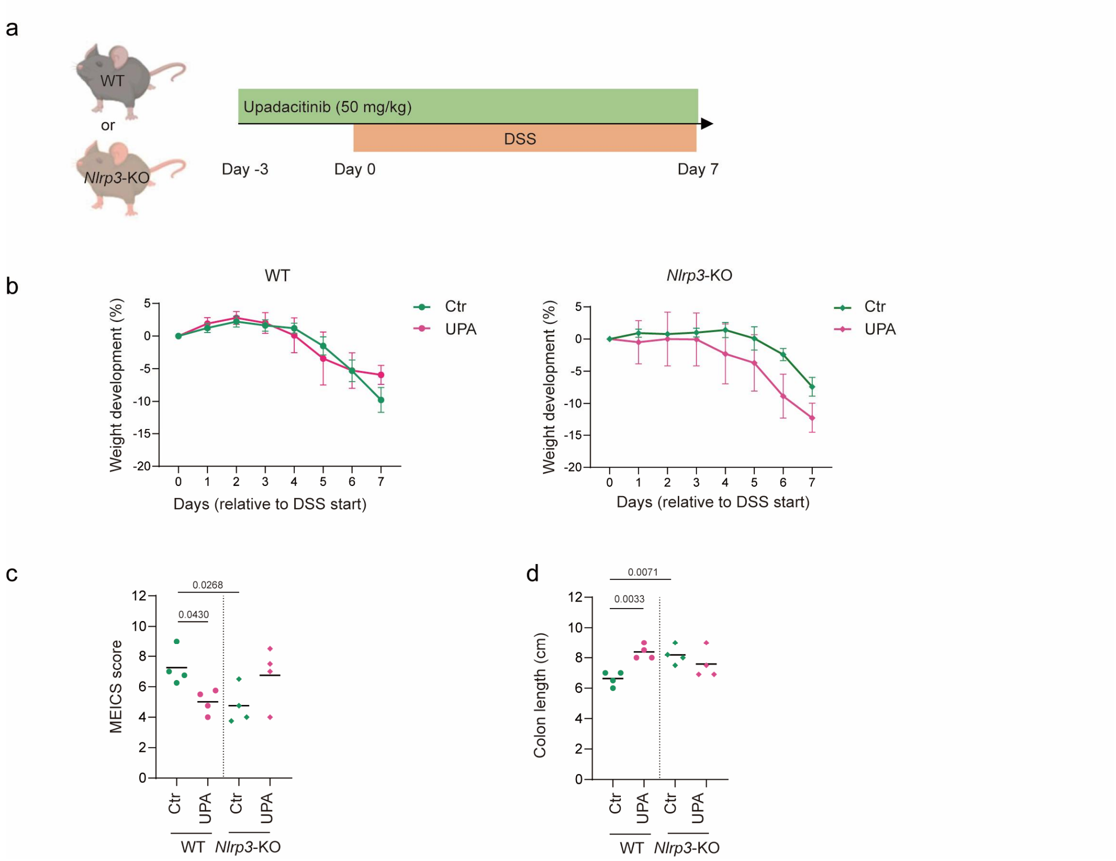

Supplementary Fig. 6. The protective effect of Upadacitinib is abrogated in NLRP3-deficient mice. NLRP3-deficient mice or WT littermates were treated with the Upadacitinib or vehicle control starting three days prior to administration of 2% DSS in the drinking water for 7 days. **a** Experimental set-up (Created in BioRender. Bedke, T. (2026) https://BioRender.com/nqo42li); **b** weight development; **c** respective scoring of mouse endoscopy on the last day of the experiment; **d** colon length. Results are from one experiment. Every dot in C and D corresponds to one mouse (n=4 per group). Bars represent mean value; error bars represent standard deviation (SD). Significance was assessed using two-tailed two-way ANOVA test with Tukey's multiple comparisons test.

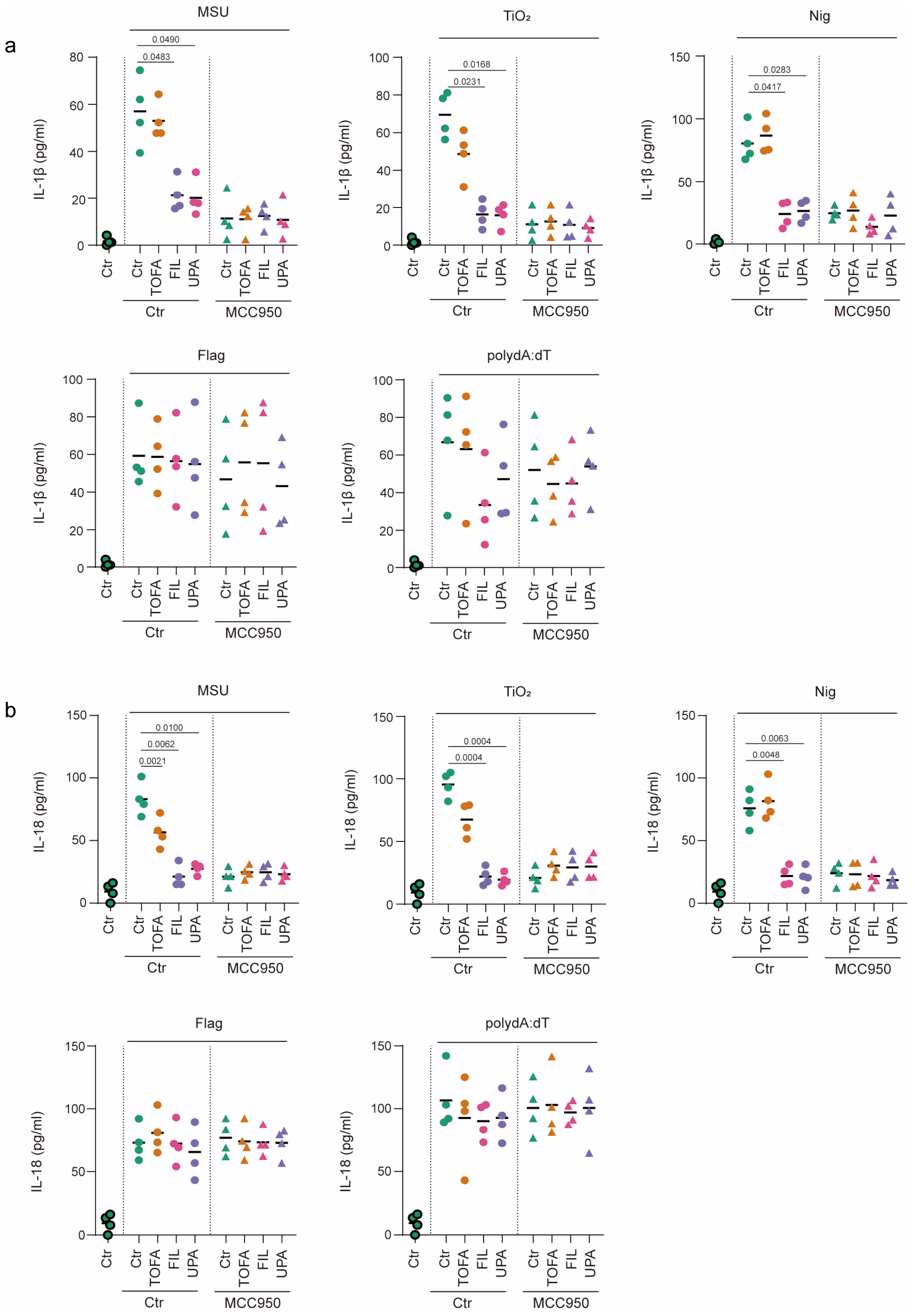

Supplementary Fig. 7. No additive effect of NLRP3 inhibitor MCC950 and JAK1-inhibitors. Monocytes were isolated from the peripheral blood of healthy volunteers (n=4) and differentiated into macrophages for 7 days. Macrophages were treated for 24 h with LPS and

then activated with MSU (150ng/ml, 6 h), TiO2 (150 ng/ml, 12 h), Nigericin (200ng/ml, 6 h), Flagellin (Flag, 500ng/ml, 2 h) or poly(dA:dT) (1ug/ml, 2h). Cell culture supernatant was assessed for **a** IL-1β and **b** IL-18 by ELISA (R&D Systems). Every dot corresponds to one individual. Bars represent mean value. Significance was assessed using two-tailed one-way ANOVA with Holm-Sidak's multiple comparisons test.

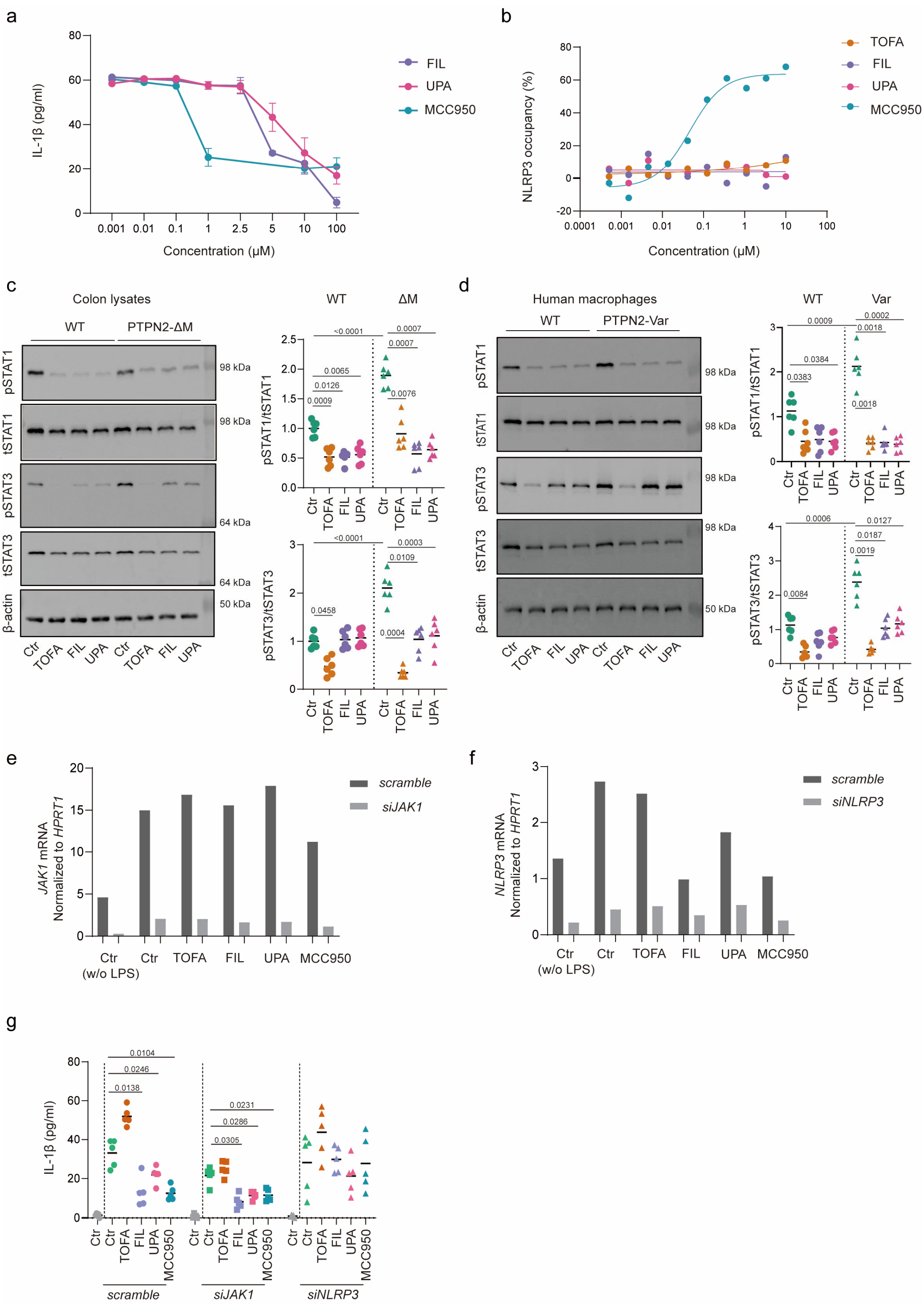

Supplementary Fig. 8. JAK1 inhibitors control NLRP3 indirectly. **a** Human monocyte-derived macrophages were activated with LPS in the presence of Filgotinib, Upadacitinib or MCC950 at indicated concentrations. IL-1β in the supernatant was measured

by ELISA. **b** Occupancy signal of NLRP3 in the presence of JAK inhibitors and MCC950 as measured using the NLRP3 NanoBRET™ Target Engagement. **c-d** Western blot quantification of phosphorylation of STAT1 and STAT3 in mouse colon lysates from WT (n=6) and PTPN2-ΔM mice (n=6), as well as human macrophages from healthy controls (n=6) and individuals carrying PTPN2 variant (n=6). Every dot corresponds to one mouse or one individual. Each data point represents one individual. Bars represent mean value. Significance was assessed using two-tailed two-way ANOVA with Tukey's multiple comparisons test. **e-g** Monocytes were isolated from buffy coats of healthy volunteers, and differentiated to macrophages with the presence of M-CSF. On day 6, macrophages were transfected by *siJAK1*, *siNLRP3* or scramble control for 48 h, then activated by LPS and treated with Tofacitinib, Upadacitinib, Filgotinib or MCC950. mRNA expression levels of **e** JAK1 and **f** NLRP3 were quantified by qPCR under respective conditions. **g** IL-1β production in the supernatant assessed by ELISA (n=5), biological repetitions). Bars represent mean value; error bars represent standard deviation (SD). Significance was assessed using two-tailed one-way ANOVA with Holm-Sidak's multiple comparison test.

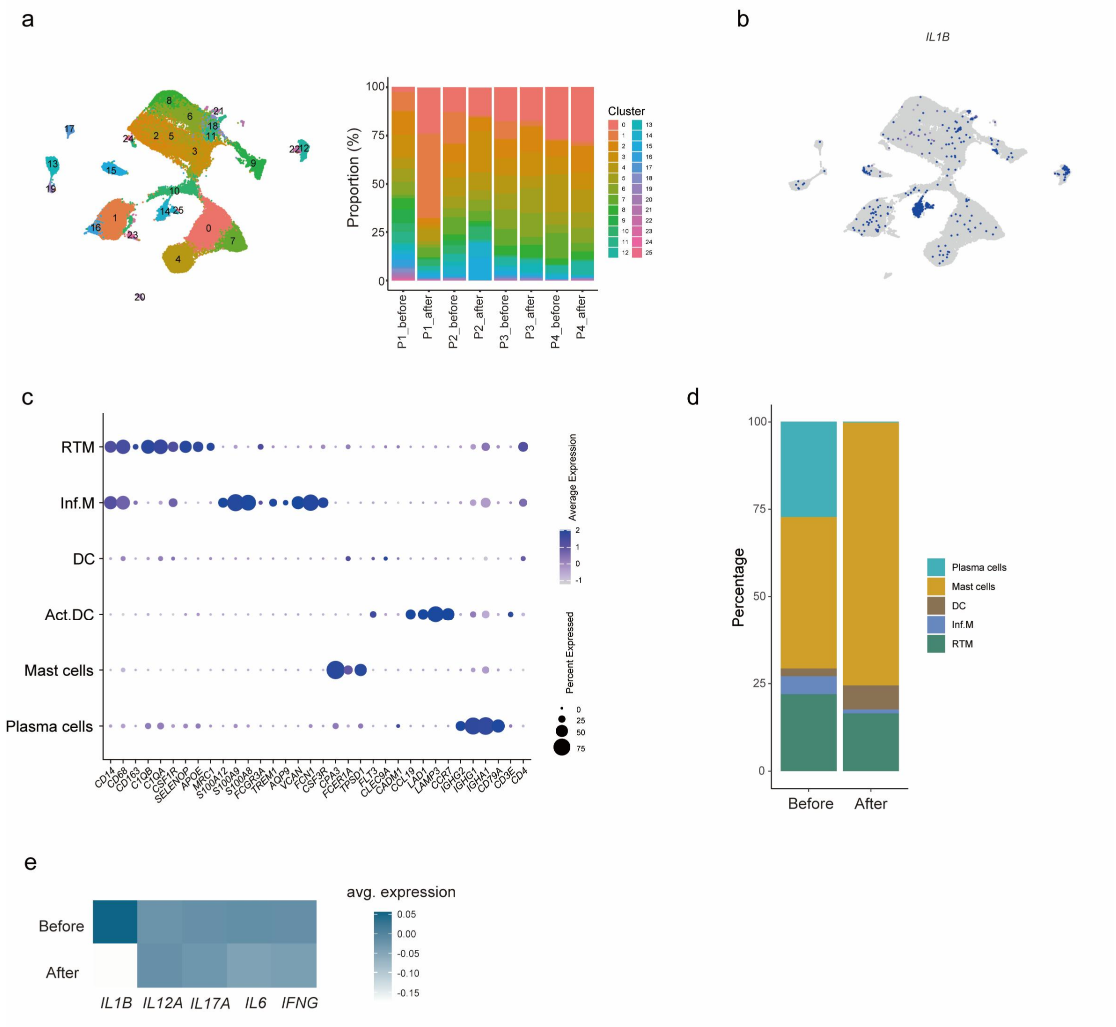

Supplementary Fig. 9. Clusters and gene expression of the cells from UC patients. Cells in lamina propria from patients before and after Upadacitinib treatment were isolated, and gene expression was determined by RNA single cell analysis **a** Left: Cell clusters from all integrated samples shown in UMAP. Right: The composition of clusters in all samples. **b** *IL1B* expression in all clusters; **c** Featured genes of identified clusters expressing *IL1B*; **d** Proportions of the myeloid clusters in patients before and after treatment; **e** Average expression of indicated genes in myeloid cells before and after Upadacitinib treatment.

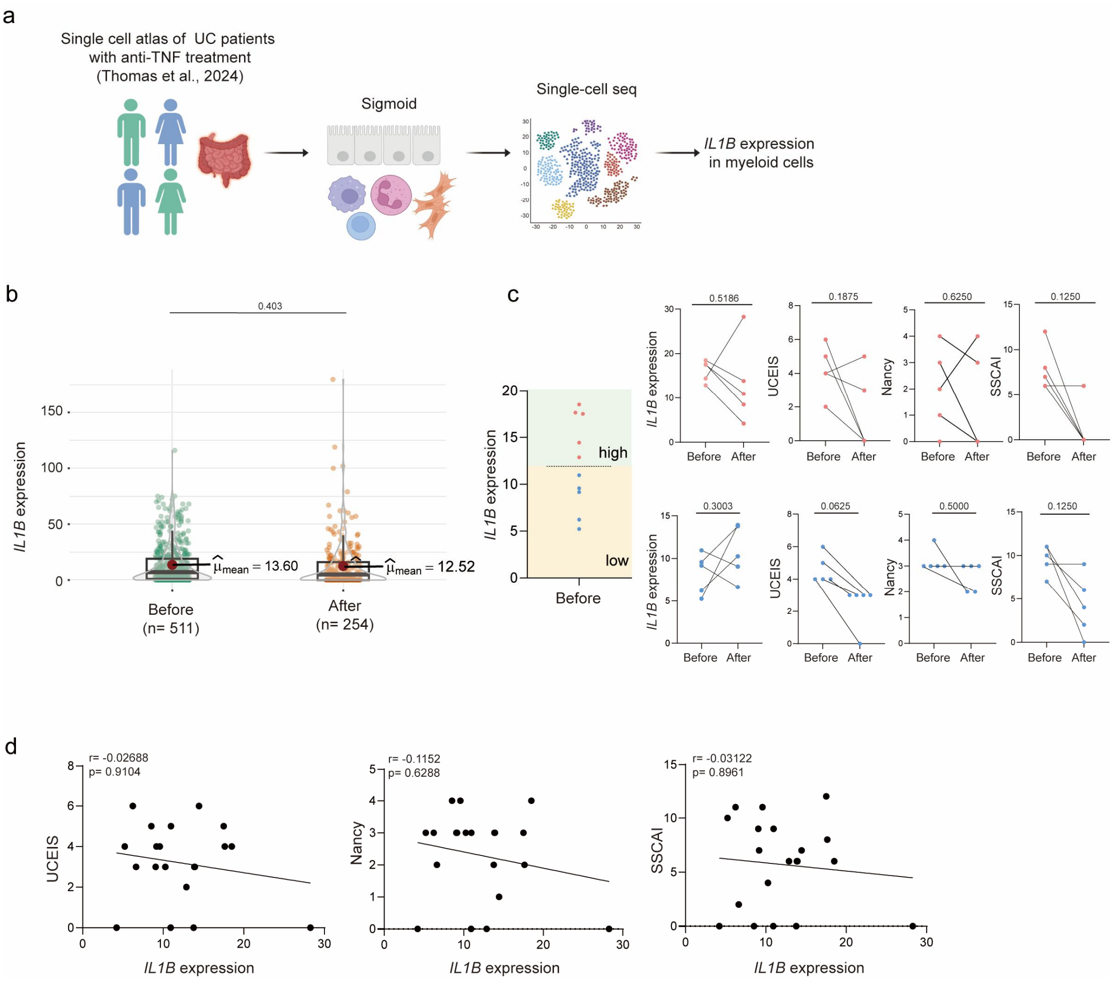

Supplementary Fig. 10. *IL1B* expression in UC patients with anti-TNF treatment. *IL1B* expression was assessed in myeloid cells from the single cell atlas (Thomas et al., 2024) with 10 UC patients before and after Adalimumab. **a** Analysis set-up (Created in BioRender. Bedke, T. (2026) https://BioRender.com/nqo42li); **b** Violin Plot of *IL1B* expression in patients with remission (n=4) before and after Adalimumab treatment. Significance was assessed using a two-tailed linear mixed-effects model with the patient ID as a random effect, followed by a Tukey's post-hoc test. The Center line, lower and upper hinges correspond to the median, 25th and 75th percentiles. The upper whisker extends from the hinge to the largest value no further than 1.5 * IQR from the hinge (where IQR is the inter-quartile range, or distance between the first and third quartiles). The lower whisker extends from the hinge to the smallest value at most 1.5 * IQR of the hinge. **c** *IL1B* expression and colitis assessment in the individual UC patient (n=10). Left: *IL1B* expression of patients before Adalimumab treatment. Right upper lane: Patients with high *IL1B* expression and their clinical colitis evaluation. Right lower lane: Patients

with low *IL1B* expression and their clinical colitis evaluation. UCEIS, Ulcerative Colitis Endoscopic Index of Severity; Nancy, histopathological Nancy index; SSCAI, Simple Clinical Colitis Activity Index. Significance was assessed using two-tailed Wilcoxon matched-pairs signed rank test. **d** Correlation of *IL1B* expression with colitis scores in Adalimumab treatment. Correlation was tested by nonparametric Spearman correlation. Every dot corresponds to one individual in C and D.

Supplementary Table 1. Clinical characters of patients with Upadacitinib treatment

	Gender	Age	Mayo score (pre-treatment)	Mayo score (post-treatment)
Patient 1-16	Female (37.5%), male (62.5%)	45.2±12.73	5.44±1.97	3±1.75

Supplementary Table 2. Clinical characters of patients with anti-TNF treatment

Sample ID	SSCAI	UCEIS	Nancy
	Before/after Adalimumab treatment		
UC2	6/6	4/3	4/3
UC5*	12/0	5/0	3/0
UC6	11/4	6/3	3/3
UC7	9/9	5/3	3/3
UC8	8/0	4/5	2/4
UC9	7/2	4/3	3/2
UC10*	11/0	4/0	4/2
UC12*	6/0	2/0	3/0
UC13*	7/0	6/0	1/0
UC14	10/6	4/3	3/3

*Patients with remission

Western blots in Supplementary Fig. 8C

Mouse

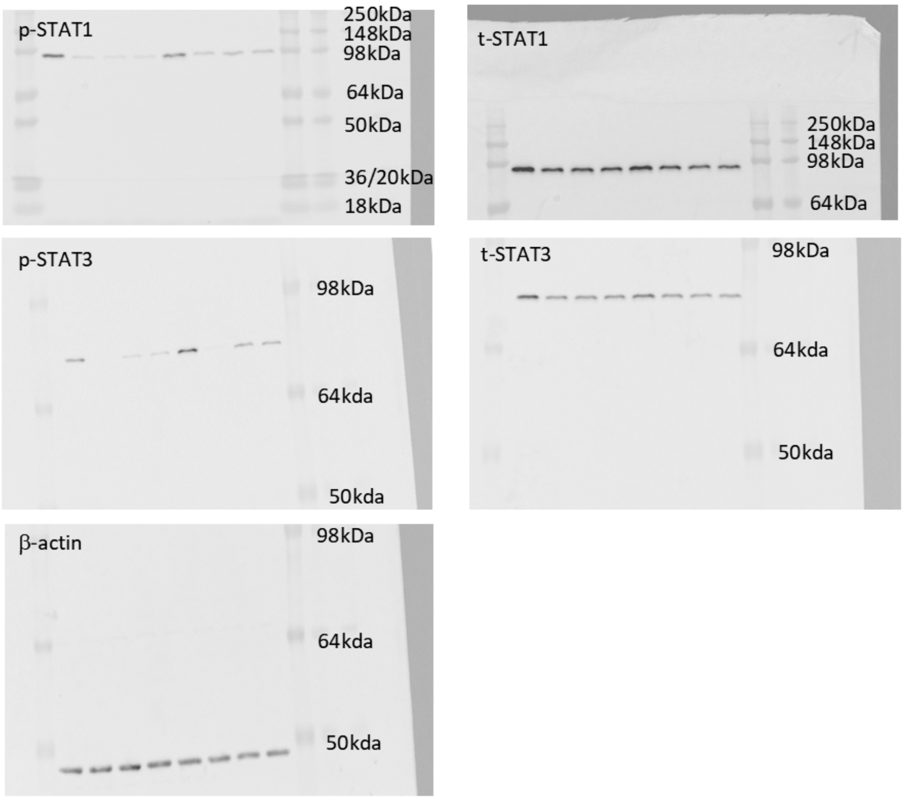

Human

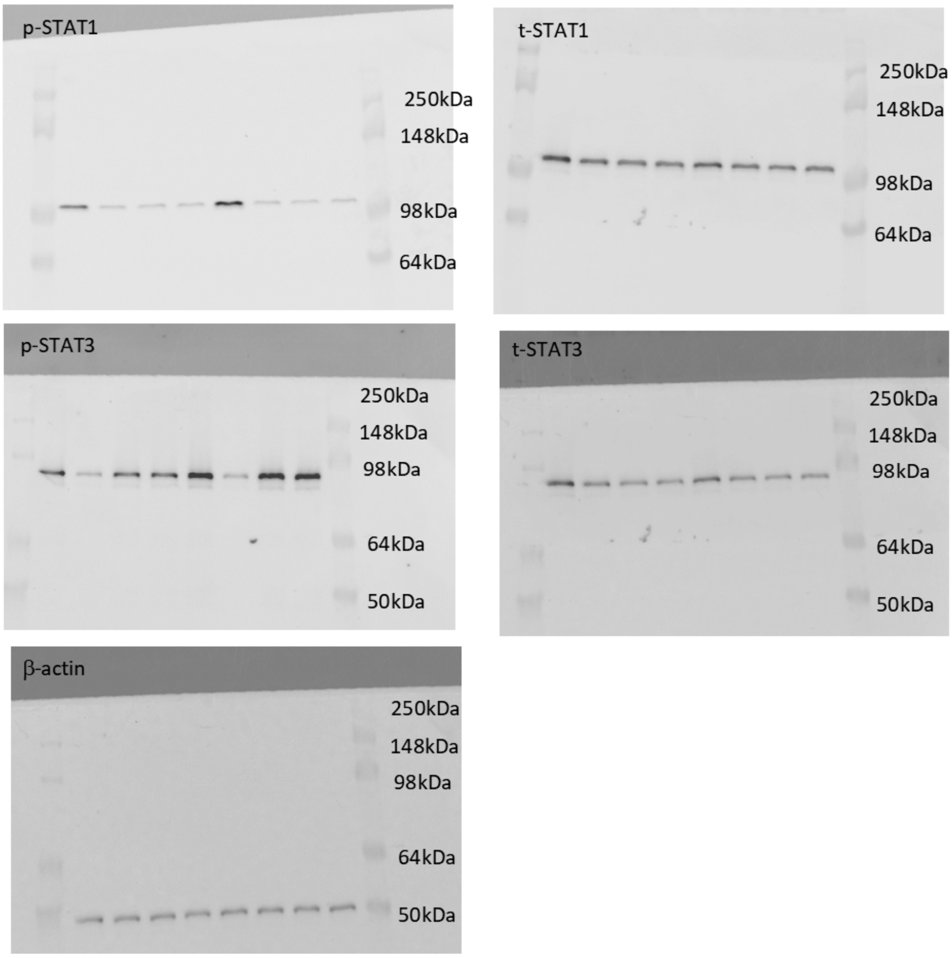
p-STAT1
250kDa
148kDa
98kDa
64kDa
t-STAT1
250kDa
148kDa
98kDa
64kDa
p-STAT3
250kDa
148kDa
98kDa
64kDa
50kDa
t-STAT3
250kDa
148kDa
98kDa
64kDa
50kDa
β-actin
250kDa
148kDa
98kDa
64kDa
50kDa
