# Supplementary material for: Indirect inhibition of the NLRP3-interleukin-1β axis contributes to the efficacy of JAK1 inhibitors in experimental colitis and human ulcerative colitis
Source: Nat Commun. 2026 Apr 18;17:5429. doi: 10.1038/s41467-026-71808-y (PMC13279951; doi:10.1038/s41467-026-71808-y)
Supplement: Supplementary file 2 — Reporting Summary [file 41467_2026_71808_MOESM2_ESM.pdf]

nature portfolio

Corresponding author(s): Samuel Huber/Michael Scharl

Last updated by author(s): Jan 30, 2026

Reporting Summary

Nature Portfolio wishes to improve the reproducibility of the work that we publish. This form provides structure for consistency and transparency in reporting. For further information on Nature Portfolio policies, see our Editorial Policies and the Editorial Policy Checklist.

Statistics

For all statistical analyses, confirm that the following items are present in the figure legend, table legend, main text, or Methods section.

n/a	Confirmed	
☐	☒	The exact sample size (*n*) for each experimental group/condition, given as a discrete number and unit of measurement
☐	☒	A statement on whether measurements were taken from distinct samples or whether the same sample was measured repeatedly
☐	☒	The statistical test(s) used AND whether they are one- or two-sided<br>*Only common tests should be described solely by name; describe more complex techniques in the Methods section.*
☒	☐	A description of all covariates tested
☐	☒	A description of any assumptions or corrections, such as tests of normality and adjustment for multiple comparisons
☐	☒	A full description of the statistical parameters including central tendency (e.g. means) or other basic estimates (e.g. regression coefficient) AND variation (e.g. standard deviation) or associated estimates of uncertainty (e.g. confidence intervals)
☐	☒	For null hypothesis testing, the test statistic (e.g. *F*, *t*, *r*) with confidence intervals, effect sizes, degrees of freedom and *P* value noted<br>*Give P values as exact values whenever suitable.*
☒	☐	For Bayesian analysis, information on the choice of priors and Markov chain Monte Carlo settings
☒	☐	For hierarchical and complex designs, identification of the appropriate level for tests and full reporting of outcomes
☐	☒	Estimates of effect sizes (e.g. Cohen's *d*, Pearson's *r*), indicating how they were calculated

Our web collection on statistics for biologists contains articles on many of the points above.

Software and code

Policy information about availability of computer code

Data collection	Cell Ranger software pipeline (v5.1.0, 10X Genomics); BioTek Gen5 Software (ELISA data); ImageJ/Fiji (Western blot);
Data analysis	GraphPad Prism 10, R studio (v2023.12.0),Seurat package (v5.0.1),ggplot2 package (v3.5.0); ggstatsplot package (v0.12.4); patchword package (v1.2.0); ggrepel package (v0.9.5), harmony package (v1.2.0), nlme package (v3.1-163), multcomp package (v1.4-29), EnrichR platform, QuPath (v0.5.1)

For manuscripts utilizing custom algorithms or software that are central to the research but not yet described in published literature, software must be made available to editors and reviewers. We strongly encourage code deposition in a community repository (e.g. GitHub). See the Nature Portfolio guidelines for submitting code & software for further information.

Data

Policy information about availability of data

All manuscripts must include a data availability statement. This statement should provide the following information, where applicable:

- Accession codes, unique identifiers, or web links for publicly available datasets
- A description of any restrictions on data availability
- For clinical datasets or third party data, please ensure that the statement adheres to our policy

Source data are provided with this paper. Mouse single-cell RNA-seq raw and processed data have been deposited in the database (DOI: 10.25592/uhhfdm.18257). Human RNA-seq raw data are available under restricted access due to the data privacy. Access can be obtained after signing a “Data transfer agreement” by contacting Prof. Samuel Huber (s.huber@uke.de). The anonymous processed human data have been deposited in the database (DOI: 10.25592/

uhhfdm.18257).Single-cell RNA-seq data from Thomas et al. reanalysed in this study are publicly accessible via the original publication.

Research involving human participants, their data, or biological material

Policy information about studies with human participants or human data. See also policy information about sex, gender (identity/presentation), and sexual orientation and race, ethnicity and racism.

Reporting on sex and gender	6 female and 10 male patients were enrolled in the study.
Reporting on race, ethnicity, or other socially relevant groupings	Race, ethnicity or other socially relevant grouping variables were not analyzed in the study.
Population characteristics	Human participants enrolled in this study were healthy volunteers or IBD patients from 30 to 62 years of age.
Recruitment	IBD patients who were variant for the IBD-associated PTPN2 SNP rs1893217, and patients diagnosed with ulcerative colitis and treated with Upadacitinib were enrolled.
Ethics oversight	Ethics approval: PV7106, Ethikkommission der Ärztekammer Hamburg, Germany; EK-1755, Cantonal Ethics Commission Zurich, Switzerland.

Note that full information on the approval of the study protocol must also be provided in the manuscript.

Field-specific reporting

Please select the one below that is the best fit for your research. If you are not sure, read the appropriate sections before making your selection.

[x] Life sciences [ ] Behavioural & social sciences [ ] Ecological, evolutionary & environmental sciences

For a reference copy of the document with all sections, see nature.com/documents/nr-reporting-summary-flat.pdf

Life sciences study design

All studies must disclose on these points even when the disclosure is negative.

Sample size	In the animal experiments, sample size was chosen based on the previous work from our group using the same models. Based on the previous data, we conducted G-power analysis with an ANOVA design to determine the sample size. Regarding human data, no sample size was calculated.
Data exclusions	No data point were excluded from the analyses
Replication	Data presented in animal studies were combined data from at least two independent experiments. All replication attempts were successful.
Randomization	In mouse experiments, animals from the same genotype were randomly distributed into different treatment groups.
Blinding	The investigators were blinded for the treatment groups during the experiment and the subsequent analyses. Blinding was not applied to the single-cell RNA-seq analyses.

Reporting for specific materials, systems and methods

We require information from authors about some types of materials, experimental systems and methods used in many studies. Here, indicate whether each material, system or method listed is relevant to your study. If you are not sure if a list item applies to your research, read the appropriate section before selecting a response.

Materials & experimental systems

n/a	Involved in the study	
[ ]	[x]	Antibodies
[ ]	[x]	Eukaryotic cell lines
[x]	[ ]	Palaeontology and archaeology
[ ]	[x]	Animals and other organisms
[x]	[ ]	Clinical data
[x]	[ ]	Dual use research of concern
[x]	[ ]	Plants

Methods

n/a	Involved in the study	
[x]	[ ]	ChIP-seq
[ ]	[x]	Flow cytometry
[x]	[ ]	MRI-based neuroimaging

Antibodies

Antibodies used	Live/dead staining by Fixable Viability Dye eFluorTM 506 (Amcyan)(eBioscience);

anti-mouse CD45 BV785 (Biolegend, Cat.:103149, Clone: 30-F11, Lot: B336128);
anti-mouse CD3 BV650 (Biolegend, Cat.:100229, Clone:17A2, Lot: B366948);
anti-mouse CD4 PE-Cy7 (Biolegend, Cat.:100528, Clone:RM4-5, Lot: B262765);
anti-mouse CD4-APC-Cy7 (DB Biosciences, Cat: 552051, clone GK1.5)
anti-mouse CD11b PE (Biolegend, Cat.: 101208, Clone: M1/70, Lot: B166034);
anti-mouse CD11c PE (BD Biosciences, Cat.: 553802, Clone:HL3, Lot: 40263);
anti-mouse CD19 APC-Cy7 (Biolegend, Cat.: 115530, Clone: 6D5, Lot: B253924);
anti-mouse CD44-APC (eBioscience, Cat: 17-0441-83, clone IM7)
anti-mouse CD62L-FITC (BioLegend, Cat: 104406, clone: MEL-14)
anti-mouse B220-PE-Cy7 (BD Pharmingen, Cat: 552772, clone RA3-6B2)
anti-mouse CD8-PerCP-Cy5.5 (eBioscience, Cat: 45-0081-80, clone 53-6.7)
anti-mouse IL-1β (R&D/bio-Techne, Cat: AF-401-NA);
anti-human CD45 PE-Cy7 (Biolegend, Cat.: 304016, Clone: HI30, Lot: B210429);
anti-human IL-1β (R&D Systems, Cat.: AF-201-SP);
anti-human/mouse Caspase-1 (AdipoGen, AG-20B-0042-C100);
anti-b-actin (Millipore, Cat: MAB1501).

Validation

Live/dead staining by Fixable Viability Dye eFluorTM 506 : https://www.thermofisher.com/order/catalog/product/65-0866-14;
anti-mouse CD45 BV785:https://www.biolegend.com/en-us/products/brilliant-violet-785-anti-mouse-cd45-antibody-10636;
anti-mouse CD3 BV650: https://www.biolegend.com/en-us/products/brilliant-violet-650-anti-mouse-cd3-antibody-7843;
anti-mouse CD4 PE-Cy7: https://www.biolegend.com/en-us/products/pe-cyanine7-anti-mouse-cd4-antibody-1932;
anti-mouse CD4-APC-Cy7:https://www.bdbiosciences.com/en-de/products/reagents/flow-cytometry-reagents/research-reagents/single-color-antibodies-ruo/apc-cy-7-rat-anti-mouse-cd4.552051?tab=product_details;
anti-mouse CD11b PE: https://www.biolegend.com/en-us/products/pe-anti-mouse-human-cd11b-antibody-349;
anti-mouse CD11c PE:https://www.bdbiosciences.com/en-de/products/reagents/flow-cytometry-reagents/research-reagents/single-color-antibodies-ruo/pe-hamster-anti-mouse-cd11c.553802?tab=product_details;
anti-mouse CD19 APC-Cy7: https://www.biolegend.com/en-us/products/apc-cyanine7-anti-mouse-cd19-antibody-3903;
anti-mouse CD44-APC: https://www.thermofisher.com/antibody/product/CD44-Antibody-clone-IM7-Monoclonal/17-0441-83;
anti-mouse CD62L-FITC: https://www.biolegend.com/en-us/products/fitc-anti-mouse-cd62l-antibody-384;
anti-mouse B220-PE-Cy7: https://www.bdbiosciences.com/en-de/products/reagents/flow-cytometry-reagents/research-reagents/single-color-antibodies-ruo/pe-cy-7-rat-anti-mouse-cd45r-b220.552772?tab=product_details;
anti-mouse CD8-PerCP-Cy5.5: https://www.thermofisher.com/antibody/product/CD8a-Antibody-clone-53-6-7-Monoclonal/45-0081-80;
anti-mouse IL-1β: https://www.rndsystems.com/products/mouse-il-1beta-il-1f2-antibody_af-401-na;
anti-human CD45 PE-Cy7: https://www.biolegend.com/en-us/products/pe-cyanine7-anti-human-cd45-antibody-1915;
anti-human IL-1β: https://www.rndsystems.com/products/human-il-1beta-il-1f2-antibody_af-201-na;
anti-human/mouse Caspase-1: https://adipogen.com/ag-20b-0042-anti-caspase-1-p20-mouse-mab-casper-1.html;
anti-b-actin:https://www.merckmillipore.com/DE/de/product/Anti-Actin-Antibody-clone-C4,MM_NF-MAB1501?ReferrerURL=https%3A%2F%2Fwww.google.com%2F;

Eukaryotic cell lines

Policy information about cell lines and Sex and Gender in Research

Cell line source(s)	Human HEK293 cells (female origin), ATCC
Authentication	Cell identity is validated with STR analysis
Mycoplasma contamination	Cells were tested negative for mycoplasma contamination
Commonly misidentified lines (See ICLAC register)	HEK293 cells were obtained from ATCC and authenticated by STR profiling; no commonly misidentified cell lines listed in the ICLAC register were used.

Animals and other research organisms

Policy information about studies involving animals; ARRIVE guidelines recommended for reporting animal research, and Sex and Gender in Research

Laboratory animals

Mice on the C57BL/6 background were used for all experiments. All mice were kept in single-ventilated cages in specific-pathogen-free conditions with 12 hours dark/light cycle, standard animal chow and water ad libitum, ambient temperature of 20±2°C, humidity of 55±10%. For colitis models involving PTPN2-ΔM mice and their WT littermates, Rag1-/- mice, as well as Caspase-1 KO and WT mice, females at a weight of 20-25 g were used for the experiments. In the colitis experiments with NLRP3 KO and their WT littermates, both male and female mice were included.

Wild animals	No wild animals were used in this study.
Reporting on sex	Main experiments were conducted with female mice in this study as male mice become aggressive when treated with DSS.
Field-collected samples	The study did not involve samples collected in the field.
Ethics oversight	The animal welfare commission of the canton of Zurich and Behörde für Justiz und Verbraucherschutz Hamburg approved all animal experiments included in this study (License Nr ZH127/2022, N111/2025).

Note that full information on the approval of the study protocol must also be provided in the manuscript.

Plants

Seed stocks	not applicable
Novel plant genotypes	not applicable
Authentication	not applicable

Flow Cytometry

Plots

Confirm that:

- [ ] The axis labels state the marker and fluorochrome used (e.g. CD4-FITC).
- [ ] The axis scales are clearly visible. Include numbers along axes only for bottom left plot of group (a 'group' is an analysis of identical markers).
- [ ] All plots are contour plots with outliers or pseudocolor plots.
- [ ] A numerical value for number of cells or percentage (with statistics) is provided.

Methodology

Sample preparation	Cells were sorted using fluorescence-activated cell sorting to enrich target cell population for single-cell RNA-seq analysis. Cell isolation from murine colon: Colon tissue was digested in dithioerythritol (DTT) buffer for 20 minutes at 37°C on a shaker and were subsequently incubated in collagenase buffer for 30 minutes at 37°C on a shaker. Next, immune cells were separated using a density gradient consisting of 40% and 60% Percoll. Interface was collected, washed and resuspended. Cells for cytokine measurement were tested freshly. Cells for single-cell RNA-seq were frozen and thawed before loading chips.<br>Cell isolation from human lamina propria: tissue was digested in DTT buffer for 15 minutes at 37°C on a shaker, then further cut and digested for 30 min in RPMI 1640 medium containing DNase I (2ug/ml, Roche, Switzerland) and Collagenase (1mg/ml) from Clostridium histolyticum (Roche, Switzerland) at 37°C on a shaker. A total of 10,000 LP cells and 20,000 FACS sort-enriched CD45+ cells were counted and loaded respectively in the Chromium Next GEM Chip K.
Instrument	BD FACSAria Fusion cell sorter
Software	BD Diva
Cell population abundance	Cell enrichment for mouse single cell RNA sequencing: CD11b+ and/or CD11c+ cells and CD4+ T cells were all sorted individually.<br>Cell enrichment for human single cell RNA sequencing: CD45+ cells were sorted.<br>The abundance within post-sort reached 95% or above.
Gating strategy	Mouse: live CD3- CD19- CD11b+ and/or CD11c+ cells and CD3+ CD4+ T cells were sorted. Human: live CD45+ cells were sorted.

- [ ] Tick this box to confirm that a figure exemplifying the gating strategy is provided in the Supplementary Information.
