# Supplementary material for: Indirect inhibition of the NLRP3-interleukin-1β axis contributes to the efficacy of JAK1 inhibitors in experimental colitis and human ulcerative colitis
Source: Nat Commun. 2026 Apr 18;17:5429. doi: 10.1038/s41467-026-71808-y (PMC13279951; doi:10.1038/s41467-026-71808-y)
Supplement: Supplementary file 3 — Transparent Peer Review file [file 41467_2026_71808_MOESM3_ESM.pdf]

nature portfolio

Peer Review File

Indirect inhibition of the NLRP3-interleukin-1β axis contributes to the efficacy of JAK1 inhibitors in experimental colitis and human ulcerative colitis

Corresponding Author: Professor Samuel Huber

This file contains all reviewer reports in order by version, followed by all author rebuttals in order by version.

Version 0:

Reviewer comments:

Reviewer #1

(Remarks to the Author)
In this original article, the authors take on the important task of trying to dissect the mechanism of action of JAK inhibitors. In particular, they attempted to verify whether 3 different Jak inhibitors (i.e. Tofacitinib, Upadacitinib, and Filgotinib), which are used to treat human inflammatory bowel diseases, differ in their ability to modulate pathogenic pathways. The authors show that Upadacitinib and Filgotinib but not Tofacitinib inhibit the inflammasome activation and prevent experimental colitis induced by giving mice oral dextran sulfate sodium, a sugar that causes gut epithelial cell injury.
The data presented in this paper are largely incomplete and leave many open questions. The paper seems to show no difference between Filgotinib and Upadacitinib in terms of inflammasome inhibition and colitis prevention, but the response and remission rates of these two compounds in IBD patients are largely different, and Upadacitinib but not Filgotinib works in Crohn’s disease. Furthermore, the mechanism by which these drugs would inhibit the inflammasome has not been properly explained. The novelty of the paper is also partly hampered by data from recent studies showing that JAK inhibition leads to suppression of the inflammasome activation in other diseases (Zhu, Front Immunol 2021; Patel, Annals of Surgery 2023).
Specific points:
1. Although the data seem to indicate that Upadacitinib and Filgotinib, but not Tofacitinib, act as inhibitors of inflammasome activation, no conclusive data is convincingly provided to show the mechanism by which these drugs modulate colitis. Additionally, it is unclear whether the inhibition of Stat molecules somewhat influences the proposed mechanism of action of these drugs.
2. All the data of the present study were generated using a preventive model (drugs were administered starting 3 days before the colitis induction). Since JAK inhibitors are used to treat the active phases of human colitis, the authors should replicate their findings in a therapeutic model by giving the drugs to colitic mice. Moreover, they should confirm their main findings in other models of experimental colitis in which macrophage/dendritic cell activation (e.g. TNBS-colitis) rather than epithelial destruction represents a primum movens in the detrimental mucosal immune response.
3. Criticism is raised on the scheme adopted to treat mice. All the JAK inhibitors were used at the same dose (50 mg/kg body weight, which was administered twice daily via oral gavage), though these compounds are used in patients at different dosages.
4. All the figures must be implemented with data in control, uninflamed mice and the number of mice per single experiment must be increased.
5. Figure 1: Tofacitinib reduces IL-12 and IFN-γ similarly to Filgotinib and Upadacitinib even though Tofacitinib is not able to suppress colitis. The lack of cytokine data in control, uninflamed mice makes it difficult to understand the relevance of Tofacitinib-mediated inhibition of IL-12/IFN-γ production in the gut and whether the benefit seen in mice treated with Filgotinib and Upadacitinib is strictly dependent on the inhibition of IL-6 synthesis. Panels B and C: The differences among groups are not easily appreciable because all the weight curves are superimposed. The authors should represent them more clearly, for example, by separating the data of WT mice from those referring to the model lacking PTPN2 in two graphs. Panel H: data about TNF and other inflammatory cytokines (which are supposed to be pathogenic in the DSS-colitis model) must be provided.
6. Figure 3. Panel B. Labels about treatment are missing. The blot with a loading control is needed to evaluate correctly the results. Panels B-D. There is a discrepancy in the data as panel B shows no effect of Tofacitinib on the mature form of IL-1B in PTPN2-LysMCre while panel D shows that the drug consistently reduces IL-1B secretion, and the lack of statistical

difference between the untreated and Tofacitinib-treated mice could be simply due to the small number of mice analyzed (n=5). Since caspase 1 cleaves pro-IL-18 into the mature form, data about this cytokine must be included.
7. Figure 4. Panel C. Again the blot with a loading control is missing. This representative blot shows also that Tofacitinib reduces the cleavage of caspase 1 and the secretion of mature IL-1B in the variant cells in contrast to what was stated by the authors. The inability of Tofacitinib to inhibit inflammasome activation (induced by NLRP3 activator) is conflicting with results from previous studies showing the inhibitory effect of Tofacitinib on NLRP3 inflammasome activation in other systems (Theranostics 2021 Jan 1;11(3):1446-1457). The inhibitory effect of Upadacitinib on caspase 1 activation and mature IL-1B secretion in both wild-type and variant cells is greater than that shown following Filgotinib treatment but this does not reflect the data shown in panel D.
8. Figure 5: As pointed out above, the lack of data in control, uninflamed mice makes it difficult to interpret such results. However, mice lacking caspase 1 still develop colitis thus indicating that inflammasome activation is not a major driver of gut inflammation in such a model. This data would conflict with the conclusion of the paper that the preventive effect of JAK1 inhibitors, but not Tofacitinib, in DSS-colitis relies on the suppression of inflammasome activation. It would be important to know whether the 3 drugs inhibit JAK activation in caspase 1-deficient mice and what is the role of JAK molecules (particularly JAK1) in the initiation of colitis in such animals.
9. Figure 6: These studies must be complemented by similar experiments using Filgotinib and Tofacitinib. In addition, inflammasome activation should be assessed not only by single-cell RNA sequencing but also at the protein level. It is mandatory to assess additional samples taken from both Crohn's disease patients and Ulcerative colitis patients and ascertain whether there is any difference between these two groups in terms of response to the single drugs. Mucosal expression of IL6, IL-12, and IFN-γ should also be evaluated, and the clinical and demographic characteristics of all patients must be indicated. From the text, it is not clear the type of IBD of these 3 patients. It is also very important to have data on patients' concomitant therapies because steroids are known to inhibit the inflammasome and this could distort the data.
10. Figure 7. Panel A. The mRNA level of NLRP3 after LPS stimulation was reduced by Tofacitinib and not only by Upadacitinib and Filgotinib as stated by the authors. This is in line with previous studies showing that inhibition of Stat3 activation (a target of Tofacitinib) reduces NLRP3 expression (Exp. Neurol. 302, 104–111. 10.1016). Even the secretion of IL-18 in response to NLRP3 activator was consistently reduced by Tofacitinib and the difference (with untreated cells) was not probably significant due to the small number of experiments performed. These issues must be better addressed. Panel D. Although the data show that Upadacitinib and Filgotinib fit into the catalytic site of NLRP3 in a similar way as the known NLRP3 inhibitor MCC950, they are not sufficient to prove that these drugs inhibit directly NLRP3 activation. Further experimentation is needed to assess the inhibitor effect of Upadacitinib and Filgotinib NLRP3 activation in JAK1-deficient cells.

Reviewer #2

(Remarks to the Author)

Relevant topic for the field of IBD and understanding pathways for the novel therapies is timely. A few comments to consider:

General

Level of inflammation needs to be accounted for in the experiments

Tofa treated mice have more inflammation therefore will have more IL1b+ dependent cells and associated pathways?

What efforts have been made to adjust for inflammation rather than treatment driving the outcome

Figure 1 and 5

B and C are unintelligible with 8 overlapping lines, these need to be redone

The severity of colitis in control mice in B and C and in tofa treated mice in Figure 5 C are very different to each other and in Figure 1 very mild

It is not clear how many times in the legend these experiments were performed and in how many mice per group?

Were there sex differences observed?

Figure 2

M1 and M2 macrophages is an outdated term and only relates to in vitro blood not tissue cells

These terms need to be removed and the annotation criteria explained for all cell types

Absolute numbers of cell per cluster?

Cluster allocation by disease

Where are the monocytes? DSS is a model of monocyte recrutiment?

What cell types are used in the DEG in panel C?, should his not be split by cluster to show macrophages are the most affected cell type?

The text terms "leucocytes" but this is actually CD4+ cells and CD11b/CD11c+ cells mixed in a 1:1 ratio

As such interpreting relative abundance in panel F is unhelpful, was flow cytometry analysis done?

Figure 4

There is a clear bi modal distribution for many of the cytokines in Tofa treated WT and variant differentiated monocytes

Why is this? How many independent experiments were performed ?

If on separate days how was this variation accounted for?

Figures 5

A-C are unintelligible and need to be seperated / made clearer

Figure 6

How were these cells annotated?

Can authors report Absolute cells per cluster?

Were cluster allocation split by treatment?

Panel C top of feature plot has been cut off

Have authors done any Pathway analysis on macrophages pre and post treatment? NLRP3/IL1b?

Figure 7

D what controls were used ?

Reviewer #3

(Remarks to the Author)
The current manuscript entitled "JAK1 inhibitors target the NLRP3 inflammasome thereby suppressing inflammatory bowel disease" aims to unravel the molecular mechanism of action of JAK1 inhibitors in IBD. Unfortunately, the manuscript suffers from several major flaws.
- There is no direct evidence using NLPR3 targeting in mice that JAK inhibitors work by controlling NLRP3.
- Observations are limited to a series of colitis prevention studies in a single model. How can these findings predict response to therapy in established IBD inflammation?
- How do the authors explain the lack of effect of tofacitinib on colitis? Can they demonstrate that serum levels of this drug in mice are similar to those in humans on drug therapy?
- Why does tofacitinib induce colitis in WT mice and block colitis in knockout mice (Fig. 1E)?
- Tofacitinib inhibits JAK1. How do the authors explain the lack of efficacy in DSS colitis?
- JAK inhibitors should block effects on multiple immune cell subsets, not just macrophages. How do the authors rule out effects on other immune cell subsets?
- Caspase 1 deficiency should also affect the gasdermin pathway. This needs to be investigated.
- To my knowledge, there is no evidence that IL-1 or IL-18 blockade is effective in human IBD. How can the authors explain that JAK1 inhibitors work by inhibiting these cytokines?

Version 1:

Reviewer comments:

Reviewer #1

(Remarks to the Author)
The authors have revised the manuscript, taking into consideration all my previous comments. In my opinion, the study should be accepted in its current version, as it advances our understanding of the mechanism of action of Jak inhibitors.

Editorial note: Due to the absence of reviewer 2 for this review round this reviewer was additionally asked to comment in place of the missing reviewer:

I have just now had the opportunity to read the paper and see if the authors addressed the points raised by Reviewer 2. In my opinion, they did a great job and all the relevant questions were adequately answered.

Reviewer #3

(Remarks to the Author)
The manuscript examines the different therapeutic mechanisms of various Janus kinase inhibitors in UC and has already been critically reviewed by three reviewers. Despite rejection, the authors have written a rebuttal version, which is now being re-reviewed again.
While tofacitinib and the JAK1-selective inhibitors upadacitinib + filgotinib are all used in the treatment of UC, their different efficacies and exact molecular mechanisms of action are not fully understood. In their study, the authors propose that, in addition to inhibiting the JAK/STAT signaling pathway, upadacitinib and filgotinib uniquely target and suppress the NLRP3 inflammasome, leading to a reduction in IL-1β levels. This dual mechanism of action is considered to be a key factor in their superior efficacy in UC. The study combines in vivo mouse models, in vitro experiments with human cells, and single-cell RNA sequencing data from UC patients.
The authors have made considerable efforts to address the concerns raised by the initial reviewers, thereby significantly improving the manuscript.
good points are:
- The strongest finding of the study lies in the now more convincing evidence that JAK1 inhibitors, unlike tofacitinib, can directly inhibit the NLRP3 inflammasome and subsequent IL-1β production. This finding is novel and provides a plausible explanation for the observed differences in clinical efficacy. Computer-aided modeling (Figure 6E), which shows that these inhibitors fit into the catalytic site of NLRP3 in a manner similar to MCC950, supports this claim with a solid structural basis.
- The authors now provided new siRNA knockdown data on the mechanism of inflammasome inhibition and clarified the direct effect of JAK1 inhibitors on NLRP3 inhibition. (Fig. 6D)
- They now discuss more clearly how their results differ from previous studies and emphasize the direct NLRP3 target effect of JAK1 inhibitors in contrast to the indirect effects reported for other JAK inhibitors.
-The authors now state that they have separated the data from WT and PTPN2-deficient/Casp1-deficient mice for clarity and have now provided details on the number of animals. (Fig. 1, Fig. 5)
- In the updated note, myeloid cells are now better identified as immature and mature macrophages based on Cx3cr1, and has been rerun to explicitly label monocytes. (Fig. 2D) The outdated terminology and cell identification are no longer included.
- The addition of human data from UC patients treated with upadacitinib and the re-analysis of the anti-TNF cohort now provide better evidence for the clinical relevance of their findings.
- The single-cell RNA-Seq data show that IL-1β was reduced more significantly by JAK1 inhibitors compared to other cytokines. This supports the focus on the inflammasome IL-1β signaling pathway as a key distinguishing feature. (suppl. Fig. 4C)

Several points remain still problematic:
-While the in vitro siRNA data and the data from caspase-1-deficient mice strongly suggest direct inhibition of NLRP3, the lack of data from in vivo experiments with Nlrp3-deficient mice remains a limitation. Direct in vivo evidence that the efficacy of JAK1 inhibitors is abolished in Nlrp3-deficient mice would definitively confirm this mechanism in a complex biological system. However, the authors have noted that they unfortunately do not currently have access to the mouse strain.
-Unfortunately, the cohort size for single-cell RNA sequencing is still small. Although these are valuable initial findings, larger multicenter studies that also include other JAK inhibitors (filgotinib and possibly even tofacitinib) would provide greater generalizability and comparable efficacy data in human patients. The recognized confounding factor of prior steroid treatment in some patients with low IL-1β levels also requires further investigation. It is worth considering whether the authors could contribute to this.
-While in-silico modeling suggests direct interaction with the catalytic site of NLRP3 (Figure 6E), direct experimental validation of this binding (e.g., by co-crystallization or detailed biochemical binding assays) would further support this specific mechanistic claim. It is worth considering whether this could be done.
-The authors of this study note that although other immune cells and signaling pathways may also be affected, they claim that macrophages are the primary target for IL-1β production. Further analyses investigating the influence of JAK1 inhibitors on other immune cell subsets and their contribution to the overall efficacy of the therapy could provide a more complete picture here.

This revised manuscript presents a very informative and well-conducted study that provides important insights into the different mechanisms of action of JAK inhibitors in ulcerative colitis. The authors have carefully addressed the reviewers' concerns, in particular by generating new data on the direct NLRP3-inhibiting effect of JAK1 inhibitors, a second experimental model, and the validation of these results using samples from human UC patients. The study is now in better shape. It could represent a valuable contribution to therapeutic options in the field of inflammatory bowel disease.

Reviewer #4

(Remarks to the Author)
The authors have provided a very comprehensive rebuttal to the criticisms of the 3 original reviewers and performed a significant amount of additional work.

Version 2:

Reviewer comments:

Reviewer #3

(Remarks to the Author)
Many thanks to the authors. They have thoroughly addressed my points and I now agree with the manuscript.

Open Access This Peer Review File is licensed under a Creative Commons Attribution 4.0 International License, which permits use, sharing, adaptation, distribution and reproduction in any medium or format, as long as you give appropriate credit to the original author(s) and the source, provide a link to the Creative Commons license, and indicate if changes were made.
In cases where reviewers are anonymous, credit should be given to 'Anonymous Referee' and the source.
The images or other third party material in this Peer Review File are included in the article's Creative Commons license, unless indicated otherwise in a credit line to the material. If material is not included in the article's Creative Commons license and your intended use is not permitted by statutory regulation or exceeds the permitted use, you will need to obtain permission directly from the copyright holder.
To view a copy of this license, visit https://creativecommons.org/licenses/by/4.0/

To reviewers' comments:

We are grateful for the insightful comments of the reviewers as they allowed us to improve our work. In essence, we did the following key experiments: **i)** We confirmed our data in a second colitis model, namely the T cell transfer colitis model; **ii)** as in IBD patients, these drugs were used in different dosages, and as suggested we titrated the drugs in the DSS-colitis model; **iii**) We used *Nlrp3*-deficient macrophages to confirm that the reduction of IL-1β induced by Upadacitinib and Filgotinib is mediated by their action on the NLRP3 inflammasome; **iv)** To assess the effect of Upadacitinib on IL-1β in patients with ulcerative colitis (UC), we performed immunohistochemistry for IL-1β in the colon tissue before and after Upadacitinib treatment. We found that Upadacitinib treatment reduced IL-1β levels and this was associated with a therapeutic response; **v)** To test if the reduction of IL-1β in UC patients is a direct effect of Upadacitinib, rather than an indirect consequence of disease suppression, we included a published cohort where patients were treated with anti-TNF therapy. The results demonstrate that IL-1β expression is unchanged upon anti-TNF treatment.

The detailed reply to reviewers' comments is listed below:

Reviewer #1 (Remarks to the Author):

In this original article, the authors take on the important task of trying to dissect the mechanism of action of JAK inhibitors. In particular, they attempted to verify whether 3 different Jak inhibitors (i.e. Tofacitinib, Upadacitinib, and Filgotinib), which are used to treat human inflammatory bowel diseases, differ in their ability to modulate pathogenic pathways. The authors show that Upadacitinib and Filgotinib but not Tofacitinib inhibit the inflammasome activation and prevent experimental colitis induced by giving mice oral dextran sulfate sodium, a sugar that causes gut epithelial cell injury.
The data presented in this paper are largely incomplete and leave many open questions.

We are grateful for this critical feedback. We have completed the data as suggested and we have answered most of the open questions as outlined below.

The paper seems to show no difference between Filgotinib and Upadacitinib in terms of inflammasome inhibition and colitis prevention, but the response and remission rates of these two compounds in IBD patients are largely different, and Upadacitinib but not Filgotinib works in Crohn's disease.

We appreciate this point. However, we did not find much difference between Filgotinib- and Upadacitinib-treated mice on colitis phenotype nor gene expression level (Sup. Fig 4B, attached below), particularly in terms of inflammasome inhibition. Of note, we used DSS colitis, which is similar to Ulcerative Colitis (UC) in humans, and indeed both compounds are approved in UC. We agree with the reviewer that Upadacitinib but not Filgotinib is approved for Crohn's disease (CD), and this may be due to different study designs and dosing regimens in the respective trials. However, there are no head-to-head studies between these two drugs for either UC or CD. Therefore, the statement of the reviewer "**that the response and remission rates of these two compounds in IBD patients are largely different**" is based on network analysis and indirect comparisons. While such analyses provide valuable insight, this statement would benefit from further validation of the direct head-to-head studies. In any case, the aim of our study was not to compare the response rate of these drugs and we believe that answering this question in humans is outside the scope of this work.

Sup.Fig.4B

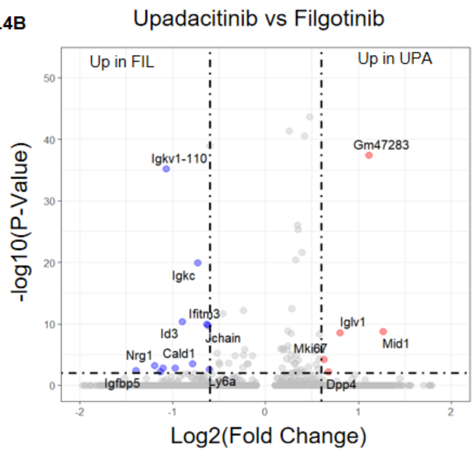

Supplementary Figure 4B. Differentially expressed genes (DEGs) between Upadacitinib and Filgotinib in single-cell RNA sequencing analysis of murine colonic immune cells.

Furthermore, the mechanism by which these drugs would inhibit the inflammasome has not been properly explained.

In the original submission we performed a structural overlay analysis indicating that Upadacitinib and Filgotinib bind to the same pocket of NLRP3 as MCC950, a known NLRP3 inhibitor. However, based on this comment, we now demonstrate the mechanism by which these drugs would inhibit the inflammasome, namely by direct binding to the NLRP3 Inflammasome. We knocked down the NLRP3 in the human

macrophages and observed that JAK1 inhibitors showed a much-compromised inhibitory function on IL-1β compared to scramble control. Additionally, to further test whether inhibition of the JAK1 pathway also contributes to the regulation of IL-1β by JAK1 inhibitors, we tested the effect of the drugs in *JAK1*-deficient macrophages (Figure 6D). The results revealed that the suppression of IL-1β is independent on JAK1. In contrast, Tofacitinib did not show any ability to inhibit IL-1β regardless of the presence of JAK1 and NLRP3. Therefore, these additional data provide new insights explaining the mechanism of the action of JAK1 inhibitors.

Fig. 6D

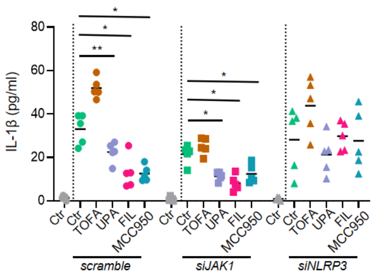

Figure 6D. Macrophages from healthy controls (n=5) were transfected by JAK1, NLRP3, or scramble control siRNA respectively for 48 h, then treated with LPS alone, or LPS with different JAK inhibitors or MCC950 respectively for 10h. Cell culture supernatant was assessed for IL-1β by ELISA.

The novelty of the paper is also partly hampered by data from recent studies showing that JAK inhibition leads to suppression of the inflammasome activation in other diseases (Zhu, Front Immunol 2021; Patel, Annals of Surgery 2023).

We thank the reviewer for raising this point

We strongly believe that our data are indeed novel as we show that JAK1 inhibitors act directly on the NLRP3 inflammasome to suppress IL-1β activation. More importantly, as outlined in detail below, we show its relevancy in treating patients with UC, namely the reduction of IL-1β level upon Upadacitinib treatment is associated with disease remission in UC patients.

We appreciated the comments from the reviewer about the previous studies. We have now included a detailed discussion regarding this point. '*In a previous study Zhu et al. showed Ruxolitinib, a JAK1/2 inhibitor, can suppress NLRP3 indirectly via inhibiting STAT3 in a murine cerebral ischemic stroke model. On the contrary, our data indicate*

that Upadacitinib and Filgotinib act as direct NLRP3 inhibitors independently of STAT3 signaling (Reply Figure 1, shown below in response to Comment#1 on Page 9). Specifically, we tested whether the JAK1 pathway regulates the NLRP3-IL-1β axis by knocking down JAK1 and NLRP3 in human macrophages. We observed that JAK1 inhibitors can still efficiently reduce the production of IL-1β in JAK1-deficient but not in NLRP3-deficient macrophages. Furthermore, Patel et al. also reported a JAK1/2 inhibitor, Baricitinib, inhibited NLRP3-Caspase 1 axis, but the underlying mechanism was not identified. Therefore, it appears that several JAK inhibitors can interact directly and indirectly with the inflammasome' (Line: #421-429)

Specific points:

1. Although the data seem to indicate that Upadacitinib and Filgotinib, but not Tofacitinib, act as inhibitors of inflammasome activation, no conclusive data is convincingly provided to show the mechanism by which these drugs modulate colitis. Additionally, it is unclear whether the inhibition of Stat molecules somewhat influences the proposed mechanism of action of these drugs.

We now provide an amplitude of conclusive data showing that inhibition of the NLRP3 inflammasome contributes to the efficacy of Upadacitinib and Filgotinib in colitis.

First, to address the mechanism by which Upadacitinib and Filgotinib (JAK1 inhibitors) exert their action during colitis, we performed single cell RNA sequencing in mice treated with JAK1 inhibitors and Tofacitinib (pan JAK inhibitor) during colitis. We found JAK1 inhibitors, but not Tofacitinib downregulated genes related to IL-1 signaling, especially *Nlrp3* and *IL1b*, which indicated that NLRP3 inflammasome pathway might play an important role in JAK1 inhibitor efficacy (Fig. 2B and C).

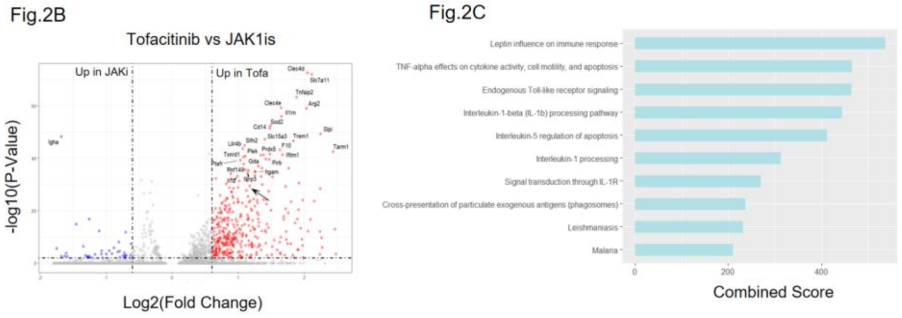

Figure 2B and 2C. **B)** DEGs between the Tofacitinib-treated group and JAK1i group (including Upadacitinib and Filgotinib) in single-cell RNA sequencing analysis of murine colonic immune cells; C) pathway analysis based on the upregulated DEGs in tofacitinib versus JAK1i group.

Second, to further investigate a potential involvement of the NLRP3 inflammasome in the observed effects, we assessed IL-1β as well as Caspase1 which is the molecule downstream of NLRP3 to activate IL-1β, and found IL-1β was decreased by JAK1 inhibitors but not Tofacitinib (Fig. 3).

Fig.3

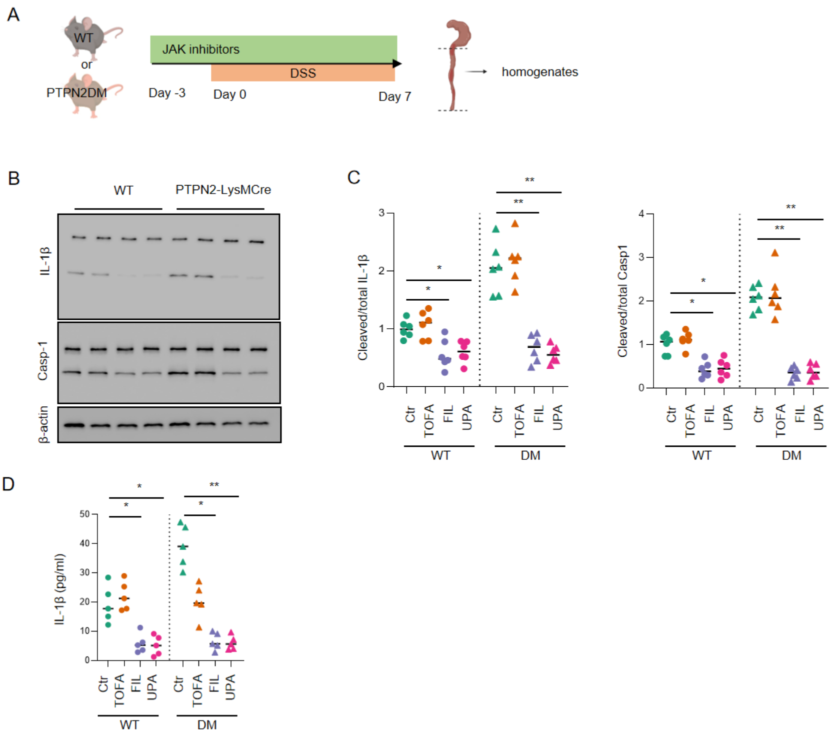

Figure 3. Filgotinib and Upadacitinib inhibit inflammasome activation in mouse colon. PTPN2-LysMCre (PTPN2-ΔM) mice and their WT littermates were orally gavaged with the JAK inhibitors Tofacitinib, Filgotinib, Upadacitinib starting three days prior to administration of 2% DSS in the drinking water for 7 days. At the end of the experiment, colons were homogenized and analyzed for IL-1β and Caspase-1 cleavage by Western blot. **A)** Experimental set-up; **B)** representative pictures and **C)** densitometric analysis of Western blots for the indicated proteins; **D)** IL-1β level in the supernatant of colon pieces obtained on the last day of the experiment and incubated for 24h in cell culture medium. *p<0.05, **p<0.01, ANOVA with Holm-Sidak's multiple comparison test.

Third, we used *Caspase1*-deficient mice to prove that the higher efficacy of JAK1 inhibitors compared to Tofacitinib is dependent on inflammasome pathway (Figure 5E

and G).

Fig.5E

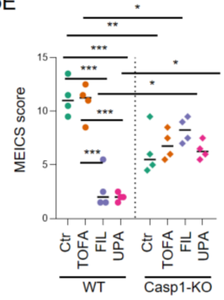

Fig.5G

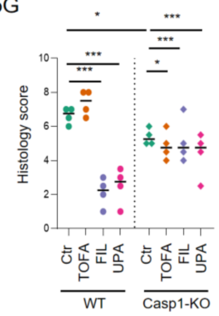

Figure 5E and 5G. The protective effect of Filgotinib and Upadacitinib is abrogated in Caspase1-deficient mice. **E)** respective scoring of mouse endoscopy on the last day of the experiment; **G)** scoring for disease severity of H&E-stained sections of the terminal colon. *p<0.05, **p<0.01, ***p<0.001, ANOVA with Holm-Sidak's multiple comparison test.

Finally, we analyzed bio-samples from UC patients before and after treatment with Upadacitinib. We observed a reduction of IL-1β, which correlated with the response to Upadacitinib therapy (Figure 7C and F). As control, we analyzed a cohort of patients with UC before and after anti-TNF therapy. Here, we found no reduction in IL-1β indicating that the observed effect is not merely mediated by a reduced inflammatory activity (Sup.Fig.8).

Fig.7C

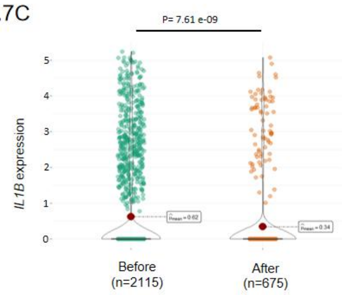

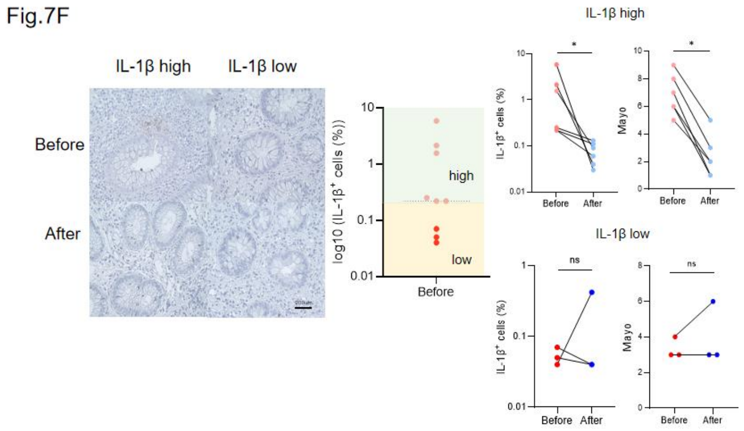

Figure 7C and 7F. Upadacitinib downregulates *NLRP3* and *IL1B* expression in UC patients. Immunocytes isolated from lamina propria of IBD patients treated with Upadacitinib were analyzed by single- cell RNA sequencing. **C)** Violin Plot of IL1B expression before and after Upadacitinib treatment. P-value was adjusted by Holm-Bonferroni method. **F)** IHC of IL-1β in sigmoid of Upadacitinib. Left: representative pictures showing IL-1β expression (brown). Middle: quantified IL-1β expression before Upadacitinib treatment (baseline). Patients were grouped to IL-1β high or low group based on the median of IL-1β expression among all individuals. Dotted line represents the median value. Right upper lane: quantified IL-1β expression and Mayo score of the patients in “IL-1β high” group before and after Upadacitinib treatment. Right lower lane: quantified IL-1β expression and Mayo score of the patients in “IL-1β low” group before and after Upadacitinib treatment. Significance was analyzed using Wilcoxon test, *$p<0.05$.

Sup. Fig. 8

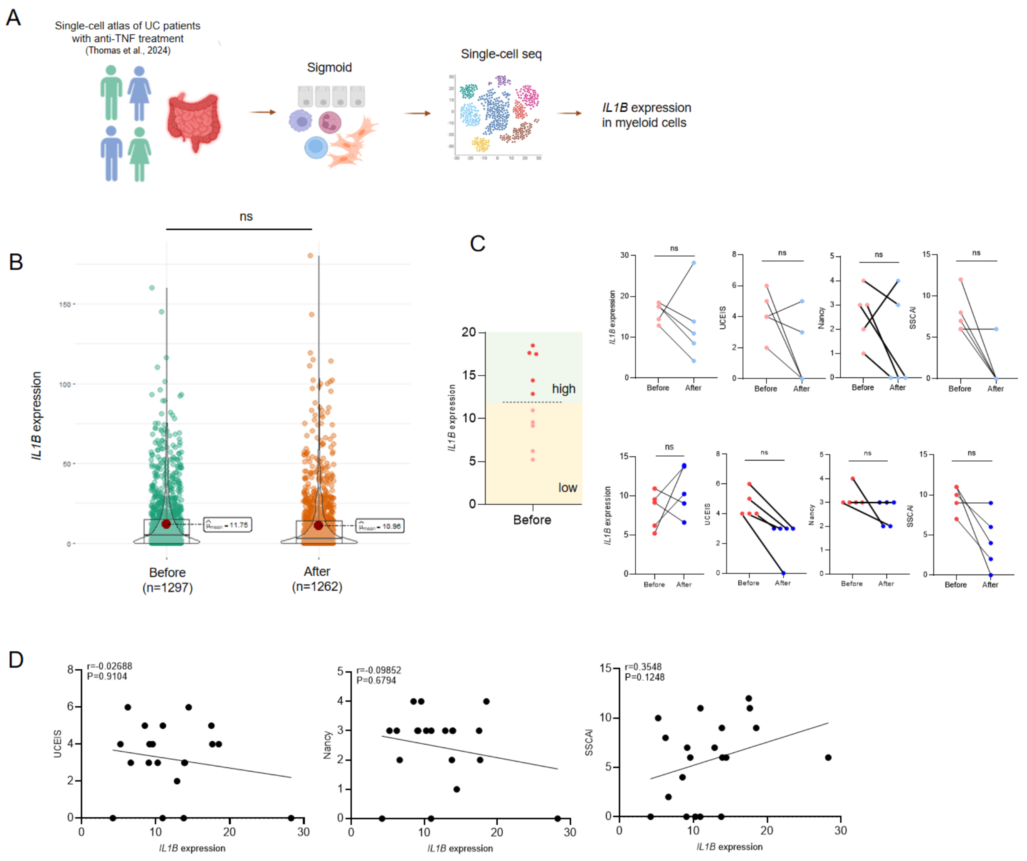

Supplementary Figure 8. *IL1B* expression in UC patients with anti-TNF treatment. *IL1B* expression was assessed in myeloid cells from the single-cell atlas (Thomas et al., 2024) with 10 UC patients before and after Adalimumab. **A)** Analysis setup; **B)** Violin Plot of *IL1B* expression in patients before and after Adalimumab treatment. P-value was adjusted by Holm-Bonferroni method; **C)** *IL1B* expression and colitis assessment in the individual UC patient. Left: *IL1B* expression of patients before Adalimumab treatment. Right upper lane: Patients with high *IL1B* expression and their clinical colitis evaluation. Right lower lane: Patients with low *IL1B* expression and their clinical colitis evaluation. UCEIS, Ulcerative Colitis Endoscopic Index of Severity; Nancy, histopathological Nancy index; SSCAI, Simple Clinical Colitis Activity Index. P-value was determined by Wilcoxon matched-pairs signed rank test. **D)** Correlation of *IL1B* expression with colitis scores in Adalimumab treatment. Correlation was tested by nonparametric Spearman correlation.

We hope with all the results, we clearly demonstrated our conclusion and answered the question from the reviewer.

For the comment '**Additionally, it is unclear whether the inhibition of Stat molecules somewhat influences the proposed mechanism of action of these drugs.**', we tested the effect of different JAK inhibitors on phosphorylated STAT1 and STAT3. We found Tofacitinib reduces phosphorylation of STAT1 and STAT3, while Upadacitinib and Filgotinib could only reduce STAT1 (Reply Fig.1). However, Tofacitinib was not able to down-regulate IL-1β, suggesting that our proposed mechanism of action, which is the inflammasome-IL-1β axis, is not associated with STAT1 or STAT3. To further strengthen this point, we knocked down *JAK1* in human macrophages using siRNA to test if JAK1 inhibitors would be able to work as NLRP3 inhibitor even in the absence of JAK1. And the result revealed JAK1 inhibitors indeed reduced IL-1β production in *JAK1*-deficient macrophages similarly as NLRP3 inhibitor MCC950, suggesting that the reduction of IL-β expression induced by JAK1 inhibitors is independent of JAK1 pathway (Fig. 6D). However, with knocking down *NLRP3*, the suppressive capacity of JAK1 inhibitors on IL-1β is largely reduced. Tofacitinib did not suppress IL-1β in any of the conditions. Taken together, in our study, the action of JAK1 inhibitors on NLRP3 inflammasome- IL-1β axis is not much affected by JAK/STAT pathway.

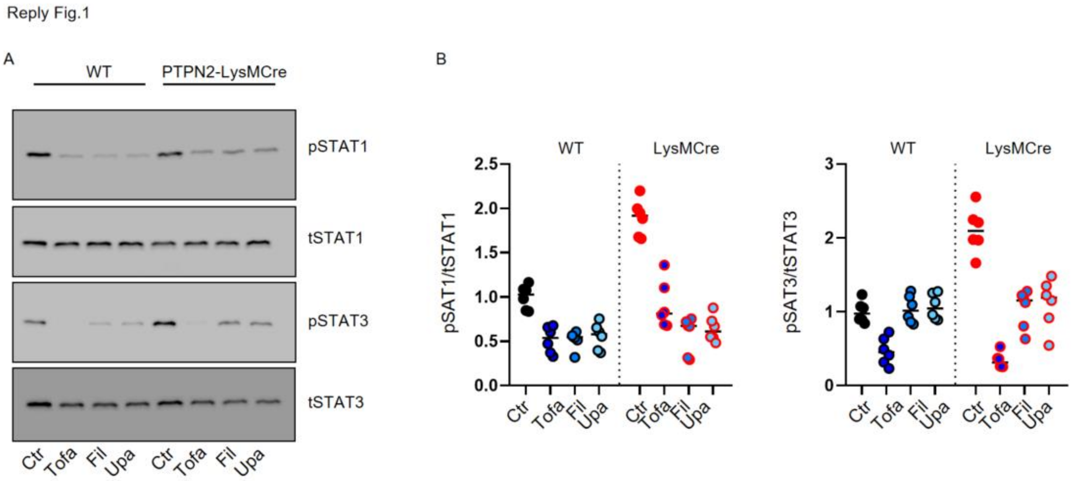

Reply Figure 1. Phosphorylation of STAT1 and STAT3 upon the treatment of JAK inhibitors in mouse colon lysates after DSS administration. **A)** representative pictures and **B)** densitometric analysis of Western blots for the indicated proteins.

Fig. 6D

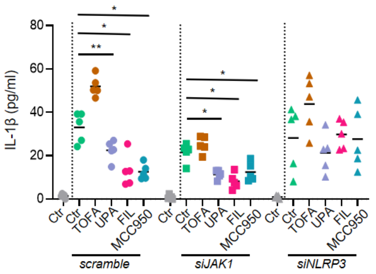

Figure 6D. Macrophages from healthy controls (n=5) were transfected by JAK1, NLRP3, or scramble control siRNA respectively for 48 h, then treated with LPS alone, or LPS with different JAK inhibitors or MCC950 respectively for 10h. Cell culture supernatant was assessed for IL-1β by ELISA.

2. All the data of the present study were generated using a preventive model (drugs were administered starting 3 days before the colitis induction). Since JAK inhibitors are used to treat the active phases of human colitis, the authors should replicate their findings in a therapeutic model by giving the drugs to colitic mice.

We agree that this is a valid point. However, the ideal situation to study this, is actually with human IBD patients which are treated with the JAK1 inhibitor in a therapeutic setting. To this end, we obtained intestinal biopsy specimen before and upon Upadacitinib treatment. We found Upadacitinib indeed reduced IL-1β expression in UC patients. Furthermore, we observed that the decreased IL-1β expression is associated with response to Upadacitinib therapy (Fig.7C and F, shown above in response to Comment #1). Additionally, as a control, we included a published cohort where the patients were treated with anti-TNF therapy (Adalimumab). We observed that Adalimumab improved the inflammation in UC patients without changing IL-1β expression suggesting that the effect of Upadacitinib on the NLRP3-IL-1β axis is not merely due to a reduced disease activity (Sup.Fig.8, shown above in response to Comment #1). We believe that these human data are more relevant than further murine data. However, we would be willing to assess the therapeutic effect in the transfer colitis model, if deemed essential.

Moreover, they should confirm their main findings in other models of experimental colitis in which macrophage/dendritic cell activation (e.g. TNBS-

colitis) rather than epithelial destruction represents a primum movens in the detrimental mucosal immune response.

We appreciated the reviewer for raising this point. However, we respectfully disagree with the perspective. Actually, DSS-induced colitis is an innate-driven model (Berndt et al., *J Immunol*, 2007; Kiesler et al., *Cell Mol Gastroenterol Hepatol*, 2015). In this model, innate cells, including macrophages and dendritic cells, are activated by the microorganisms in the gut due to epithelial barrier defects. Despite of this, we still assessed the effect of the JAK inhibitors in an additional experimental colitis model. We employed the T cell transfer colitis model in which immune cell activation also represents a primum movens in the detrimental mucosal immune response. In this model, we also found a higher efficacy of Upadacitinib and Filgotinib compared to Tofacitinib (Sup.Fig.1).

Sup.Fig.1

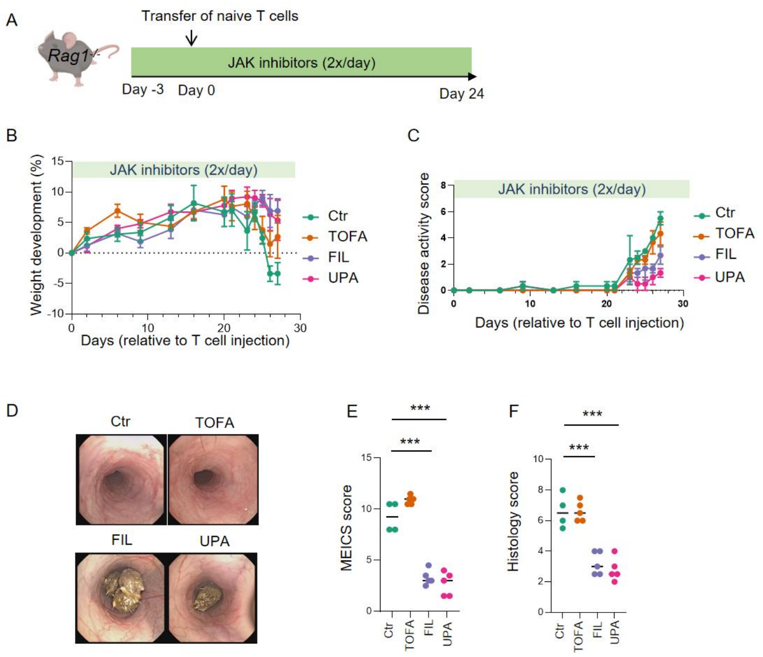

Supplementary Figure 1. Upadacitinib and Filgotinib reduce T cell mediated colitis. *Rag1*$^{-/-}$ mice were orally gavaged with the JAK inhibitors Tofacitinib, Filgotinib, Upadacitinib starting three days prior of the injection of 0.25x10^{6} naïve T cells. **A)** Experimental set-up; **B)** weight development; **C)** disease activity score over time; **D)** representative images of colitis and **E)** respective scoring of mouse endoscopy on the last day of the experiment. ***p<0.001, ANOVA with Holm-Sidak's multiple comparison test.

3. Criticism is raised on the scheme adopted to treat mice. All the JAK inhibitors were used at the same dose (50 mg/kg body weight, which was administered twice daily via oral gavage), though these compounds are used in patients at different dosages.

In ulcerative colitis, the recommended dose for induction treatment is 10mg twice a day, 45 mg once daily and 200 mg once daily for Tofacitinib, Upadacitinib and Filgotinib, respectively. In light of this comment, we performed a drug titration experiment. We observed that 50 mg/kg body weight, which was administered twice daily, was the most efficient for Upadacitinib and Filgotinib compared to 5.6mg/kg and 17mg/kg. However, treatment with 50mg/kg body weight Tofacitinib or even higher concentration (150mg/kg and 450mg/kg) had no effect on colitis severity in WT mice (Sup.Fig.2). Therefore, our data indicate that even higher doses of Tofacitinib are not as efficient as lower doses of JAK1 inhibitors indicating that the mode of action rather than the dose is critical. We do discuss this point now in the manuscript: '*Considering these drugs are used in UC treatment at different dosages (10mg twice a day, 45 mg once daily and 200 mg once daily for Tofacitinib, Upadacitinib and Filgotinib respectively), we next tested in a drug titration experiment whether the different efficacy between Tofacitinib and JAK1i is dependent on the dosage. We observed that even in the highest concentration (450mg/kg), Tofacitinib was not as efficacious as JAK1i in suppressing colitis susceptibility in mice, indicating that the dosage is less critical in this setting.*' (Line: #323-329). These data further support the notion that the mode of action rather than the dosage explains the different efficacy of JAK1 inhibitors compared to Tofacitinib.

Sup.Fig.2

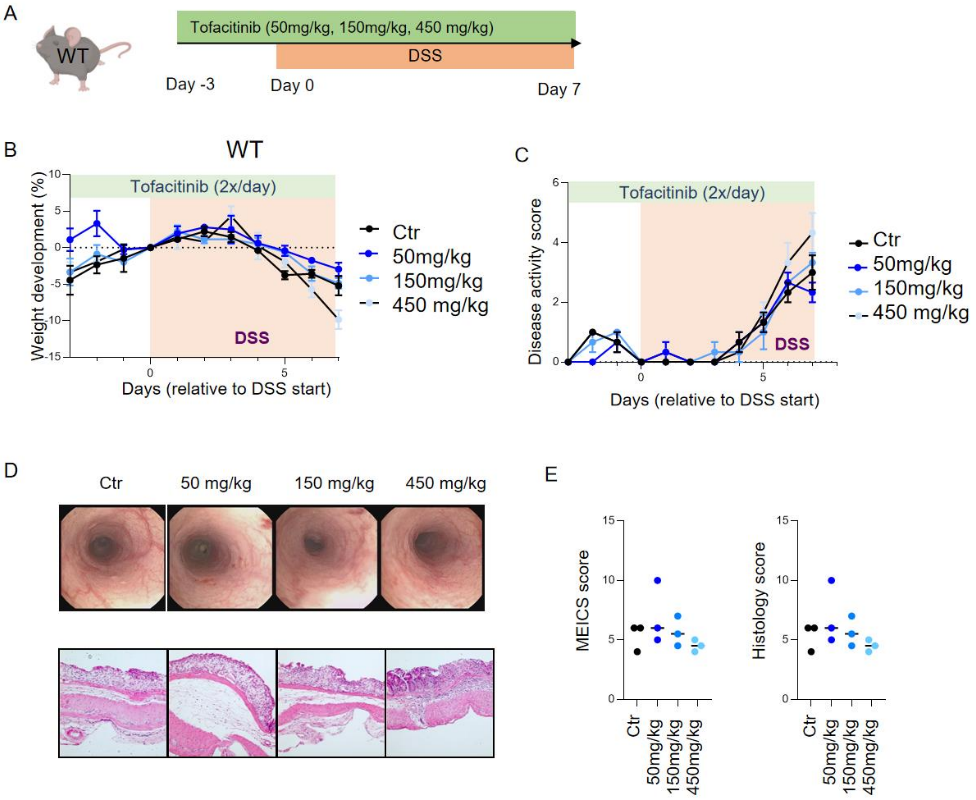

Supplementary Figure 2. High doses of Tofacitinib are not effective in reducing colitis severity. WT mice were orally gavaged with the indicated doses of JAK inhibitor Tofacitinib starting three days prior to administration of 2% DSS in the drinking water for 7 days. **A)** Experimental set-up; **B)** weight development; **C)** disease activity score over time; **D)** representative images of and **E)** respective scoring of mouse endoscopy (top) and H&E-stained sections of the terminal colon (bottom) on the last day of the experiment.

4. All the figures must be implemented with data in control, uninflamed mice and the number of mice per single experiment must be increased.

We believe that our study is significantly powered to answer the proposed questions. Due to the strict animal welfare legislation in Switzerland and Germany, increasing the number of mice beyond what is already included is not possible, because it would subject animals to unnecessary suffering.

5.1 Figure 1: Tofacitinib reduces IL-12 and IFN-γ similarly to Filgotinib and Upadacitinib even though Tofacitinib is not able to suppress colitis. The lack of cytokine data in control, uninflamed mice makes it difficult to understand the

relevance of Tofacitinib-mediated inhibition of IL-12/IFN-y production in the gut and whether the benefit seen in mice treated with Filgotinib and Upadacitinib is strictly dependent on the inhibition of IL-6 synthesis.

Uninflamed mice or mice in steady state do not show much cytokine production. Therefore, it is difficult to show the function of JAK inhibitors in general in non-inflamed mice.

Upon colitis induction, we found similar strength from JAK1 inhibitors and Tofacitinib to decrease IL-12 and IFN-γ production (Fig. 1H), but Tofacitinib could not protect mice from colitis, suggesting that the efficacy is not mainly mediated by the effects of IL-12 and IFN-γ. The effect on IL-6 showed a difference between JAK1 inhibitors and Tofacitinib. However, these mice also showed a different disease severity. To control for disease severity, we analyzed RNA sequencing data from immune cells isolated from mice treated with either JAK1 inhibitors or tofacitinib, selecting animals with comparable disease scores. Under these controlled conditions, the inflammasome and IL-1 signaling pathways emerged as the most differentially expressed between the two treatment groups. (Fig.2B and C, shown above in response to Comment#1). Additionally, comparison of the average expression of cytokines across different treatments revealed that *Il1b* was more markedly reduced by JAK1 inhibitors than the other cytokines, including IL-6 (Sup. Fig. 4C). Therefore, we focused the investigation on the inflammasome-IL-1β pathway. Nevertheless, we acknowledge the possibility that other cytokines and pathways contribute to the overall therapeutic effects of JAK inhibitors in IBD treatment and warrant further investigation.

Sup.Fig.4C

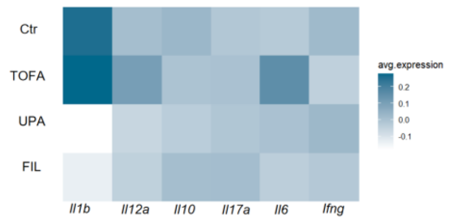

Supplementary Figure 4C. Single-cell RNA sequencing analysis of murine colonic immune cells. CD4+ T cells and CD11b+ and/or CD11c+ cells were isolated and FACS sorted from mouse colon samples and merged in a 1:1 ratio. Gene expression was examined by single-cell analysis. Average expression of the indicated genes among groups was shown in the heatmap.

Next, to demonstrate the role of inflammasome pathway in the efficacy of JAK inhibitors, we furthermore used Caspase1-deficient mice to support our conclusion that the inhibition of the inflammasome contributes to JAK1 efficacy (Fig.5E and G, shown above in response to Comment#1).

Overall, in light of this comment we do discuss these points in our manuscript: '*Notably, in the mouse colitis model, we also observed a reduction in several other pro-inflammatory cytokines—such as IL-12, and IFN-γ. However, both Tofacitinib and the JAK1 inhibitors reduced these cytokines to comparable levels, suggesting that they are unlikely to be the primary drivers of the different therapeutic efficacy observed. This interpretation is further supported by our single-cell analysis, which did not implicate these cytokines as key factors underlying the distinct responses to the treatments. Collectively, our findings indicate that inhibition of the NLRP3–IL-1β axis plays a critical role in mediating the superior efficacy of Upadacitinib and Filgotinib compared to Tofacitinib in the context of ulcerative colitis. Nonetheless, our data do not exclude the possibility that other cytokines, including IL-6, IL-12, and IFN-γ, and other pathways (Figure 2C) may also contribute. Further studies will be necessary to elucidate the specific functional roles of these cytokines and pathways in modulating treatment responses.'* (Line: #372-383)

Fig.1H

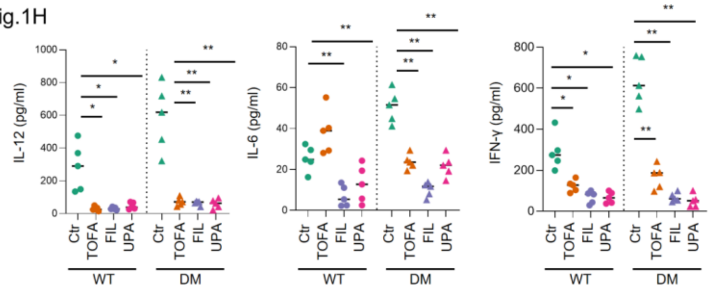

Figure 1H. Levels of the indicated cytokines from the supernatant of colon explants. Colon pieces were obtained on the last day (Day7) of the DSS model and incubated for 24 h in cell culture medium. *p<0.05, **p<0.01, ANOVA with Holm-Sidak's multiple comparison test.

5.2 Panels B and C: The differences among groups are not easily appreciable because all the weight curves are superimposed. The authors should represent them more clearly, for example, by separating the data of WT mice from those referring to the model lacking PTPN2 in two graphs. Panel H: data about TNF and other inflammatory cytokines (which are supposed to be pathogenic in the DSS-colitis model) must be provided.

As suggested, we separated the WT mice from PTPN2-deficient mice in Fig.1 B and C. Also, we provide data about TNF and IL-17A (which are supposed to be pathogenic in the DSS-colitis model) (Reply Fig.2). They did not show much difference between the therapies.

Reply Fig. 2

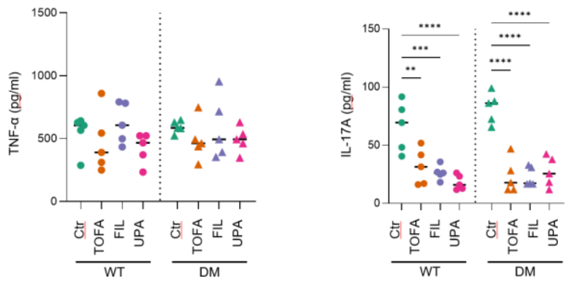

Reply Figure 2. PTPN2-LysMCre (PTPN2-ΔM) mice and their WT littermates were orally gavaged with the JAK inhibitors Tofacitinib, Filgotinib, Upadacitinib starting three days prior to administration of 2% DSS in the drinking water for 7 days. Levels of the TNF-α and IL-17A in the supernatant from colon explant. Colon pieces were obtained on the last day of the experiment and incubated for 24 h in cell culture medium. **p<0.01, ***p<0.001, ***p<0.0001, ANOVA with Holm-Sidak's multiple comparison test.

6. Figure 3. Panel B. Labels about treatment are missing. The blot with a loading control is needed to evaluate correctly the results. Panels B-D. There is a discrepancy in the data as panel B shows no effect of Tofacitinib on the mature form of IL-1B in PTPN2-LysMCre while panel D shows that the drug consistently reduces IL-1B secretion, and the lack of statistical difference between the untreated and Tofacitinib-treated mice could be simply due to the small number of mice analyzed (n=5). Since caspase 1 cleaves pro-IL-18 into the mature form, data about this cytokine must be included.

Labels about treatment are now included in panel B. The blot with a loading control is now also included.

We have reported previously (Marianne et al., Cell Rep., 2018) that mice lacking PTPN2 have enhanced IL-1β production. In Panel D, we found that in PTPN2-deficient mice, Tofacitinib decreased IL-1β, but only to the level of their WT controls. The reviewer is correct that there is an inconsistency between Panel C and D, we have discussed it now in the manuscript '…*we found reduced levels of cleaved (active) Caspase-1 and IL-1β in lysates from Upadacitinib- and Filgotinib-treated mice compared to untreated control mice using western blot (WB). But there were no*

significant differences in lysates from Tofacitinib-treated mice compared to control. We observed by trend a decrease in IL-1β expression by Tofacitinib from PTPN2-deficient mice using ELISA (Figure 3D). This may be due to a technical sensitivity compared to WB. However, the reduction of IL-1β was in any case much lower compared to Upadacitinib and Filgotinib.' (Line: #345-348)

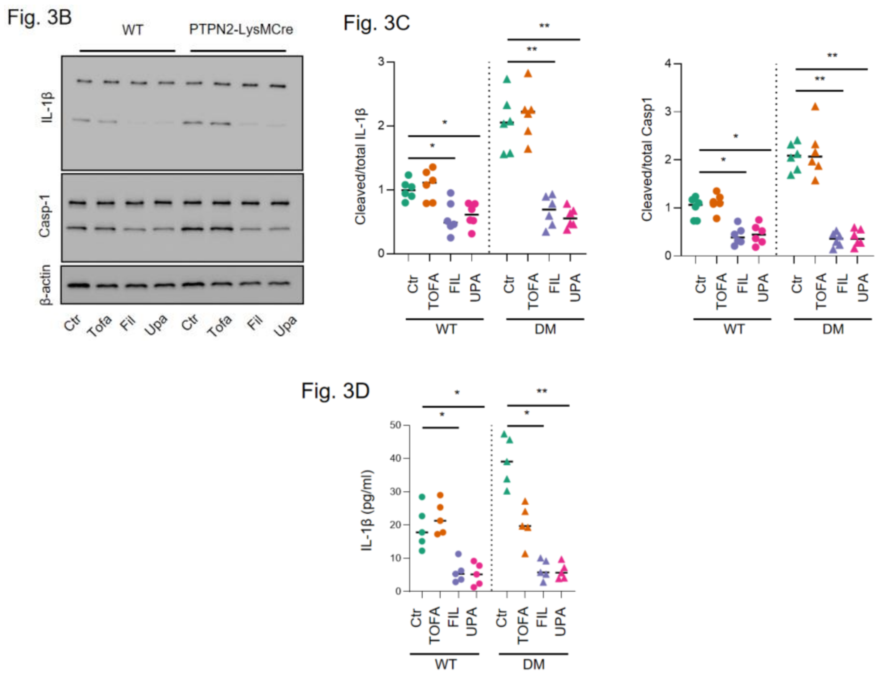

Figure 3B-D. Filgotinib and Upadacitinib inhibit inflammasome activation in mouse colon. PTPN2-LysMCre (PTPN2-ΔM) mice and their WT littermates were orally gavaged with the JAK inhibitors Tofacitinib, Filgotinib, Upadacitinib starting three days prior to administration of 2% DSS in the drinking water for 7 days. At the end of the experiment, colons were homogenized and analyzed for IL-1β and Caspase-1 cleavage by Western blot. **B)** representative pictures and **C)** densitometric analysis of Western blots for the indicated proteins;**D)** IL-1β level in the supernatant of colon pieces obtained on the last day of the experiment and incubated for 24h in cell culture medium. *p<0.05, **p<0.01, ANOVA with Holm-Sidak's multiple comparison test.

Since caspase 1 cleaves pro-IL-18 into the mature form, we included data about this cytokine in Figure 3 as well. Similar to IL-1β, JAK1 inhibitors reduced IL-18 level in both WT and PTPN2-deficient mice upon colitis, while Tofacitinib did not.

Fig. 3E

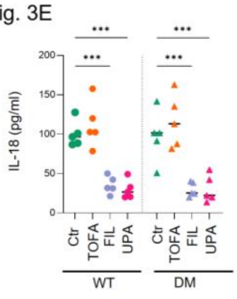

Figure 3E. Filgotinib and Upadacitinib inhibit inflammasome activation in mouse colon. PTPN2-LysMCre (PTPN2-ΔM) mice and their WT littermates were orally gavaged with the JAK inhibitors Tofacitinib, Filgotinib, Upadacitinib starting three days prior to administration of 2% DSS in the drinking water for 7 days. E) IL-18 level in the supernatant of colon pieces obtained on the last day of the experiment and incubated for 24h in cell culture medium. *p<0.05, **p<0.01, ***p<0.001, ANOVA with Holm-Sidak's multiple comparison test.

7. Figure 4. Panel C. Again the blot with a loading control is missing. This representative blot shows also that Tofacitinib reduces the cleavage of caspase 1 and the secretion of mature IL-1B in the variant cells in contrast to what was stated by the authors. The inability of Tofacitinib to inhibit inflammasome activation (induced by NLRP3 activator) is conflicting with results from previous studies showing the inhibitory effect of Tofacitinib on NLRP3 inflammasome activation in other systems (Theranostics 2021 Jan 1;11(3):1446-1457). The inhibitory effect of Upadacitinib on caspase 1 activation and mature IL-1B secretion in both wild-type and variant cells is greater than that shown following Filgotinib treatment but this does not reflect the data shown in panel D.

We added the loading controls as suggested.

We appreciated the reviewer's comments. However, the total quantification of the WB from Tofacitinib-treated cells did not show a significant difference compared to control (Fig. 4D). We observed the reduction of IL-1β in some samples treated by Tofacitinib (Fig. 4C), which is in line with the previous study mentioned by the reviewer. However, when we quantified and assessed all samples, there was no significant difference. In contrast, Upadacitinib and Filgotinib had quantitative significance on IL-1β reduction. As for the discrepancy between Upadacitinib and Filgotinib: In some samples Upadacitinib showed a higher suppression of Caspase-1 and IL-1β than Filgotinib (Fig. 4C). However, there is no significant difference between these two JAK1 inhibitors, when quantifying and comparing all samples (Fig. 4D).

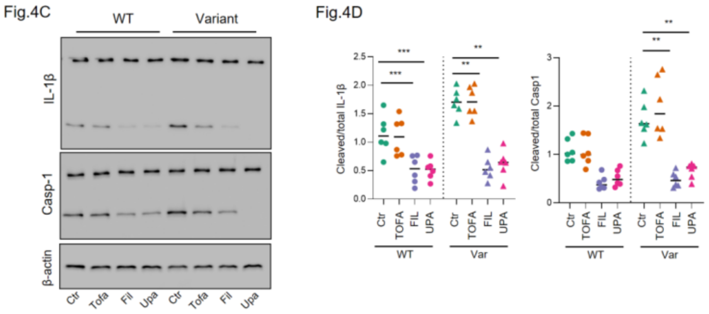

Figure 4C and D. Upadacitinib and Filgotinib inhibit inflammasome activation in human cells. Monocytes were isolated from the peripheral blood of IBD patients that were either WT or variant for the loss-of-function SNP rs1893217 in the gene locus encoding *PTPN2.* These cells were differentiated into macrophages and then treated for 24h with LPS prior to activation with MSU crystals (150 ng/ml) for 6h. **C)** Representative Western blot pictures and **D)** densitometry for the indicated proteins. *p<0.05, **p<0.01, ANOVA with Holm-Sidak's multiple comparison test.

We furthermore discuss now the study mentioned by the reviewer in the revised manuscript: '*Yang et al. (Yang et al., Theranostics, 2021) investigated the effect of Tofacitinib in a rheumatoid arthritis (RA) model where Tofacitinib was given to mice with RA or to BMDM culture at the dosage of 120ng/day, or at the concentration of 1.5, or 25 μM, respectively. They observed that Tofacitinib protected mice from RA, and inhibited NLRP3 and IL-1β in vivo and in vitro in macrophages, which seems to be contradictory to our results. However,* this study did not investigate the mechanism of how Tofacitinib inhibited NLRP3. *In our study, we tested the efficacy of Tofacitinib in colitis models and here Tofacitinib did not have a protective effect on colitis regardless of the dosages used, which is also in line with a previous study (Spalinger et al, 2021; De Vries et al, 2019). Differences in the disease model used may account for the observed effects of Tofacitinib. In addition, we did observe Tofacitinib reduced the RNA levels of NLRP3 in vitro to some extent. However, when assessing IL-1β production, this had only a minor effect. Thus, it seems that lowering NLRP3 RNA level alone is not the primary determinant of inflammasome activity in this context. In contrast to Tofacitinib, JAK1 inhibitors displayed a more pronounced suppressive effect on IL-1β production, likely associated with their direct impact on NLRP3 inflammasome activation rather than only affecting the transcriptional regulation. Additionally, there was an effect of Tofacitinib on IL-18 release (although not significant, probably due to a low sample size), however, again, this effect was clearly less pronounced than the*

effect of Upadacitinib and Filgotinib, which reinforced our conclusion that the functional inhibition of NLRP3 seems to be more important in modulating IL-1β production than merely reducing NLRP3 transcript level. Of note, though we did not observe a direct effect of Tofacitinib on NLRP3 activation, we cannot rule out indirect effects at different doses similar to what has been described for other JAK inhibitors (Zhu et al., Front Immunol; Patel et al., Annals of Surgery 2023).' (Line: #351-371)

8. Figure 5: As pointed out above, the lack of data in control, uninflamed mice makes it difficult to interpret such results. However, mice lacking caspase 1 still develop colitis thus indicating that inflammasome activation is not a major driver of gut inflammation in such a model. This data would conflict with the conclusion of the paper that the preventive effect of JAK1 inhibitors, but not Tofacitinib, in DSS-colitis relies on the suppression of inflammasome activation. It would be important to know whether the 3 drugs inhibit JAK activation in caspase 1-deficient mice and what is the role of JAK molecules (particularly JAK1) in the initiation of colitis in such animals.

The reviewer is right that mice lacking caspase 1 still develop colitis. These data indicate that inflammasome activation is not essential for colitis development in this model. However, mice lacking caspase 1 developed significantly milder colitis compared to littermate controls indicating that the inflammasome contributes to colitis development in this model. Therefore, these data do not conflict with the conclusion of our paper that the protective effect of JAK1 inhibitors, but not Tofacitinib, in DSS-colitis relies on the suppression of inflammasome activation.

In terms of the comments "**It would be important to know whether the 3 drugs inhibit JAK activation in caspase 1-deficient mice and what is the role of JAK molecules (particularly JAK1) in the initiation of colitis in such animals**": Indeed, in *Casp1* KO mice with the absence of inflammasome singling, the superior efficacy of JAK1 inhibitors compared to Tofacitinib disappeared, instead they showed similar protective function as Tofacitinib during colitis (Fig. 5E and G, shown above in response to Comment#1). This actually indicates that the higher efficacy of JAK1 inhibitors is, at least in parts, due to the inhibition of the inflammasome. Since JAK1 inhibitors and Tofacitinib inhibited JAK/STAT signaling to a similar extent (evidenced by the decrease of cytokine production shown in Fig. 1H, Sup. Fig. 4C), but only JAK1 inhibitors were able to inhibit inflammasome signaling. Our data indicate that the effect of the JAK1 inhibitors on inflammasome activation is likely responsible for their superior effect in inhibiting intestinal inflammation. We added this point in the manuscript: '*To*

assess whether the protective effect of Filgotinib and Upadacitinib in DSS-colitis is indeed dependent on inflammasome activation, we next used Casp1-deficient mice. We observed a small and equal protective effect from all three drugs based on histology scores (Fig. 5G), but not based on endoscopy (Fig.5E) in Casp1-deficient mice. This supports the notion that these drugs exert their effects also through the suppression of the JAK/STAT pathway. However, as for Upadacitinib and Filgotinib the effect was much lower in Casp1-deficient mice when compared to wild type mice, suggesting that the inhibition of the inflammasome in conjunction with JAK/STAT pathways contributed to the advanced efficacy of JAK1 inhibitors.' (Line: #395-403). While investigating the function of these drugs on the JAK/STAT pathway in *Casp1*-deficient mice might provide additional information about the interaction between JAK inhibitors and JAK/STAT pathway, we believe this falls outside the central focus of this work, and we aim to address this in future studies.

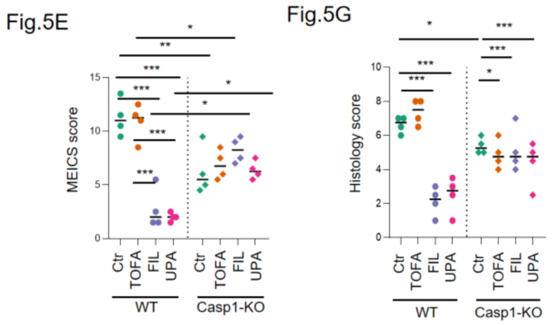

Figure 5E and 5G. The protective effect of Filgotinib and Upadacitinib is abrogated in Caspase1-deficient mice. **E)** respective scoring of mouse endoscopy on the last day of the experiment; **G)** scoring for disease severity of H&E-stained sections of the terminal colon. *p<0.05, **p<0.01, ***p<0.001, ANOVA with Holm-Sidak's multiple comparison test.

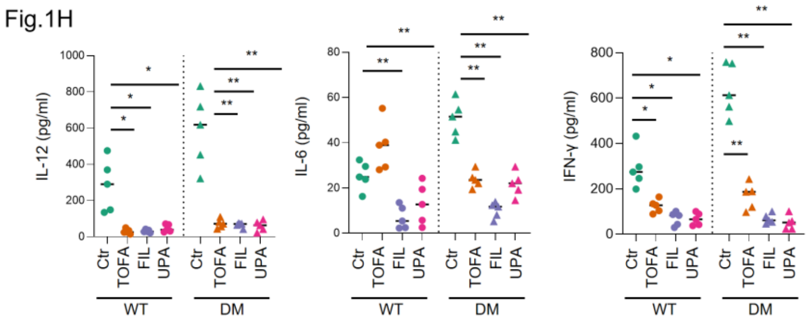

Figure 1H. Levels of the indicated cytokines from the supernatant of colon explants. Colon pieces were obtained on the last day (Day7) of the DSS model and incubated for 24 h in cell culture medium. *p<0.05, **p<0.01, ANOVA with Holm-Sidak's multiple comparison test.

Sup.Fig.4C

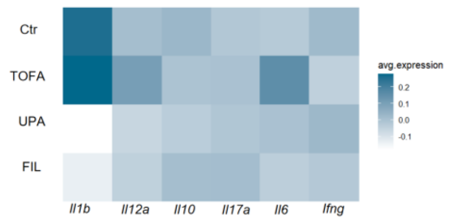

Supplementary Figure 4C. Single-cell RNA sequencing analysis of murine colonic immune cells. CD4+ T cells and CD11b+ and/or CD11c+ cells were isolated and FACS sorted from mouse colon samples and merged in a 1:1 ratio. Gene expression was examined by single-cell analysis. Average expression of the indicated genes among groups was shown in the heatmap.

9. Figure 6 (Figure 7 in the new version): These studies must be complemented by similar experiments using Filgotinib and Tofacitinib. In addition, inflammasome activation should be assessed not only by single-cell RNA sequencing but also at the protein level. It is mandatory to assess additional samples taken from both Crohn's disease patients and Ulcerative colitis patients and ascertain whether there is any difference between these two groups in terms of response to the single drugs. Mucosal expression of IL6, IL-12, and IFN-γ should also be evaluated, and the clinical and demographic characteristics of all patients must be indicated. From the text, it is not clear the type of IBD of these 3 patients. It is also very important to have data on patients' concomitant therapies because steroids are known to inhibit the inflammasome and this could distort the data.

It is not possible to enroll tofacitinib- and filgotinib treated Crohn's patients as these drugs have no approval for this indication. Also in UC, Tofacitinib-treated patients are quite rare now as this drug induces a high risk of developing thrombosis and thus has a high-risk FDA warning. We therefore focused our analysis on Upadacitinib treated patients with ulcerative colitis. We evaluated IL-1β expression using IHC in sigmoid tissue of patients with UC, and observed a reduction of IL-1β upon Upadacitinib treatment. Furthermore, decreased IL-1β levels were associated with an improvement of colitis severity. In contrast, patients who did not benefit from Upadacitinib treatment showed unchanged level of IL-1β (Fig. 7F). Mucosal expression of IL-6, IL-12, and IFN-γ was evaluated by single-cell analysis, which did not show much difference (Sup.

Fig. 7D), and we thus did not measure these cytokines again. However, we did include as control patients before and after treatment with anti-TNF to rule out that the observed effects are merely due to lower colitis activity. (Sup. Fig. 8, shown above in response to Comment#1)

As for the treatment with steroids: ‘*Of note, patients with high IL-1β expression and therapeutic response to Upadacitinib (Figure 7F) were not exposed to any other treatments. However, two of the three patients with low pre-treatment IL-1β expression had received Budesonide foam or Prednisone. Further studies will be critical to assess, if this pretreatment did impact the efficacy of Upadacitinib.*’ (Line: #443-446)

Fig.7F

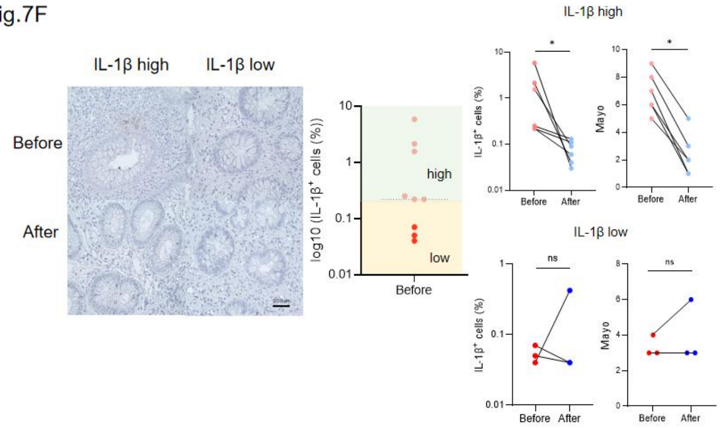

Figure 7C and 7F. Upadacitinib downregulates *NLRP3* and *IL1B* expression in UC patients. Immunocytes isolated from lamina propria of IBD patients treated with Upadacitinib were analyzed by single- cell RNA sequencing. **C)** Violin Plot of IL1B expression before and after Upadacitinib treatment. P-value was adjusted by Holm-Bonferroni method. **F)** IHC of IL-1β in sigmoid of Upadacitinib. Left: representative pictures showing IL-1β expression (brown). Middle: quantified IL-1β expression before Upadacitinib treatment (baseline). Patients were grouped to IL-1β high or low group based on the median of IL-1β expression among all individuals. Dotted line represents the median value. Right upper lane: quantified IL-1β expression and Mayo score of the patients in “IL-1β high” group before and after Upadacitinib treatment. Right lower lane: quantified IL-1β expression and Mayo score of the patients in “IL-1β low” group before and after Upadacitinib treatment. Significance was analyzed using Wilcoxon test, *$p<0.05$.

Sup. Fig. 7D

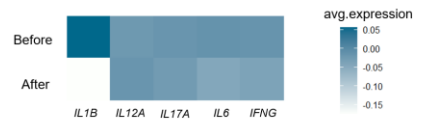

Supplementary Figure 7D. Gene expression of the cells from UC patients. Cells in lamina propria from patients before and after Upadacitinib treatment were isolated and Gene expression was determined by RNA single-cell analysis. **D)** Average expression of indicated genes in myeloid cells before and after Upadacitinib treatment.

10. Figure 7 (Figure 6 in the updated version). Panel A. The mRNA level of NLRP3 after LPS stimulation was reduced by Tofacitinib and not only by Upadacitinib and Filgotinib as stated by the authors. This is in line with previous studies showing that inhibition of Stat3 activation (a target of Tofacitinib) reduces NLRP3 expression (Exp. Neurol. 302, 104–111. 10.1016). Even the secretion of IL-18 in response to NLRP3 activator was consistently reduced by Tofacitinib and the difference (with untreated cells) was not probably significant due to the small number of experiments performed. These issues must be better addressed.

We fully agree with the reviewer that '**the mRNA level of NLRP3 after LPS stimulation was reduced by Tofacitinib and not only by Upadacitinib and Filgotinib**'. However, when assessing IL-1β protein level, Tofacitinib had only a minor effect compared to Upadacitinib. Thus, lowering NLRP3 RNA level alone does not seem to be sufficient to reduce IL-1β production. Of note, JAK1 inhibitors displayed a more pronounced suppressive effect on IL-1β production, likely associated with their direct impact on the NLRP3 inflammasome activation rather than only affecting the transcriptional regulation. The reviewer is correct: "**Even the secretion of IL-18 in response to NLRP3 activator was consistently reduced by Tofacitinib and the difference (with untreated cells) was not probably significant due to the small number of experiments performed**". We therefore discuss this now in the manuscript: '*…there was an effect of Tofacitinib on IL-18 release (although not significant, probably due to a low sample size), however, again, this effect was clearly less pronounced than the effect of Upadacitinib and Filgotinib, which reinforces our conclusion that the functional inhibition of NLRP3 seems to be more important in modulating IL-1β production than merely reducing NLRP3 transcript level.*'(Line:#365-369).

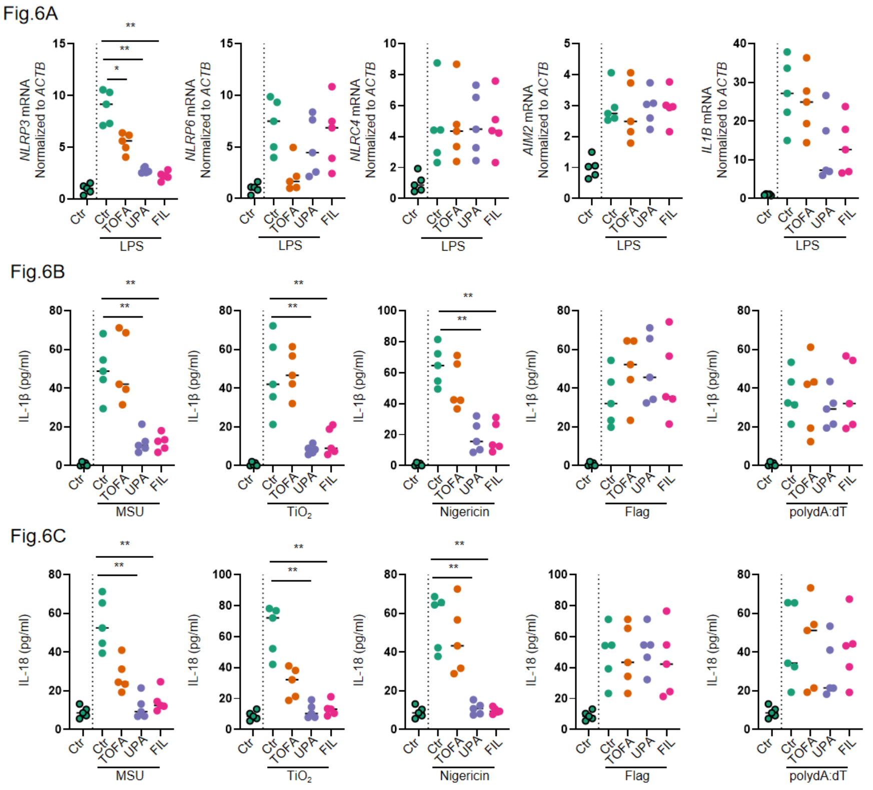

Figure 6A-C. Upadacitinib and Filgotinib specifically inhibit the NLRP3 inflammasome. Monocytes were isolated from the peripheral blood of healthy volunteers and differentiated into macrophages for 7 days. **A)** Macrophages were treated for 24 h with LPS and mRNA expression levels of the indicated inflammasome receptors were assessed; **B+C)** Macrophages were treated for 24 h with LPS and then activated with MSU (150ng/ml, 6 h), TiO2 (150 ng/ml, 12 h), Nigericin (200ng/ml, 6 h), Flagellin (Flag, 500ng/ml, 2 h) or poly(dA:dT) (1ug/ml, 2h). Cell culture supernatant was assessed for IL-1β (B) and IL-18 (C) by ELISA.

Panel D. Although the data show that Upadacitinib and Filgotinib fit into the catalytic site of NLRP3 in a similar way as the known NLRP3 inhibitor MCC950, they are not sufficient to prove that these drugs inhibit directly NLRP3 activation. Further experimentation is needed to assess the inhibitor effect of Upadacitinib and Filgotinib NLRP3 activation in JAK1-deficient cells.

We thank the reviewer for this point. In order to address this, apart from the computational modeling (Figure 6E), we assessed the effect of Upadacitinib and Filgotinib in *JAK1*-deficient as well as *NLRP3*-deficient cells (Figure 6D, shown above in response to Comment#1). We found that Upadacitinib and Filgotinib reduced IL-1β expression in *JAK1*-deficient but not in *NLRP3*-deficient macrophages. This indicates

that JAK1 inhibitors suppress the NLRP3 activation independently of JAK1 signaling, but dependent on NLRP3.

Fig.6E

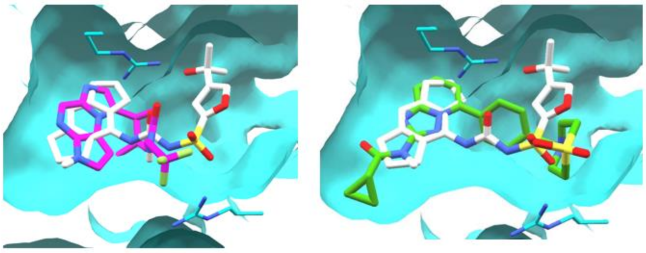

Figure 6E. Structural overlay of Upadacitinib (carbon atoms in magenta, left side) or Filgotinib (carbon atoms in green, right side) to MCC950 (carbon atoms in white) in the MCC950-binding pocket of NLRP3 (cyan surface). The side chains of Arg351 and Arg578 (cyan sticks) form similar polar interactions with the two ligands.

Reviewer #2 (Remarks to the Author):

Relevant topic for the field of IBD and understanding pathways for the novel therapies is timely. A few comments to consider:

General

Level of inflammation needs to be accounted for in the experiments. Tofa treated mice have more inflammation therefore will have more IL1b+ dependent cells and associated pathways? What efforts have been made to adjust for inflammation rather than treatment driving the outcome

We agree with the reviewer. Therefore, to avoid this potential caveat, for our single cell sequencing analysis we sacrificed mice on day 5 after DSS induction, when mice across the groups were under equal disease scores (Fig.1B and C, upper lane, attached below). In addition, we did functional experiments using *Casp1*-deficient mice to demonstrate the relevancy of the inflammasome.

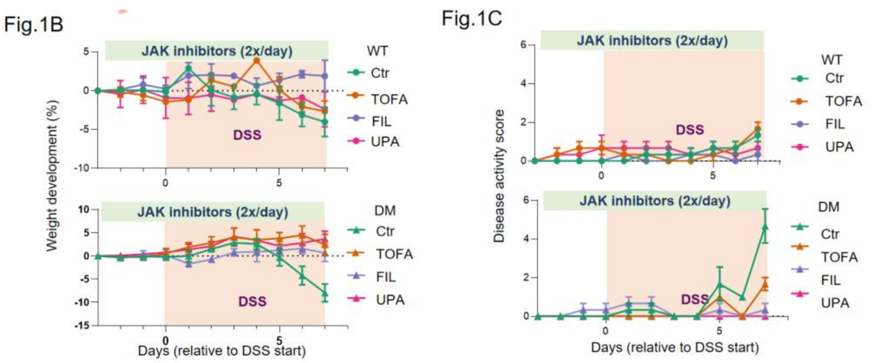

Figure 1B and C. Upadacitinib and Filgotinib reduce colitis severity. PTPN2-LysMCre (PTPN2-ΔM) mice and their WT littermates were orally gavaged with the JAK inhibitors Tofacitinib, Filgotinib, Upadacitinib starting three days prior to administration of 2% DSS in the drinking water for 7 days. **B)** weight development; **C)** disease activity score over time.

Figure 1 and 5

B and C are unintelligible with 8 overlapping lines, these need to be redone

We thank the reviewer for this point. To make it easier to distinguish between the

different groups, we now separated the WT from the PTPN2-mutated mice.

The severity of colitis in control mice in B and C and in tofa treated mice in Figure 5 C are very different to each other and in Figure 1 very mild

The reviewer is right that the colitis severity is different in the control mice between these experiments. However, these experiments were not performed simultaneously. Indeed, we do observe a variability from experiment to experiment, which is why we always use the appropriate control. The control mice in Figure 1 were littermates to the mice lacking PTPN2 in myeloid cells, while the control mice used for the experiments shown in Figure 5 were WT mice co-housed with *Casp1*-deficient mice. Thus, these control mice were bred completely separately. Therefore, one can only compare those groups with each other that were in the same experiment.

It is not clear how many times in the legend these experiments were performed and in how many mice per group?

The data presented is representative for one out of two independent experiments with n=6 per group. We now included this information in each Figure legend.

Were there sex differences observed?

Male mice become aggressive and often need to be removed from experiments. Therefore, the animal license in Zurich did not allow to use male mice for DSS colitis experiments and in general, for DSS colitis, only female mice are used due to animal welfare reasons. Hence, we are not able to tell if there are sex differences for the observed effects. Of note, in Hamburg we can use male and female mice in colitis models, and we did not observe any differences between the genders.

Figure 2

M1 and M2 macrophages is an outdated term and only relates to in vitro blood not tissue cells. These terms need to be removed and the annotation criteria explained for all cell types

We updated the annotation and the macrophages are divided to premature and mature populations based on the expression of *Cx3cr1* (Sup. Fig. 4D).

Sup.Fig.4D

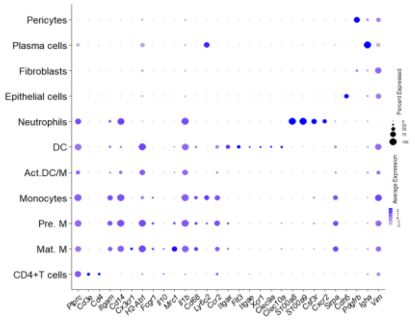

Supplementary Figure 4D. Single-cell RNA sequencing analysis of murine colonic immune cells. CD4+ T cells and CD11b+ and/or CD11c+ cells were isolated and FACS sorted from mouse colon samples and merged in a 1:1 ratio. Gene expression was examined by single-cell analysis. D) dot plot of characteristic markers in different cell clusters.

Absolute numbers of cell per cluster?

We included the absolute number in the attached excel sheet. We are happy to include it in the Figure upon request. But we would prefer to indicate the frequency.

Cluster allocation by disease

We included only one disease, which is colitis.

Where are the monocytes? DSS is a model of monocyte recruitment?

Based on this comment, we re-ran the Umap and updated the annotation. We now label the monocytes in the respective cluster (Figure 2D).

Fig. 2D

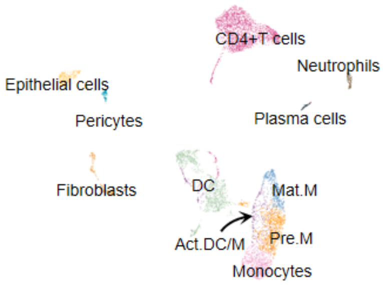

Figure 2D. UMAP of identified cell clusters of immune cells isolated from control and JAK inhibitor- treated groups.

What cell types are used in the DEG in panel C?, should his not be split by cluster to show macrophages are the most affected cell type?

All cells were included to analyze the DEGs, because we did not know at the beginning which cell type or signaling would dominate the phenotype. Therefore, to start in an unsupervised way, we included all cell clusters. Based on these results we focused on IL-1, and we found macrophages contributed the most to IL-1 signaling.

The text terms "leucocytes" but this is actually CD4+ cells and CD11b/CD11c+ cells mixed in a 1:1 ratio

Thanks for the reviewer rising this point. We changed '*leucocytes' to 'myeloid cells (CD11b*$^{+}$ *and/or CD11c*$^{+}$*) and CD4*$^{+}$ *T cells (with a 1:1 ratio combination)*' to be more specific in the manuscript. (Line: #150-151)

As such interpreting relative abundance in panel F is unhelpful, was flow cytometry analysis done?

Relative abundance is a possible way to show the cluster composition in different groups and to visualize overall alteration of clusters in single-cell sequencing analyses. This is also recommended in the current best practice guidelines as well as in recent publications (Lukas Heumos et al., Single-cell best practice, 2023, Chapter 17.3) (A publication as an example: Alvarez-Martinez et al., *Nat Immunol*, 2024). Of note, this analysis merely serves to generate a hypothesize, which then was tested in the subsequent experiments and analyses.

Figure 4

There is a clear bi modal distribution for many of the cytokines in Tofa treated WT and variant differentiated monocytes. Why is this? How many independent experiments were performed? If on separate days how was this variation accounted for?

We appreciate this point. Many factors including genetic diversity, gender, age, microbiota composition, and environmental factors, contribute to the variability observed in human samples. These could cause relatively big deviation or bi modal distribution as noted by the reviewer. All samples shown in Fig. 4 were processed at the same day, in order to avoid further discrepancy.

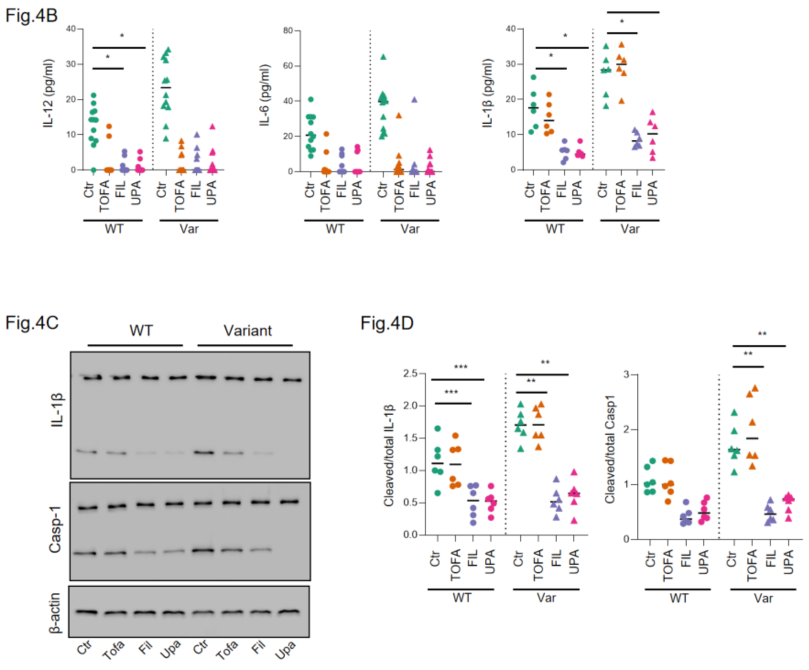

Figure 4B-D. Upadacitinib and Filgotinib inhibit inflammasome activation in human cells. Monocytes were isolated from the peripheral blood of IBD patients that were either WT or variant for the loss-of-function SNP rs1893217 in the gene locus encoding PTPN2. These cells were differentiated into macrophages and then treated for 24h with LPS prior to activation with MSU crystals (150 ng/ml) for 6h. B) cytokine levels in the supernatant for the indicated cytokines; C) representative Western blot pictures; D) densitometry for the indicated proteins. *p<0.05, **p<0.01, ANOVA with Holm-Sidak's multiple comparison test.

Figures 5

A-C are unintelligible and need to be seperated / made clearer

We separated the WT and the *Casp1*-deficient mice to make it clearer.

Figure 6 (Figure 7 in the upadated version)

How were these cells annotated?

The feature genes were shown in the Sup. Fig. 7C.

Sup. Fig.7C

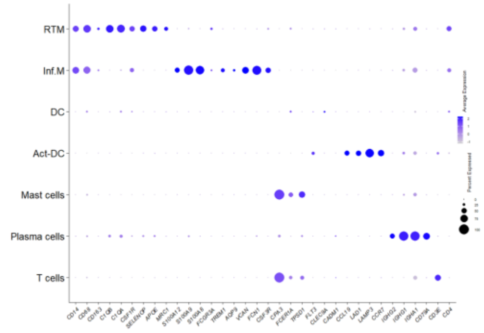

Supplementary Figure 7C. Clusters and gene expression of the cells from IBD patients. Cells in lamina propria from patients before and after Upadacitinib treatment were isolated and Gene expression was determined by RNA single-cell analysis. The feature gene expression in each identified cluster.

Can authors report Absolute cells per cluster?

We summarized this in an Excel sheet (enclosed with the manuscript) as requested.

Were cluster allocation split by treatment?

No, all the samples were integrated together and allocated. We did not observe the allocation split by treatment.

Reply Fig. 3

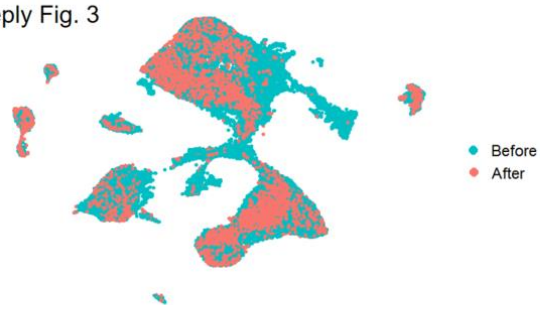

Reply Figure 3. Umap showing integrated paired samples from patients (n=4) before and after Upadacitinib treatment.

Panel C top of feature plot has been cut off

We thank the reviewer for this point. We updated the figures and resolved this problem. (This figure has been moved to supplementary data as Supplementary figure 7B.)

Have authors done any Pathway analysis on macrophages pre and post treatment? NLRP3/IL1b?

As suggested by the reviewer, we did a pathway analysis on macrophages based on the differentially expressed genes (DEGs) between pre and post Upadacitinib treatment (Reply Fig.3). We did not see the NLRP3/IL1B signaling pathway in the top list. Therefore, while the transcriptomic analysis was valuable to generate hypotheses, the subsequent pathway analysis may have guided us to another direction. Of note, our data do not exclude an additional role of other pathways. But we believe that studying those, would be outside the scoop of this manuscript. Nevertheless, our data clearly demonstrated that IL-1β, primarily expressed by macrophages, significantly decreased on RNA and protein level upon Upadacitinib treatment in the UC patients. And the reduction of IL-1β is closely associated with therapeutic response (Fig.7F).

Reply Fig. 4

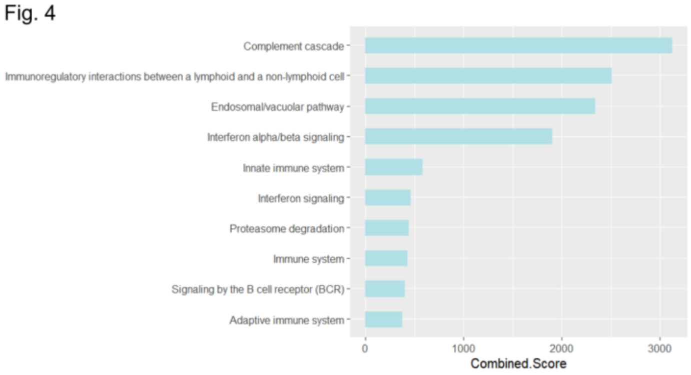

Reply Figure 4. Enriched pathway analysis based on the differentially expressed genes (DEGs) on macrophages between pre and post treatment of Upadacitinib patients with UC. Top 10 signaling is listed.

Fig.7F

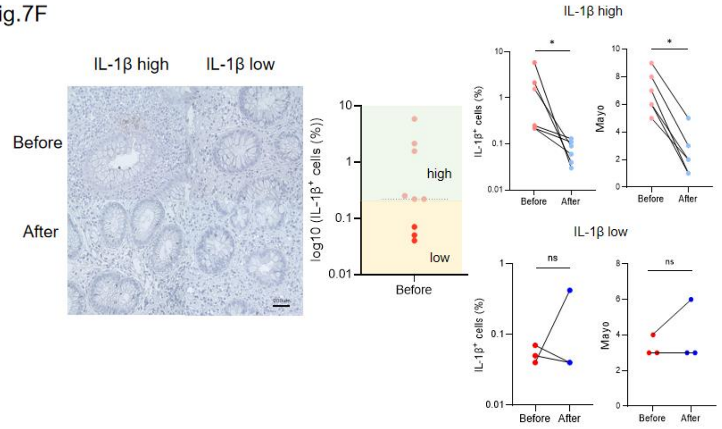

Figure 7F. Upadacitinib downregulates IL-1β *production* in UC patients**.** IHC of IL-1β in

sigmoid of Upadacitinib. Left: representative pictures showing IL-1β expression (brown). Middle: quantified IL-1β expression before Upadacitinib treatment (baseline). Patients were grouped to IL-1β high or low group based on the median of IL-1β expression among all individuals. Dotted line represents the median value. Right upper lane: quantified IL-1β expression and Mayo score of the patients in “IL-1β high” group before and after Upadacitinib treatment. Right lower lane: quantified IL-1β expression and Mayo score of the patients in “IL-1β low” group before and after Upadacitinib treatment. Significance was analyzed using Wilcoxon test, *$p<0.05$.

Figure 7 (Figure 6 in the updated version)

D what controls were used?

We used MCC950, a well-characterized NLRP3 inhibitor, as control. We compared the catalytic structural similarity between MCC950, Upadacitinib and Filgotinib, and found an overlap of MCC950 with these two JAK1 inhibitors. In addition, computational modeling also simulated the possible direct interaction of NLRP3 and JAK1 inhibitors.

Reviewer #3 (Remarks to the Author):

The current manuscript entitled "JAK1 inhibitors target the NLRP3 inflammasome thereby suppressing inflammatory bowel disease" aims to unravel the molecular mechanism of action of JAK1 inhibitors in IBD. Unfortunately, the manuscript suffers from several major flaws.

- There is no direct evidence using NLPR3 targeting in mice that JAK inhibitors work by controlling NLRP3.

We appreciated this point from the reviewer. Unfortunately, we do not have *Nlrp3*-deficient mice at the moment. We thus used *Casp1*-deficient mice to assess the role of the inflammasome *in vivo*. Subsequently, we used *in vitro* experiments to assess the relevancy of NLRP3. We activated NLRP3 by MSU, TiO2 and Nig in human monocyte-derived macrophages, and used MCC950, a well characterized NLRP3 inhibitor to block NLRP3. Simultaneously we added JAK inhibitors on top. Our data revealed that Upadacitinib and Filgotinib alone can inhibit IL-1β production as sufficiently as MCC950, but Tofacitinib does not (Sup.Fig.5A). Additionally, Upadacitinib and Filgotinib could not further reduce IL-1β when MCC950 was present (Sup.Fig.5A).

Furthermore, we added an experiment where we knocked down *JAK1* or *NLRP3* using siRNA and human macrophages. Of note, JAK1 inhibitors were still capable of inhibiting the production of IL-1β upon the knock-down of *JAK1*, but not upon the knock-down of *NLRP3* (Figure 6D). This indicates that JAK1 inhibitors regulate NLRP3 directly, and independent of JAK1 signaling. We believe that these data together allow us to better support our conclusion that JAK1 inhibitors target NLRP3. However, we would be willing to repeat the in vivo experiment using *Nlrp3*-deficient mice, if deemed essential.

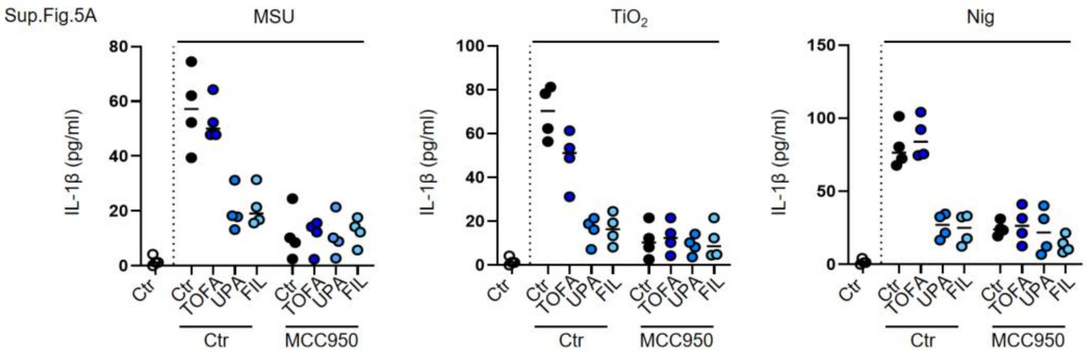

Supplementary Figure 5A. No additive effect of JAK1 inhibitors with the presence of NLRP3 inhibitor MCC950. Monocytes were isolated from the peripheral blood of healthy volunteers and

differentiated into macrophages for 7 days. Macrophages were treated for 24 h with LPS and then activated with MSU (150ng/ml, 6 h), TiO2 (150 ng/ml, 12 h), Nigericin (200ng/ml, 6 h), Flagellin (Flag, 500ng/ml, 2 h) or poly(dA:dT) (1ug/ml, 2h). Cell culture supernatant was assessed for IL-1β.

Fig. 6D

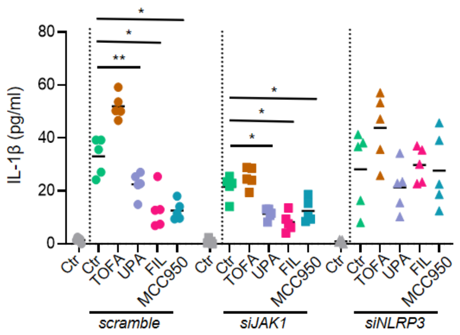

Figure 6D. Macrophages were transfected by JAK1, NLRP3, or scramble control siRNA respectively for 48 h, then treated with LPS alone, or LPS with different JAK inhibitors or MCC950 respectively for 10h. Cell culture supernatant was assessed for IL-1β by ELISA.

- Observations are limited to a series of colitis prevention studies in a single model. How can these findings predict response to therapy in established IBD inflammation?

To address this point, we included data from a second IBD mouse model, i.e. T cell transfer colitis model (Sup. Fig. 1) where we see the same effects as in the DSS model. More importantly, we expanded our analysis with additional human data. By performing single cell analysis and immunohistochemistry, we found a reduction of IL-1β expression upon Upadacitinib treatment. Notably, the reduction of IL-1β was associated with therapeutic response (Fig.7C and F). While our current findings are not sufficient to predict response to therapy in established IBD inflammation, we believe that our data build the basis for a follow up study in which this question can be answered. We discussed this in the manuscript: '*Overall, our data suggest that the efficacy of Upadacitinib therapy in ulcerative colitis might be correlated to its capacity to inhibit IL-1β activation via inhibition of the NLRP3 inflammasome. Therefore, Upadacitinib might work more efficiently in UC individuals with high IL-1β expression, which indicates IL-1β could potentially serve as a biomarker to assist clinical therapeutic strategy. However, future multi-center prospective trials with higher patient numbers will be essential to test this hypothesis.*' (Line: #451-456)

Sup.Fig.1

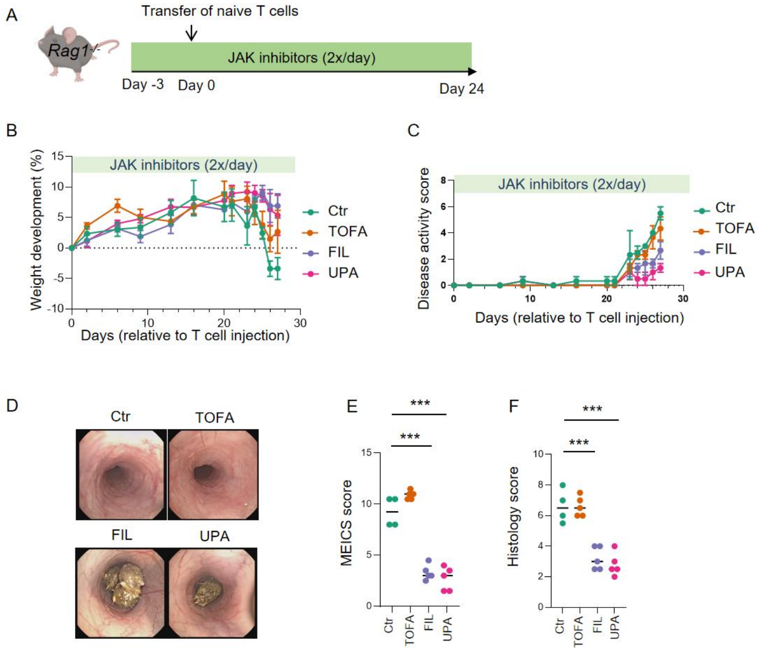

Supplementary Figure 1. Upadacitinib and Filgotinib reduce T cell mediated colitis**.** *Rag1*$^{-/-}$ mice were orally gavaged with the JAK inhibitors Tofacitinib, Filgotinib, Upadacitinib starting three days prior of the injection of 0.25x10^{6} naïve T cells. **A)** Experimental set-up; **B)** weight development; **C)** disease activity score over time; **D)** representative images of colitis and **E)** respective scoring of mouse endoscopy on the last day of the experiment. ***p<0.001, ANOVA with Holm-Sidak's multiple comparison test.

Fig.7C

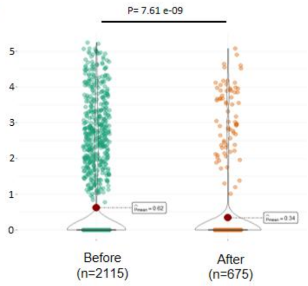

Fig.7F

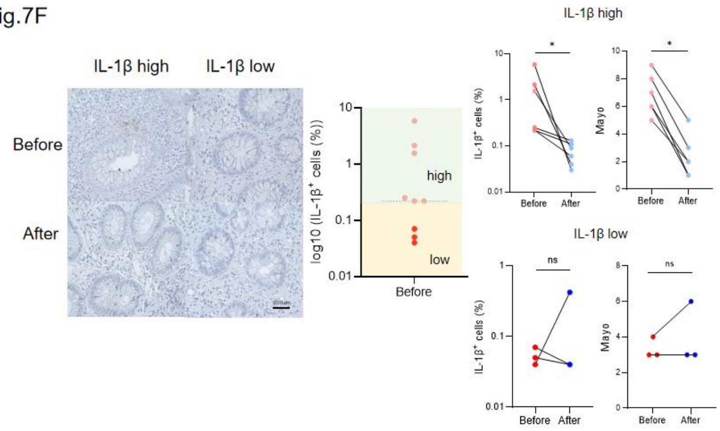

Figure 7C and 7F. Upadacitinib downregulates *NLRP3* and *IL1B* expression in UC patients. Immunocytes isolated from lamina propria of IBD patients treated with Upadacitinib were analyzed by single- cell RNA sequencing. **C)** Violin Plot of IL1B expression before and after Upadacitinib treatment. P-value was adjusted by Holm-Bonferroni method. **F)** IHC of IL-1β in sigmoid of Upadacitinib. Left: representative pictures showing IL-1β expression (brown). Middle: quantified IL-1β expression before Upadacitinib treatment (baseline). Patients were grouped to IL-1β high or low group based on the median of IL-1β expression among all individuals. Dotted line represents the median value. Right upper lane: quantified IL-1β expression and Mayo score of the patients in “IL-1β high” group before and after Upadacitinib treatment. Right lower lane: quantified IL-1β expression and Mayo score of the patients in “IL-1β low” group before and after Upadacitinib treatment. Significance was analyzed using Wilcoxon test, *$p<0.05$.

- *How do the authors explain the lack of effect of tofacitinib on colitis? Can they demonstrate that serum levels of this drug in mice are similar to those in humans on drug therapy?*

We understand that the reviewer is concerned due to the absence of an effect of the Tofacitinib treatment in our experiments. The lack of a significant effect on colitis severity following Tofacitinib treatment in our wild-type mice is consistent with previous findings (Spalinger et al., 2021; De Vries et al., 2019). Notably, Tofacitinib does influence inflammatory cytokine levels (Figure 1H) and modulates STAT phosphorylation in the intestinal tissue (Reply Figure 1, shown below). Furthermore, Tofacitinib exerts a clear therapeutic effect in mice lacking PTPN2 specifically in myeloid cells, indicating that the drug retains functional activity under certain conditions. Importantly, increasing the dosage of Tofacitinib did not lead to improved outcomes in wild type mice, suggesting that the lack of efficacy was not due to insufficient drug exposure. Even at doses as high as 450 mg/kg—at which serum levels are expected

to reach or exceed effective concentrations (Texler et al., Cell Mol Gastroenterol Hepatol, 2021; Maiga et al., eBioMedicine, 2015; Vong et al., Clin Pharmacol Drug Dev, 2021; Julsgaard et al., Lancet Gastroenterol Hepatol, 2023)—Tofacitinib did not alleviate colitis in the DSS-colitis model (We discussed this point in the manuscript, line: #323-329). These findings collectively suggest that inhibition of the JAK/STAT pathway alone, as mediated by Tofacitinib, is less effective than the combined inhibition of JAK/STAT signaling and IL-1β/inflammasome activation, as mediated by JAK1 inhibitors, at least in wild type animals.

In contrast, in PTPN2-deficient mice—where JAK/STAT signaling is more strongly activated—Tofacitinib showed therapeutic efficacy. These results suggest that Tofacitinib may be particularly effective in IBD patients carrying PTPN2 polymorphisms (We discussed this point in the manuscript, line: #318-321), while JAK1-selective inhibitors such as Filgotinib and Upadacitinib may have broader clinical utility. Further studies will be essential to test this hypothesis. Nevertheless, our data demonstrate that the dual inhibition of the JAK/STAT pathway and the NLRP3–IL-1β axis contributes to the superior efficacy observed with Filgotinib and Upadacitinib (Line: #461-466).

Fig.1H

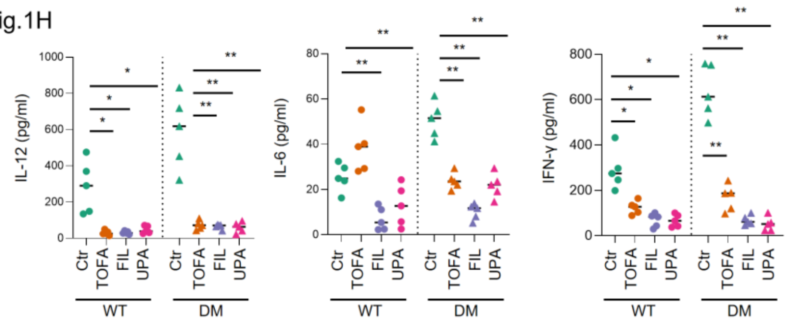

Figure 1H. Levels of the indicated cytokines from the supernatant of colon explants. Colon pieces were obtained on the last day (Day7) of the DSS model and incubated for 24 h in cell culture medium. *p<0.05, **p<0.01, ANOVA with Holm-Sidak's multiple comparison test.

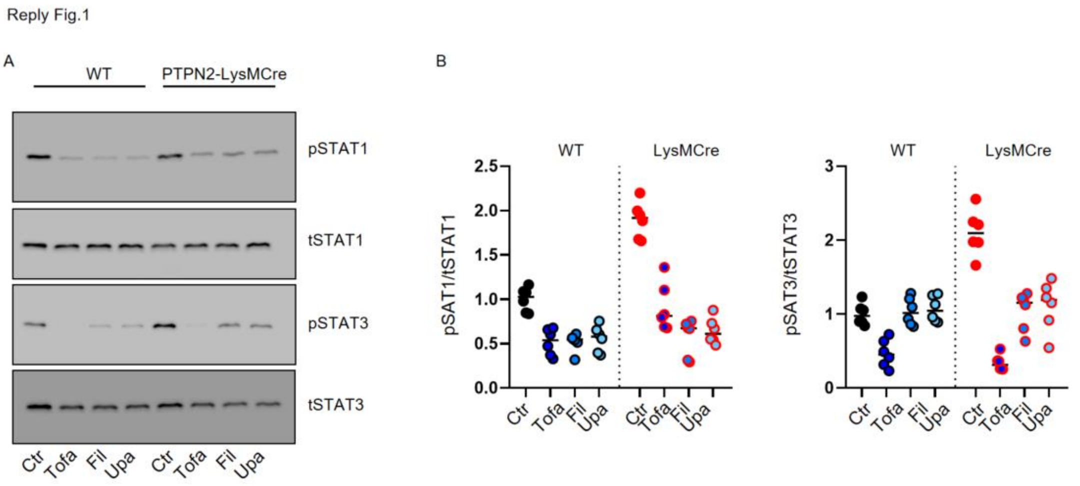

Reply Figure 1. Phosphorylation of STAT1 and STAT3 upon the treatment of JAK inhibitors in mouse colon lysates after DSS administration. **A)** representative pictures and **B)** densitometric analysis of Western blots for the indicated proteins.

- Why does tofacitinib induce colitis in WT mice and block colitis in knockout mice (Fig. 1E)?

In *Ptpn2*-knockout mice, hyperactivated JAK/STAT signaling is the primary driver of colitis, making Tofacitinib an effective therapeutic agent. In contrast, in WT mice, JAK/STAT signaling is only partially responsible for driving colitis, thus inhibition of this pathway alone seems not to be efficient. However, the drug did not promote colitis in WT mice.

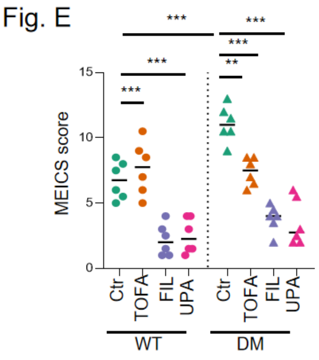

Figure 1E. Upadacitinib and Filgotinib reduce colitis severity. PTPN2-LysMCre (PTPN2-ΔM) mice and their WT littermates were orally gavaged with the JAK inhibitors Tofacitinib (n=6), Filgotinib(n=6), Upadacitinib (n=6) starting three days prior to administration of 2% DSS in the drinking water for 7 days. **E)** respective scoring of mouse endoscopy on the last day of the experiment.

- Tofacitinib inhibits JAK1. How do the authors explain the lack of efficacy in DSS colitis?

We thank the reviewer for this point. Though Tofacitinib did not rescue mice from colitis, we indeed observed the reduced production of pro-inflammatory cytokines using JAK/STAT pathway in Tofacitinib-treated WT mice (Fig. 1H). Our data indicate that inhibiting JAK/STAT pathway alone is not sufficient to ameliorate DSS-colitis at least in WT mice. But its combination with NLRP3 blockade enhances the therapeutic efficacy. Of note, NLRP3 inhibition by JAK1 inhibitors appears to be independent on JAK1 signaling itself (Figure 6D, shown above in response to the first comment). And we have discussed this point in our manuscript: '*Notably, in the mouse colitis model, we also observed a reduction in several other pro-inflammatory cytokines—such as IL-12, and IFN-γ. However, both Tofacitinib and the JAK1 inhibitors reduced these cytokines to comparable levels, suggesting that they are unlikely to be the primary drivers of the different therapeutic efficacy observed. This interpretation is further supported by our single-cell analysis, which did not implicate these cytokines as key factors underlying the distinct responses to the treatments. Collectively, our findings indicate that inhibition of the NLRP3–IL-1β axis plays a critical role in mediating the superior efficacy of Upadacitinib and Filgotinib compared to Tofacitinib in the context of ulcerative colitis. Nonetheless, our data do not exclude the possibility that other cytokines, including IL-6, IL-12, and IFN-γ, and other pathways (Figure 2C) may also contribute. Further studies will be necessary to elucidate the specific functional roles of these cytokines and pathways in modulating treatment responses.*' (Line: #372-383).

- JAK inhibitors should block effects on multiple immune cell subsets, not just macrophages. How do the authors rule out effects on other immune cell subsets?

Very likely there are other molecules and pathways mediating the efficacy of JAK inhibitors. But the new mode of action of JAK1 inhibitors is allocated on the inflammasome-IL1β axis. Our data indicate that macrophages are the primary target (Fig.2E and F). We fully agree that other immune cells and pathways might be also affected. Further studies are warranted to fully decipher the effect of JAK1 inhibitor. But addressing this is clearly beyond the scope of this manuscript. Nevertheless, we do discuss this in the manuscript: '*Collectively, our findings indicate that inhibition of the NLRP3–IL-1β axis plays a critical role in mediating the superior efficacy of Upadacitinib and Filgotinib compared to Tofacitinib in the context of ulcerative colitis. Nonetheless, our data do not exclude the possibility that other cytokines, including IL-6, IL-12, and IFN-γ, and other pathways (Figure 2C) may also contribute. Further studies will be necessary to elucidate the specific functional roles of these cytokines and pathways in modulating treatment responses.*' (Line: #377-383)

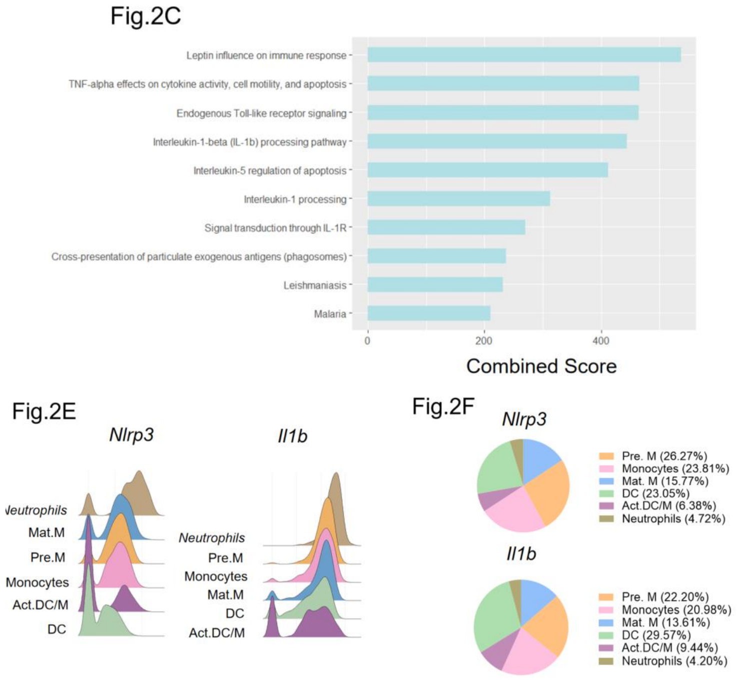

Figure 2C, 2E and 2F. Upadacitinib and Filgotinib downregulate *Nlrp3* and *IL1b* expression. Colonic immunocytes from DSS colitis after JAK inhibitor treatment were analyzed by single-cell RNA sequencing. CD4$^+$T cells and CD11b$^+$ and/or CD11c$^+$ cells were isolated and FACS sorted from mouse colon, and merged by 1:1 ratio. **B)** differentially expressed genes (DEGs) between the Tofacitinib-treated group and JAK1i group (including Upadacitinib and Filgotinib); **E)** *Il1b* and *Nlrp3* expression among cell clusters; **F)** proportions of cell clusters within *Nlrp3* and *Il1b* expressing cells;

- Caspase 1 deficiency should also affect the gasdermin pathway. This needs to be investigated.

Activated Caspase-1 cleaves gasdermin D into N- and C-terminal fragments (GSDMD-N, GSDMD-C). And GSDMD-N inserts itself to the membrane, forming pores which facilitate the release of IL-1β and IL-18, while also driving the pyroptosis process. In *Casp1*-deficient mice, gasdermin cleavage is partially impaired, but can be still activated by other caspase members, e.g., Caspase-8 (Demarco et al., 2020, Sci. Adv). Since our data did not indicate a relevance of the gasdermin pathway or its connection to the efficacy of JAK inhibitors, we decided not to pursue this point further. We are happy to discuss this point in the manuscript upon request.

- To my knowledge, there is no evidence that IL-1 or IL-18 blockade is effective in human IBD. How can the authors explain that JAK1 inhibitors work by inhibiting these cytokines?

We appreciate this point. We added the discussion in our manuscript: '*Large-scale studies of IL-1 blockade in polygenic IBD patient in acute severe ulcerative colitis are ongoing, however, results appear to be rather disappointing. We hypothesize that targeting IL-1β alone is insufficient to provide effective protection against colitis. Dual action, such as targeting both the JAK/STAT signaling pathway and the inflammasome axis may increase the therapeutic efficacy. This highlights the importance of simultaneously modulating these interconnected inflammatory pathways. Thus, taken together, it appears that inhibition of NLRP3-IL-1β axis plays an important role in controlling colitis in conjunction with JAK/ STAT inhibition, but may not be sufficient to control colits alone. Further studies will be essential to clarify this point.'* (Line: #457-466).

As for IL-18, we did not find a difference in our experiments with UC patients— at least not in the single cell analysis—which is why we did not pursue this further.

To reviewers' comments:

We sincerely appreciate the reviewers' insightful comments and constructive suggestions and have refined our work accordingly. In line with these recommendations, we conducted the following key experiments:

i) We performed DSS colitis experiments in *Nlrp3*-deficient mice treated with Upadacitinib. We observed that, in the absence of NLRP3, the efficacy of Upadacitinib is largely abolished.

ii) We expanded the cohort of UC patients analyzed by immunohistochemistry for IL-1β, demonstrating that the therapeutic efficacy of Upadacitinib is associated with suppression of IL-1β in colon tissue.

iii) We performed an NLRP3 binding assay using the NLRP3 NanoBRET™ Target Engagement system to assess whether JAK1 inhibitors directly bind NLRP3. The data showed no direct binding, indicating that Upadacitinib and Filgotinib modulate the NLRP3–IL-1β axis indirectly.

iv) We evaluated the effects of Tofacitinib, Upadacitinib, and Filgotinib on monocytes, dendritic cells, and T cells to obtain a broader understanding of the overall immunomodulatory effect of JAK inhibitors.

The detailed reply to reviewers' comments is listed below:

Reviewer #1 (Remarks to the Author):

The authors have revised the manuscript, taking into consideration all my previous comments. In my opinion, the study should be accepted in its current version, as it advances our understanding of the mechanism of action of Jak inhibitors.

Editorial note: Due to the absence of reviewer 2 for this review round this reviewer was additionally asked to comment in place of the missing reviewer:

I have just now had the opportunity to read the paper and see if the authors addressed the points raised by Reviewer 2. In my opinion, they did a great job and all the relevant questions were adequately answered.

We thank the reviewer for the positive assessment of our work.

Reviewer #3 (Remarks to the Author):

The manuscript examines the different therapeutic mechanisms of various Janus kinase inhibitors in UC and has already been critically reviewed by three reviewers. Despite rejection, the authors have written a rebuttal version, which is now being re-reviewed again.

While tofacitinib and the JAK1-selective inhibitors upadacitinib + filgotinib are all used in the treatment of UC, their different efficacies and exact molecular mechanisms of action are not fully understood. In their study, the authors propose that, in addition to inhibiting the JAK/STAT signaling pathway, upadacitinib and filgotinib uniquely target and suppress the NLRP3 inflammasome, leading to a reduction in IL-1β levels. This dual mechanism of action is considered to be a key factor in their superior efficacy in UC. The study combines in vivo mouse models, in vitro experiments with human cells, and single-cell RNA sequencing data from UC patients.

The authors have made considerable efforts to address the concerns raised by the initial reviewers, thereby significantly improving the manuscript.

good points are:

- The strongest finding of the study lies in the now more convincing evidence that JAK1 inhibitors, unlike tofacitinib, can directly inhibit the NLRP3 inflammasome and subsequent IL-1β production. This finding is novel and provides a plausible explanation for the observed differences in clinical efficacy. Computer-aided modeling (Figure 6E), which shows that these inhibitors fit into the catalytic site of NLRP3 in a manner similar to MCC950, supports this claim with a solid structural basis.

- The authors now provided new siRNA knockdown data on the mechanism of inflammasome inhibition and clarified the direct effect of JAK1 inhibitors on NLRP3 inhibition. (Fig. 6D)

- They now discuss more clearly how their results differ from previous studies and emphasize the direct NLRP3 target effect of JAK1 inhibitors in contrast to the indirect effects reported for other JAK inhibitors.

-The authors now state that they have separated the data from WT and PTPN2-deficient/Casp1-deficient mice for clarity and have now provided details on the number of animals. (Fig. 1, Fig. 5)

- In the updated note, myeloid cells are now better identified as immature and mature macrophages based on Cx3cr1, and has been rerun to explicitly label monocytes. (Fig. 2D) The outdated terminology and cell identification are no longer included.

- The addition of human data from UC patients treated with upadacitinib and the re-analysis of the anti-TNF cohort now provide better evidence for the clinical relevance of their findings.

- The single-cell RNA-Seq data show that IL-1β was reduced more significantly by JAK1 inhibitors compared to other cytokines. This supports the focus on the inflammasome IL-1β signaling pathway as a key distinguishing feature. (suppl. Fig. 4C)

We thank the reviewer for the positive assessment of our work.

Several points remain still problematic:

-While the in vitro siRNA data and the data from caspase-1-deficient mice strongly suggest direct inhibition of NLRP3, the lack of data from in vivo experiments with Nlrp3-deficient mice remains a limitation. Direct in vivo evidence that the efficacy of JAK1 inhibitors is abolished in Nlrp3-deficient mice would definitively confirm this mechanism in a complex biological system. However, the authors have noted that they unfortunately do not currently have access to the mouse strain.

We thank the reviewer for this point. We imported *Nlrp3*-deficient mice and performed the requested experiment. Due to the limited time and access to this strain, we performed this experiment with Upadacitinib only. '…To further assess whether the efficacy of JAK1 inhibitors in colitis is dependent on NLRP3, we performed DSS colitis in *Nlrp3*-deficient mice (Sup. Fig. 6A,). Consistent with the findings obtained in *Casp-1*-deficient mice, Upadacitinib showed no protective effect in *Nlrp3*-deficient mice (Sup. Fig. 6B-D).' (Line: #250-253)

Sup.Fig. 6

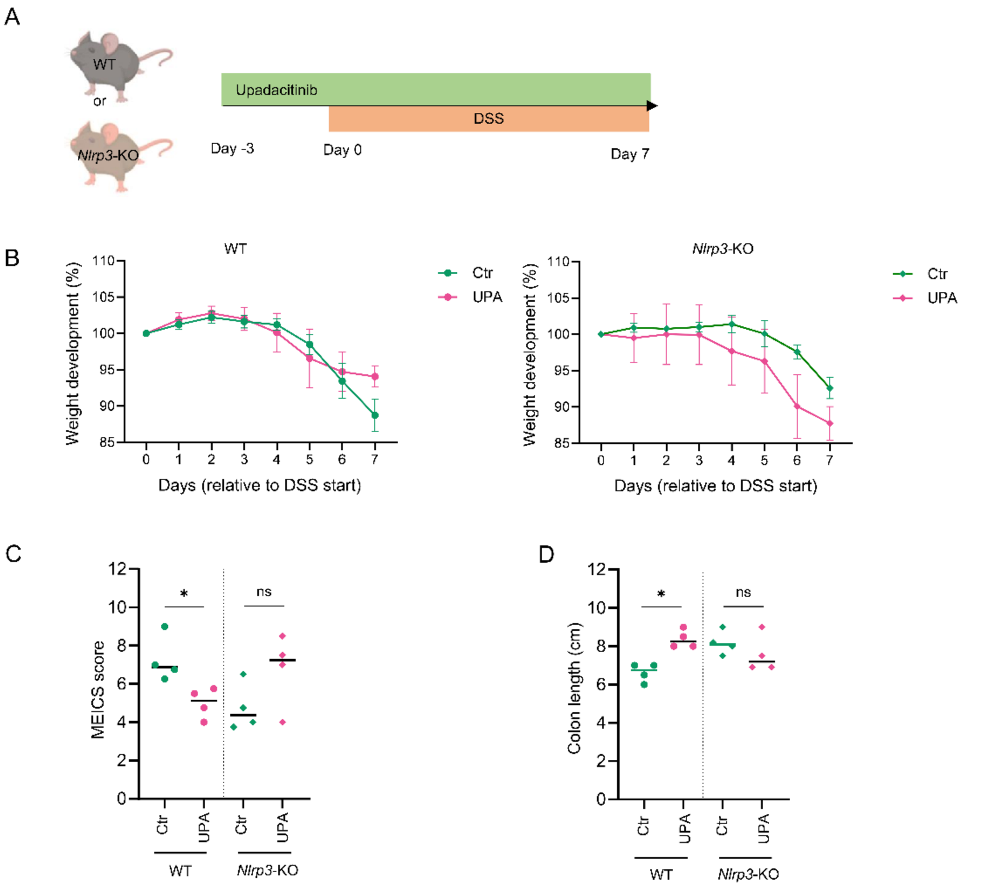

Supplementary Figure 6. The protective effect of Upadacitinib is abrogated in *Nlrp3*-deficient mice. *Nlrp3*-deficient mice or WT controls were treated with the Upadacitinib (n=4), starting three days prior to administration of 2% DSS in the drinking water for 7 days. **A)** Experimental set up; **B)** weight development; **C)** respective scoring of mouse endoscopy on the last day of the experiment; **D)** colon length. Results are from one experiment. Every dot in C and D corresponds to one mouse. *p<0.05, Mann Whitney test.

-Unfortunately, the cohort size for single-cell RNA sequencing is still small. Although these are valuable initial findings, larger multicenter studies that also include other JAK inhibitors (filgotinib and possibly even tofacitinib) would provide greater generalizability and comparable efficacy data in human patients. The recognized confounding factor of prior steroid treatment in some patients with low IL-1β levels also requires further investigation. It is worth considering whether the authors could contribute to this.

We appreciated the comments from the reviewer. The cohort of four patients for single cell RNA sequencing is relatively small. However, single cell sequencing analysis was primarily used to generate a hypothesis, which was then tested *in vitro* and in *vivo* using mouse models. However, in order to further strengthen the human data as requested, we enrolled another 7 patients in which we performed IL-1β IHC before and after Upadacitinib treatment (Figure 7F, also below). The enlarged cohort confirmed the correlation between IL-1β reduction and response to Upadacitinib therapy.

Notably, the newly recruited patients were not treated with steroid, therefore arguing against a major steroid effect. Nevertheless, validation in a larger cohort is warranted, as discussed in the manuscript: '*Of note, patients with high IL-1β expression and therapeutic response to Upadacitinib were not simultaneously treated with any other advanced IBD treatments. But notably, two of the eight patients with low pre-treatment IL-1β expression had received Budesonide foam or Prednisone. Steroids are known to suppress inflammatory response, including IL-1β. Therefore, larger multicenter studies that also include other JAK inhibitors are warranted to provide greater generalizability and comparable efficacy data in human patients.*' (Line: #506-511).

Figure 7F

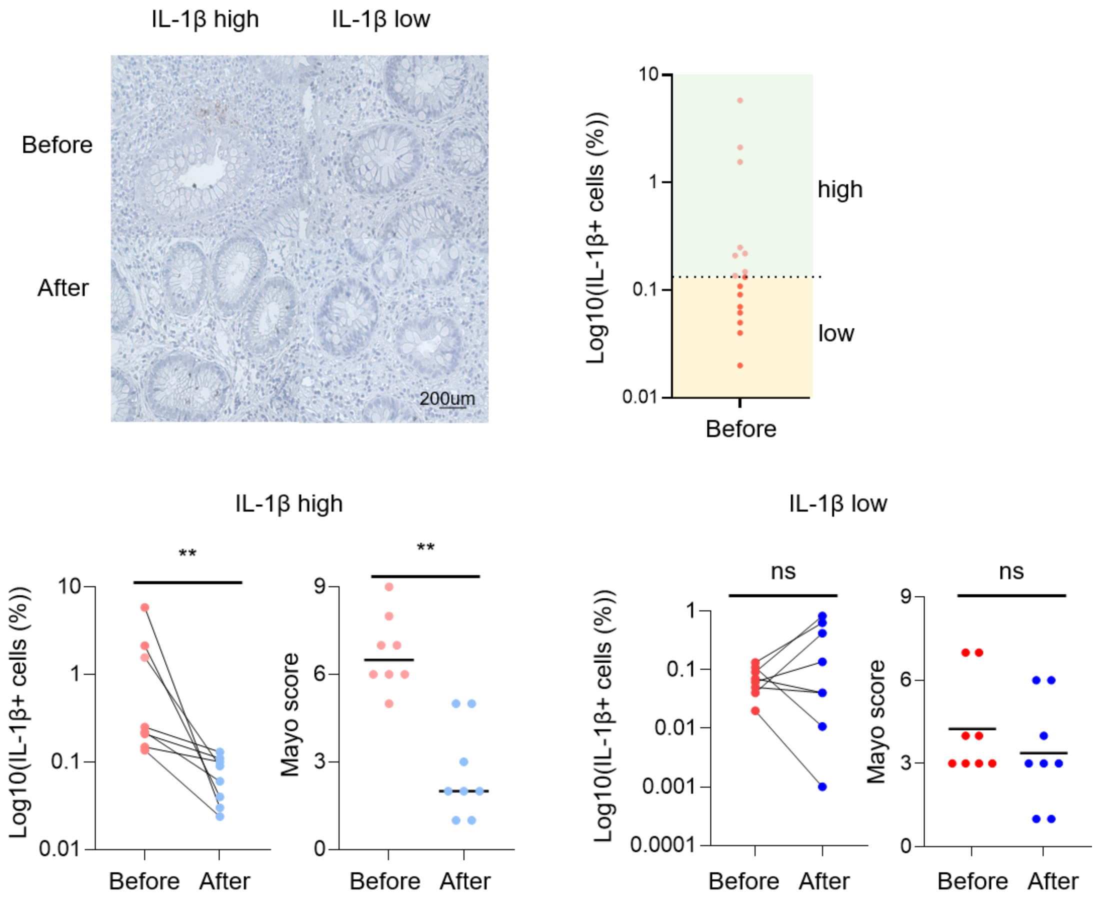

Figure 7. Upadacitinib downregulates IL-1β expression in UC patients. F) IHC of IL-1β in sigmoid of Upadacitinib-treated UC patients (n=16). Upper left: representative pictures showing IL-1β expression (brown). Upper right: quantified IL-1β expression before Upadacitinib treatment (baseline). Patients were grouped into IL-1β high or low groups based on the median of IL-1β expression among all individuals. The dotted line represents the median value. Lower left: quantified IL-1β expression and Mayo score of the patients in the “IL-1β high” group before and after Upadacitinib treatment. Lower right: quantified IL-1β expression and Mayo score of the patients in the “IL-1β low” group before and after Upadacitinib treatment. Bars represent mean value. Significance was analyzed using the Wilcoxon test, **p<0.01.

-While in-silico modeling suggests direct interaction with the catalytic site of NLRP3 (Figure 6E), direct experimental validation of this binding (e.g., by co-crystallization or detailed biochemical binding assays) would further support this specific mechanistic claim. It is worth considering whether this could be done.

We truly appreciated this point. In-silico modeling suggested a direct interaction with the catalytic site of NLRP3. To further test this, we performed a NLRP3 binding assay

using the NLRP3 NanoBRET™ Target Engagement (Teske et al., 2024) where MCC950 is included as a control. We first confirmed that JAK1 inhibitors can suppress IL-1β at the concentrations tested. Nevertheless, the results revealed no direct binding signals of all JAK inhibitors (Sup. Fig. 8A and B, also below), indicating that JAK1 inhibitors modulate NLRP3 indirectly.

In light of these data, we removed the in-silico modeling and included the results of the binding assay. We discussed the findings in the manuscript:

Results: '*However, it remained unclear, whether JAK1 inhibitors would bind directly to NLRP3 or if they regulate NLRP3 expression through indirect mechanisms. To address this, we assessed the ability of JAK1 inhibitors to bind NLRP3 using the NLRP3 NanoBRET™ Target Engagement assay with MCC950 as a positive control. As expected, JAK1 inhibitors reduced IL-1β production at the tested concentrations (Sup. Fig. 8A). However, the assay revealed no detectable direct binding of any JAK inhibitor to NLRP3 (Sup. Fig. 8B), indicating that JAK1 inhibitors modulate NLRP3 activity through indirect mechanisms.*' (Line: #275-281)

Methods: *NLRP3 NanoBRET™ Target Engagement utilized bioluminescent resonance energy transfer (BRET). Briefly, HEK293 cells were transfected with NLRP3-Nanoluc®fusion construct. With the addition of the substrate, the Nanoluc tag from NLRP3 emitted the signal (donor) at 450nm. The NLRP3 tracer, binding to the NACHT domain and labeled with a chemically reactive dye, was added in the culture together with JAK inhibitors and MCC950 at concentrations of 0.000508, 0.00152, 0.00457, 0.0137, 0.0412, 0.123, 0.37, 1.11, 3.33,10μM. Prior to BRET measurement, the Nanoluc substrate was added to HEK 293 cells. With the tracer binding to NLRP3, the donor light excited the tracer, emitting the BRET signal at 600nm (acceptor). Competition for the binding site by inhibitors displace the tracer, thereby decreasing the acceptor signal. Occupancy of NLRP3 by inhibitors was calculated based on the detected signals and formula provided in the manufacturer's protocol.*' (Line: #604-620)

Discussion: '*In previous studies, Patel et al. reported that Baricitinib, a JAK1/2 inhibitor, suppresses the NLRP3–caspase-1 axis, although the underlying mechanism remained unclear. Zhu et al. further demonstrated that Ruxolitinib, another JAK1/2 inhibitor, can indirectly inhibit NLRP3 activation by suppressing STAT3 in a murine cerebral ischemia model. Based on these findings, we examined STAT1 and STAT3 phosphorylation in lysates from DSS-induced colitis in mice and in human monocyte-derived macrophages treated with Tofacitinib, Upadacitinib, or Filgotinib. However, we did not detect any differences among the three inhibitors (Sup. Fig 8C and D). Given that*

Tofacitinib failed to downregulate inflammasome signaling, these data collectively suggest that STAT1 and STAT3 are unlikely to mediate the JAK1 inhibitor–induced reduction of IL-1β in this context.'

'We next examined whether the JAK1 pathway regulates the NLRP3-IL-1β axis by knocking down JAK1 or NLRP3, in human macrophages. Notably, JAK1 inhibitors continued to efficiently reduce IL-1β levels in JAK1-deficient macrophages, but not in NLRP3-deficient cells. These results indicate that JAK1 inhibitors suppress IL-1β independently of canonical JAK1 signaling.'

'To obtain a broader overview of transcriptional changes associated with IL-1β regulation, we analyzed genes involved in NLRP3 inflammasome activation and IL-1β processing that were downregulated by JAK1 inhibitors compared with Tofacitinib or control treatments in our single cell RNA-seq dataset. Pathway enrichment analysis revealed that many of the genes were involved in NF-κB and MAPK cascades. NF-κB indeed plays an important role in promoting the transcription of both NLRP3 and IL-1β (Hiscott et al, 1993, Liu et al., 2017). MAPK cascades are activated in parallel with NF-κB, and co-regulate the expression NLRP3 and IL-1β (Li et al., 2018, Wang et al., 2018). Consistent with this, we did observe that JAK1 inhibitors reduced both NLRP3 and IL1B transcript levels in macrophages (Figure 6A), suggesting that interference with NF-κB and/or MAPK signaling may contribute to their mechanism of action. Moreover, several additional genes previously reported to regulate NLRP3 and IL-1β expression, such as Hif1a (Tannahill et al., 2013) and Cebpb (Luo et al., 2022, Li et al., 2025) were also downregulated following JAK1 inhibition, which may also contribute to suppression of the inflammasome pathway.'

'Collectively, our data indicate that JAK inhibitors can modulate inflammasome pathway; however, it appears that many molecules and pathways might participate in the modulation, and the precise mechanisms appear to differ between compounds. Therefore, further studies are warranted to elucidate the detailed molecular pathways through which JAK1 inhibitors regulate the NLRP3-IL-1β axis.' (Line: #465-496)

Sup. Fig 8

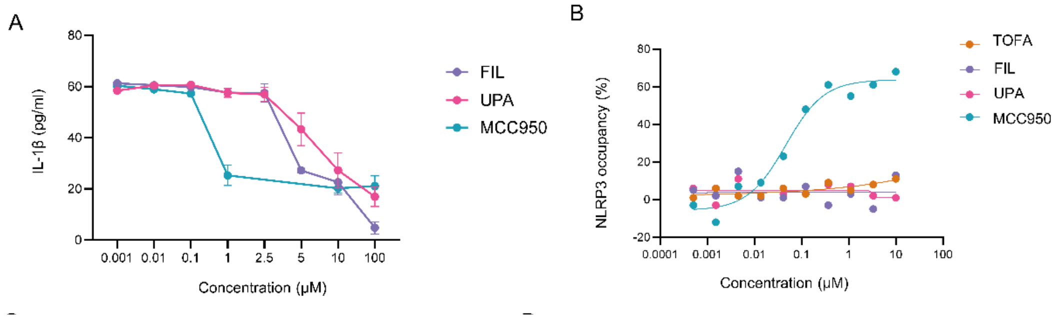

Supplementary Figure 8. JAK1 inhibitors control NLRP3 indirectly. A) Human monocyte-derived macrophages were activated with LPS in the presence of Filgotinib, Upadacitinib or MCC950 at indicated concentrations. IL-1β in the supernatant was measured by ELISA. **B)** Occupancy signal of NLRP3 in the presence of JAK inhibitors and MCC950 as measured using the NLRP3 NanoBRET™ Target Engagement.

-The authors of this study note that although other immune cells and signaling pathways may also be affected, they claim that macrophages are the primary target for IL-1β production. Further analyses investigating the influence of JAK1 inhibitors on other immune cell subsets and their contribution to the overall efficacy of the therapy could provide a more complete picture here.

We appreciated the suggestion from the reviewer. We performed an *in vitro* assay with human monocytes, monocyte-derived dendritic cells (DCs) and T cells in the presence of Tofacitinib, Upadacitinib and Filgotinib to assess the overall efficacy of the JAK inhibitors. '*We observed that all three JAK inhibitors equally suppressed the production of IL-12 and IL-6 in all populations, and IFN-γ from T cells, especially in PTPN2-variant patients (Sup. Fig 5A-E, see below). Owing to the low level of inflammasome activation induced by LPS, we were unable to reliably assess the effects of Upadacitinib and Filgotinib on IL-1β production in this setting (Sup. Fig. 5A and C). However, upon NLRP3-specific activation with MSU, both JAK1 inhibitors markedly reduced IL-1β production in dendritic cells and monocytes from IBD patients, regardless of PTPN2 genotype (Sup. Fig 5B and D). In contrast, Tofacitinib only modestly suppressed IL-1β production in monocytes and had no detectable effect in dendritic cells following MSU stimulation.*

Thus, Filgotinib and Upadacitinib—but not Tofacitinib—effectively inhibit IL-1β production and suppress inflammasome activation.' (Line: #231-241)

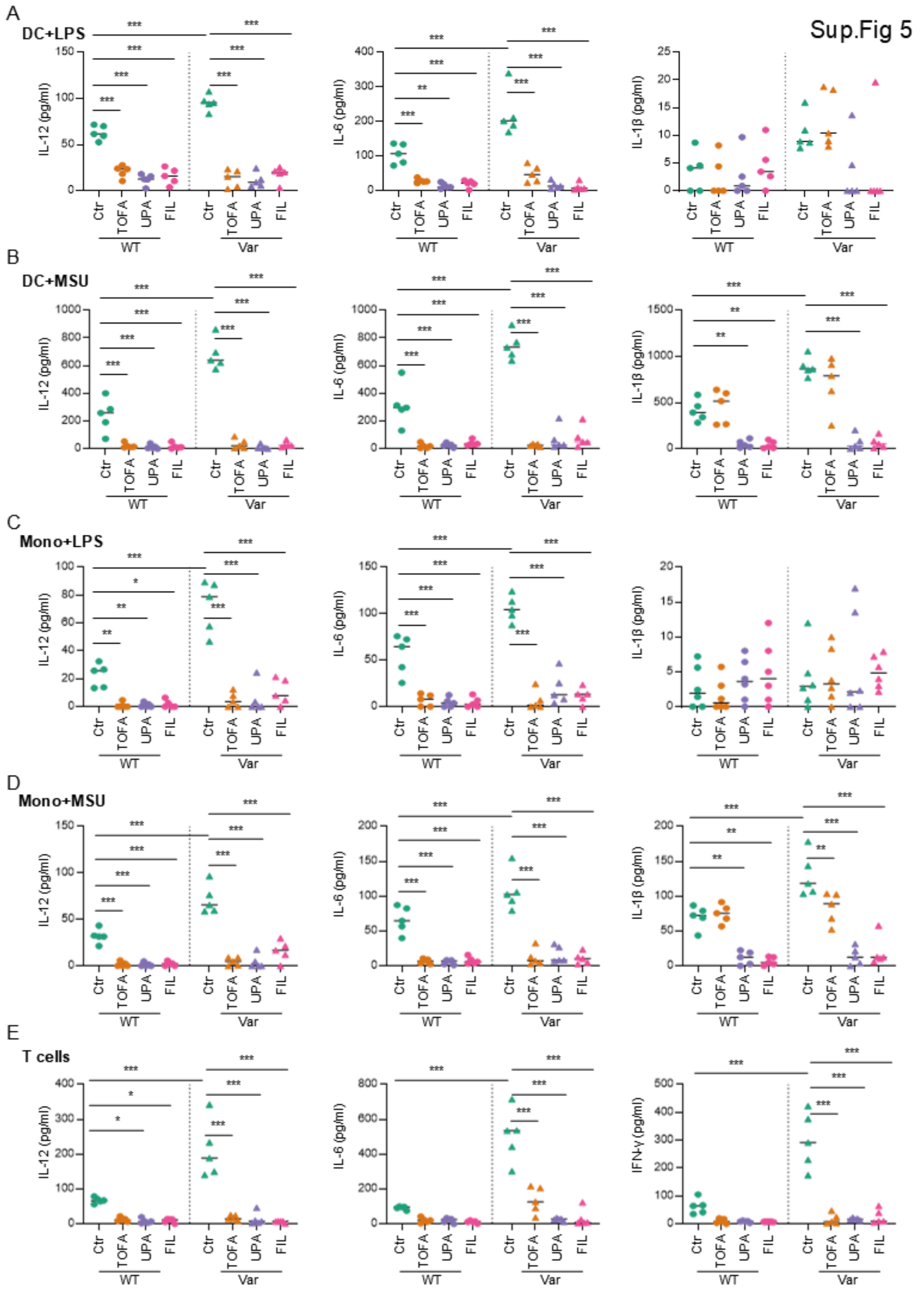

Supplementary Figure 5. Upadacitinib and Filgotinib inhibit cytokine production in monocytes, DCs and T cells. Monocytes and T cells were isolated from the peripheral blood of IBD patients that were either WT or variant for the loss-of-function SNP rs1893217 in the gene locus encoding PTPN2. Monocytes or monocyte-derivd DCs were then treated for 24h with LPS with or without futher activation with MSU crystals (150 ng/ml) for 6h. T cells were

activted by anti-CD3/anti-CD28 dynabeads. Cytokine levels in the supernatant for the indicated cytokines produced by **A-B)** DCs, **C-D)** monocytes and **E)** T cell. Every dot corresponds to one individual. Bars represent mean value, *$p<0.05$, **$p<0.01$, ANOVA with Holm-Sidak's multiple comparison test.

In addition to the recommendations, we included one more point in the discussion: '*Interestingly, our results demonstrate a robust suppression of IL-1β at a concentration of approximately 5 μM Upadacitinib in vitro (Sup. Fig 8A, presented under Point 3). Notably, a previous study suggested that colonic concentrations of Upadacitinib may fall within a similar range (Favaron et al, 2024), and findings from the study evaluating other JAK inhibitor further support this estimate (Verstockt et al., 2025). This is in line with our human data, which show a marked reduction of IL-1β in colonic tissue from patients who respond to Upadacitinib. Nevertheless, additional studies are warranted to directly quantify colonic Upadacitinib levels and to correlate these concentrations with IL-1β expression and clinical outcomes*.' (Line: #512-519)

This revised manuscript presents a very informative and well-conducted study that provides important insights into the different mechanisms of action of JAK inhibitors in ulcerative colitis. The authors have carefully addressed the reviewers' concerns, in particular by generating new data on the direct NLRP3-inhibiting effect of JAK1 inhibitors, a second experimental model, and the validation of these results using samples from human UC patients. The study is now in better shape. It could represent a valuable contribution to therapeutic options in the field of inflammatory bowel disease.

We thank the reviewer for the overall very positive assessment of our work and are grateful for the additional recommendations, which helped us further improve the manuscript.

Reviewer #4 (Remarks to the Author):

The authors have provided a very comprehensive rebuttal to the criticisms of the 3 original reviewers and performed a significant amount of additional work.

We thank the reviewer for the very positive assessment of our work.

To reviewers' comments:

Reviewer #3 (Remarks to the Author):

Many thanks to the authors. They have thoroughly addressed my points and I now agree with the manuscript.

We thank the reviewer for the very positive assessment of our work and also for the insightful suggestions provided during the revision process. We are pleased that the reviewer now agrees with the manuscript.

In addition, we revised our manuscript in accordance with the Author Checklist. Detailed responses to every point are listed in the completed Author Checklist. Briefly, the main changes include:

- As required, we included the animal model context in the title. Therefore, the title has been revised to "Indirect inhibition of the NLRP3-interleukin-1β axis contributes to the efficacy of JAK1 inhibitors in experimental colitis and human ulcerative colitis";
- Abstract has been revised to meet 150-word limit;
- Detailed statistical analyses have been updated in the figure legends, and exact *p*-values are reported in all the figures;
- Figures have been reformatted following the guidelines of *Nature Communications;*
- Data Availability section has been added after Methods.
